# National healthcare-associated infections surveillance programs – comparison between provincial and national case definitions in Canada

**DOI:** 10.1186/s12963-026-00508-y

**Published:** 2026-08-25

**Authors:** Etienne Poirier, Caroline Quach, Anne MacLaurin, Janie Coulombe

**Affiliations:** 1 Department of Microbiology, Infectious Diseases, and Immunology, Faculty of Medicine, University of Montreal, Montreal, Canada; 2 Research Center, CHU Sainte Justine, Montreal, Canada; 3 Infection Prevention & Control, CHU Sainte-Justine, Montreal, Canada; 4 Department of Pediatric Laboratory Medicine, CHU Sainte-Justine, Montreal, Canada; 5 Healthcare Excellence Canada, Ottawa, Canada; 6 Department of Mathematics and Statistics, Université de Montréal, 2920 Chemin de la Tour, Montreal, Québec, H3T 1J4 Canada

## Abstract

**Background:**

Provincial and national case definitions for healthcare-associated infections (HAI) differ. A key question is the extent to which these differences impact the estimated case proportions.

**Methods:**

Analyses focused on two infections: *Clostridioides difficile* infection (CDI) and methicillin-resistant *Staphylococcus aureus* (MRSA). After identifying divergent elements in definitions, a parametric simulation was conducted using a multivariate Bernoulli model to generate elements, incorporating assumed relationships between them and necessary sample sizes. A reference simulation was performed using the original proportions of elements as reported in the literature. This reference served as a baseline for comparison with simulations in which the proportions of discordant elements varied between provincial and national case definitions.

**Results:**

Nine discordant elements were identified for CDI and six for MRSA. Simulations showed comparability between provincial and national HAI proportions. The greatest differences between provincial and national proportions occurred for Newfoundland and Labrador (CDI) and Ontario (MRSA). These differences persisted even in the reference simulation.

**Conclusion:**

Although adopting a standardized case definition would simplify comparisons, provincial case definitions are generally comparable to the national definition. Results should be interpreted with care, especially when estimating case proportions under extreme values for some characteristics, as the joint distributions we used for generating simulated datasets did not necessarily exist nor represent target marginal proportions or associations between elements in some scenarios. To improve comparability, hospitals should calculate both the number of patients with positive CDI or MRSA results and their proportion of all elements used in case definitions to have a better picture of their patient demographics. This information could further help the Canadian Nosocomial Infection Surveillance Program (CNISP) to describe comparability across provinces.

**Supplementary Information:**

The online version contains supplementary material available at https://doi.org/10.1186/s12963-026-00508-y.

## Introduction

Healthcare-associated infections (HAIs) are a major public health issue worldwide [1]. Patients acquire these infections during a hospital stay or a healthcare episode, which means that they were not infected at the time of admission. In the United States, HAI costs amount to several billion dollars [2]. Several measures, including surveillance, are implemented to prevent these infections. Surveillance of HAIs in Canada occurs at two levels: provincial and national. Canadian hospitals may participate in voluntary surveillance at the national level, whereas they are required to follow guidelines for regional, provincial or territorial surveillance (e.g., which infection to monitor and the frequency of reporting data to the province). Even though some infections are monitored at both levels, national and provincial authorities have established their own surveillance programs with, at times, their own case definitions [3–6]. Since case definitions between each province and the federal government (Canadian Nosocomial Infection Surveillance Program, CNISP) are based on different elements, e.g., at least 72 h vs. 48 h of admission to be counted as HAI, exclusion vs. inclusion of children 1 year old or less for *C. difficile* surveillance, divergent HAI rates may be reported for the same population.

Advantages of a pan-Canadian surveillance program include benchmarking and risk-stratified incidence for specific populations such as burn units, neonatal, and pediatric units, for which the patient population and number of units is too small in each province. To be useful, this type of surveillance must be timely. To this end, harmonized provincial and national definitions would be essential. If harmonizing case definitions is impossible, they must be sufficiently concordant to allow for national comparison.

We have demonstrated a good concordance between case classification based on provincial definitions and those of the CNISP for methicillin-resistant *Staphylococcus aureus* (MRSA) and *Clostridoides difficile* infections (CDI) [7]. In this follow-up work, we aimed to assess whether the estimated provincial case proportions remained comparable when patient characteristics were varied across provinces. To do so, we performed a large simulation study using publicly available provincial surveillance data for CDI and MRSA bloodstream infections (BSI). That simulation evaluated how discrepancies between each provincial case definitions for MRSA BSI and CDI and those of CNISP impact HAI reported rates.

## Methods

### Research question

For MRSA BSI and CDI, what is the impact of discordant elements in case definitions, when comparing provincial and national rates? As a sub-question, what is the threshold at which an overexpression (or underexpression) of provincial characteristics results in provincial and national rates being no longer comparable?

### Setting and study design

The two most surveyed HAIs, CDI and MRSA BSI, were selected for this study. To participate in the study, provinces and territories must have a surveillance protocol for these healthcare-acquired infections, and surveillance reports must be publicly available. This information was available for eight provinces for CDI and six for MRSA [3, 5, 6, 8–19], giving us a global picture with a national reach. Alberta, New-Brunswick, Saskatchewan (for MRSA), and the three territories were excluded from the analyses because public reports were not available for these provinces and territories.

### Case definition

The most recent definitions for each province were retrieved from provincial websites in January 2024 [3, 5, 6, 8–20]. Using this information, we determined whether each profile (i.e., a patient with specific provincial characteristics) met the case definition for each province in the study. Table 1 lists the discordant elements for both infections that we identified across all case definitions for the Canadian provinces and CNISP. For the analysis of discrepancies, nine elements were created for CDI and six for MRSA (Table 2).

**Table 1:** Comparison of elements discrepancies between provincial and CNISP, CDI and MRSA

Province	Elements of provincial definition	Elements of the CNISP definition	Discrepancies
***Clostridioides difficile*** ** infections**			
British Columbia, Nova Scotia	If the patient’s medical chart was reviewed and the information about the frequency and consistency of diarrhea was not available, or only one or two liquid or loose stools was documented, the infection control practitioner must decide based on the patient’s other clinical manifestations and treatments or consult with the nurse or physician caring for the patient.	**Inclusion**: has diarrhea or fever, abdominal pain and/or ileus AND a laboratory confirmation of a positive toxin assay	Diarrhea
Newfoundland and Labrador	Recurrence of diarrhea within four weeks of a previous *C. difficile* infection episode	**Inclusion**: new episode of CDI which occurs greater than eight weeks after the diagnosis of a previous episode in the same patient	Recurrence between 4 to less than 8 weeks
Newfoundland and Labrador, Prince Edward Island	Patient 1 year old and less accepted	**Exclusion**: Any patients under 1 year of age.	Patient age of 1 year old and more
Nova Scotia, Ontario	Patients who were discharged in the previous 4 weeks and return to the ER or outpatient clinic with a new onset of CDI, but are not readmitted, are NOT included.	**Inclusion**: The patient presents with CDI symptoms at your ER or outpatient location AND the patient had been previously hospitalized at your healthcare facility and discharged within the previous 4 weeks	Patient admission
Prince Edward Island	Fever and abdominal pain AND a positive toxin assay or PCR cannot be used for a case confirmation	**Inclusion**: has diarrhea or fever, abdominal pain and/or ileus AND a laboratory confirmation of a positive toxin assay or positive polymerase chain reaction (PCR) for *C. difficile* toxin gene(s)	Fever and abdominal pain
Prince Edward Island	Hospitalization > 48 h before specimen collection	**Inclusion**: The patient’s CDI symptoms occur in your healthcare facility 3 or more days (or ≥ 72 h) after admission	Two days of admission
Saskatchewan	If information about the frequency and consistency of diarrhea is not available, a toxin-positive stool or positive PCR may be considered as a case.	**Inclusion**: Starting in 2017, we will no longer accept asymptomatic cases identified only by laboratory confirmation of a positive toxin assay or PCR for *C. difficile* (i.e. A patient must have diarrhea or fever, abdominal pain and/or ileus AND a laboratory confirmation of a positive toxin assay or PCR for *C. difficile* to be identified as having CDI)	Information about diarrhea available
**Methicillin-resistant** *** Staphylococcus aureus***			
Newfoundland and Labrador, Nova Scotia, Prince Edward Island	No precision about the mother MRSA status on admission (newborn)	The mother was NOT known to have MRSA on admission and there is no epidemiological reason to suspect that the mother was colonized prior to admission, even if the newborn is < 48 h of age	Mother status
Quebec	No precision on admission status	Patient must be admitted to the hospital	Patient admission
Quebec	Mental health, labour and delivery beds are excluded	No information about exclusion of these departments	Patient from Mental or mother and children department
Ontario	The infection was present at the time of admission but was related to a previous admission to the same facility within the last 72 h	**Inclusion**: Patient has been hospitalized in your facility in the last 7 days or up to 90 days depending on the source of infection	Previous admission within the last 3 days

**Table 2:** List of discordant elements and proportions of each at reference used for the parametric simulation, CDI and MRSA

Elements	Proportion of elements identified in literature and set for reference
***Clostridioides difficile*** ** infections**	
Two days of admission	7.2%
Three days of admission	49.3%
Fever and abdominal pain	6.3%
Diarrhea	84.0%
Admission	77.0%
One year old and more	98.5%
Recurrence between four and eight weeks	18.4%
Hospitalisation within four weeks	18.5%
Information on diarrhea available	83.0%
**Methicillin-resistant** *** Staphylococcus aureus***	
Three days of admission	34.0%
Mother status	5.5%
Admission	71.8%
Mental or mother and children patients	13.2%
Previous admission within the last 7 or up to 90 days	66.0%
Previous admission within the last 3 days	3.0%

### Simulations and cohort construction

We used parametric simulations to estimate HAI reported rates under varying patient characteristics. A parametric simulation is a technique that can be used to generate new data under specific structural assumptions. We used a multivariate Bernoulli model to generate all the elements with the assumed relationships between them and the sample sizes required. By *elements*, we refer to variables (i.e., patient characteristics) used in the case definition(s). For example, the case definition in Quebec states that to confirm a CDI case as HAI, the diagnosis must occur after three days of admission. It corresponds to the element *Three days of admission*. Table 1 lists the discordant elements for both infections that we identified across all case definitions for the Canadian provinces and CNISP. For the analysis of discrepancies, nine elements were created for CDI and six for MRSA BSI (Table 2).

To generate realistic data, relationships (i.e., associations) between elements used in the case definitions were estimated in the Quebec data in the form of odds ratios (OR). We retrieved the case databases that we had used in a previous study [7]: 100 CDI cases from the *Centre Hospitalier Universitaire Sainte-Justine* (CHUSJ, Montreal, Canada), a mother-child hospital, and 267 MRSA cases from the *McGill University Health Centre* (MUHC, Montreal, Canada), which also included pediatric patients. We estimated the OR for each pair of two elements (36 combinations for CDI and 15 for MRSA). Table S1 shows all the different combinations of elements. For example, the Quebec definition uses the elements *Three days of admission* and *Diarrhea*. An estimated OR greater than 1 indicates that if the element *Three days of admission* is present, the element *Diarrhea* is also more likely to be present. Conversely, with an OR lower than 1, the opposite is true.

To create provincial cohorts of patients with realistic proportions of characteristics, the original proportions of each element were found in the literature by reviewing articles where proportions of the elements were reported [21–29]. For example, in one study, 77.0% of patients with laboratory-confirmed *C. difficile* were admitted, while 84.0% presented with diarrhea [24]. The element proportions identified in the literature are presented in Table 2. When these proportions were not available in the literature, we used our case databases (CDI and MRSA) to determine them [7].

To determine the sample size for each province’s simulated data, we first found the number of nosocomial CDI and MRSA cases in the most recent provincial surveillance reports (July 2024) [30–38]. To calculate sample sizes with these findings, we used the proportion of cases meeting the nosocomial case definition from our previous study [7]. For instance, the latest surveillance report from Quebec indicated 1,335 HAI cases of CDI [39]. In our previous study, 36% of our Quebec laboratory-confirmed CDI cases met the CDI nosocomial case definition. Based on this information, the simulated sample size for Quebec was estimated at 3,708 (1,335/36%) laboratory-confirmed CDI cases, both HAI and not HAI.

Then, a multivariate Bernoulli model was parametrized to generate cohorts with the proportions of elements found from the literature (which we refer to as reference cohorts and results), simultaneously while preserving the relationships between elements from the estimated ORs, and the sample sizes described above, using the function *ObtainMultBinaryDist* in the R package *mipfp* [40]. The proportions of elements could further be varied to assess the effect of having populations with different characteristics. We used the same ORs to generate simulated data across all provinces so that these relationships (estimated from Quebec data) were assumed to be the same across provinces. In each simulation, we generated cohorts and calculated the proportion of infections meeting the case definition for each province and for CNISP and results were then averaged over the 500 simulations.

### Statistical analysis

A first series of simulations used the original proportions of elements found from the literature. The corresponding results served as reference, for both infections.

We calculated 95% confidence intervals (CI) of the proportion of HAIs by taking the 2.5% and 97.5% percentiles of case proportions across all simulations. These CI were used to compare proportions of cases between provinces and CNISP. We considered a difference in these proportions as statistically significant whenever the 95% confidence interval for the province and CNISP proportions of cases did not overlap. In result tables, we indicated whether the difference was statistically significant, and which of CNISP or the provincial definition led to the lowest or highest proportion. We also present in Supplementary material the absolute and relative differences between the proportions.

Statistical analyses were conducted in two parts. First, we evaluated the impact on proportions of cases, when the proportion of each element was varied one-by-one. In our analyses, we had each element vary from 5% to 95% in increments of 5%, by changing the parameters corresponding to the element proportions in the multivariate Bernoulli model, keeping the same ORs for the associations between elements. For example, we adjusted the element *Three days of admission* to 5% occurrence, then 10%, and so on up to 95%. Then, analyses were conducted in a different way that we refer to as the *provincial level*. Since provinces had more than one discordant element in their definitions, these analyses included variations of multiple elements simultaneously. As previously, 5% increments were used from 5% to 95%.

Finally, we compared HAI case proportions between each province and CNISP. Two types of comparison for proportions of patients meeting the case definition were conducted. The first comparison was between provinces with varying proportions of elements and CNISP, where for CNISP, we fixed the elements at their reference proportions. The second comparison, also focusing on the proportion of patients meeting the case definition, was conducted between each province with varying proportions of elements and CNISP, where CNISP was simulated using the same proportions of elements as the provinces. The rationale for doing so is that populations in each province could vary independently from one another, thus affecting national proportions (first analysis). However, characteristics of populations in each province could vary in sync with the national population (second analysis).

We did not perform any adjustment for multiple testing; statistical significance must be interpreted with caution and the overlap results be interpreted as descriptive.

### Ethics

This was an analysis of previously collected data. We only had access to the infection reference number and clinical information collected on the IPAC case report form. The overall research protocol was approved on July 8th, 2021 by the Centre de recherche du Centre Hospitalier Universitaire Sainte-Justine’s research ethics committee #2022–3382.

## Results

### Reference provincial cohorts

#### CDI

A total of nine discordant elements were considered to analyze the discrepancies between provincial and national case definitions (Table 2). Based on the elements included in our analyses, we conducted the first simulation using the original proportions of these elements (based on the literature) to determine the reference case proportions that we would use as benchmarks in the simulation studies (Table 3).

**Table 3:** Proportion of CDI and MRSA patients meeting provincial case definition at reference with the CI 95%, per province and per infection

Provinces	Proportion of profiles following provincial case definition	IC 95%	
		-	+
***Clostridioides difficile*** **infections**			
Prince Edward Island	52.4%	42.7%	62.6%
British Columbia	49.9%	48.0%	52.1%
Quebec	49.2%	47.3%	50.8%
Ontario	49.0%	47.5%	50.2%
Manitoba	49.2%	47.3%	51.1%
Saskatchewan	50.0%	46.0%	54.1%
Nova Scotia	49.6%	45.6%	53.3%
Newfoundland and Labrador	55.8%	49.4%	61.8%
CNISP	49.3%	48.2%	50.5%
**Methicillin-resistant** ***Staphylococcus aureus***			
British Columbia	58.1%	48.8%	67.9%
Manitoba	59.9%	50.0%	70.1%
Newfoundland and Labrador	59.9%	50.5%	69.7%
Nova Scotia	51.2%	34.8%	66.7%
Ontario	13.6%	7.4%	20.7%
Prince Edwars Island	54.8%	40.8%	69.7%
Quebec	66.8%	56.1%	77.0%
CNISP	58.1%	48.7%	67.6%

#### MRSA BSI

A total of six elements were identified (Table 2). Under these original proportions of the six elements, the first simulation was conducted to determine the reference proportions that would eventually be compared in simulation studies (Table 3). In this analysis, Ontario showed a proportion of nosocomial cases that was statistically lower than that of CNISP.

### Case definitions discrepancies

#### CDI

As the proportion of elements deviated from reference, either increasing or decreasing, the comparability of HAI proportions between the provinces and CNISP decreased (Table S2). For example, at reference, the proportion of element Two days of admission, was 7.2%. When the proportion of this element was 5%, all provincial proportion of case confirmation remained comparable with those from CNISP (green color). When the element proportion raised to 35%, the proportion of provincial confirmed cases of British Columbia became higher (color yellow) in comparison with CNISP at reference. This means that if 35% of patients with positive laboratory C. difficile infections were sampled at two days of admission, the confirmed cases of CDI would be higher in British Columbia compared to CNISP at reference (7.2%). When the proportion of the same element increased at 45%, the proportion of confirmed CDI cases was statistically lower in British Columbia, Manitoba, Ontario and Quebec than CNISP at reference. In this same situation (45% of patients sampled at two days of admission), the proportion of confirmed cases of Prince Edward Island were higher than CNISP at reference, and also if CNISP had the same proportion of patients sampled at two days of admission.

Provincial-level analyses, in which more than one element proportion was varied simultaneously, were conducted for three of the eight provinces in the study (Table S3). The other five provinces had fewer than two divergent elements compared to CNISP, making it impossible to perform comparisons at this level.

#### MRSA BSI

As with CDI, the greater the variation in the proportions of elements, the poorer the comparability between the provincial and CNISP HAI proportions (Table S4).

Provincial-level analyses were conducted only for Quebec and Ontario because other provinces had only one divergent element each, compared with CNISP definition (Table S5). For Quebec, the area where the comparison of HAI proportions of Quebec and CNISP was still acceptable (which includes the reference values) was substantial. For Ontario the comparability area was far from the reference threshold; an important change in the element proportions would have been needed to reach a comparability of HAI, otherwise HAI rates in Ontario were lower.

## Discussion

The analyses that were performed in this manuscript are critical for public health in Canada, where health is a provincial responsibility. Since provinces oversee the surveillance of healthcare-associated infections within their jurisdiction, they have adapted case definitions that account for their specific context. As a result, provincial case definitions exhibit differences among them. However, no study has been conducted to assess whether infection rates remain comparable despite these differences. The proposed analysis allows for the evaluation of differences between case definitions by testing various scenarios specific to Canada. Using their own data (sample size, OR between elements) and case definitions, other regions or countries with a similar political context could replicate these analyses according to their own reality.

### Case definitions discrepancies

#### CDI

When comparing HAI proportions at reference, proportions remained comparable between the provinces and CNISP. One reason that may explain these differences is the narrow CNISP confidence interval.

Furthermore, the variation in the proportion of discordant elements individually presents two possible scenarios. In the first, a substantial change in the proportion of a specific element is needed to affect the provincial-CNISP comparison (e.g., two days of admission or fever and abdominal pain). In the second, even a minor change in the element’s proportion is enough to make the province and CNISP non comparable (e.g., three days of admission and diarrhea). In the first scenario, the element would need to be significantly overrepresented among patients with laboratory-confirmed CDI for the province to no longer be comparable (e.g., 25% of two days of admission for all laboratory-confirmed CDI in PEI). This situation differs in the second scenario, where it is possible with slight variations in characteristics that provincial HAI proportion becomes non comparable (e.g., we would need 80% of patients presenting with diarrhea instead of 84% for all laboratory-confirmed CDI in Ontario to have a non comparable provincial rate with CNISP).

The element Information available on diarrhea shows an inconsistent comparison pattern. As its occurrence decreases, the proportion of nosocomial CDI positivity at the provincial level increases. This trend is counterintuitive, as the availability of information is necessary to confirm the presence or absence of diarrhea. This could be explained by the fact that no multivariate Bernoulli distribution exists, that would correspond to the parameters we set in simulations for the element proportions and the ORs between each pair of elements. Consequently, it is unclear whether the data returned by the multivariate distribution and the corresponding estimated case proportions are valid.

Provincial comparisons led to similar results: either the provincial comparability range (i.e. the range over which two proportions are not statistically different) with CNISP is very narrow (e.g., Newfoundland and Labrador and Prince Edward Island [two days of admission and age one year or older]), or this comparability range is very broad (e.g., Nova Scotia and Prince Edward Island [diarrhea, fever, and abdominal pain]).

For CDI, provinces and CNISP were comparable even under the different provincial case definitions. This statement is supported by the provincial comparisons with the CNISP at the reference point (Table 2). All provinces had infection proportions comparable to those reported by CNISP, despite differences in case definitions. We observed that divergences could occur with variations in certain elements. Adapting provincial case definitions to match those of CNISP would certainly be the simplest solution to ensure comparability. However, provinces that choose to keep their own case definition could still do so without markedly affecting their estimates of case proportions. Their comparison with CNISP data would be even more robust if they recorded the proportions of their discordant elements with the CNISP definition to ensure the quality of the comparison.

#### MRSA BSI

The MRSA analysis was more straightforward as most provinces had only one discordant element with the national case definition, except for two provinces that had two. The comparison between the proportions of HAI for provincial and CNISP was fairly robust to varying elements, and large variations in element proportions were required to impact comparability (except for Admission and Previous admission withing the last 7 or up to 90 days).

Quebec remained quite comparable to CNISP. However, the fact that surveillance is not conducted in mental health and mother-child departments could hinder comparison with the national data. Despite this difficulty, our results suggest that hospitals in Quebec can compare their proportion of HAI MRSA with those of CNISP using their own definition.

Ontario was the province with the most challenges in terms of comparability. When using even the reference proportions of elements, Ontario’s HAI proportion was not comparable to that of CNISP. Nearly all scenarios we assessed yielded a non-comparable proportion of HAI. This can be mainly explained by a more stringent case definition using the element Previous admission within the last 3 days, which is not used by any other province.

The analyses indicate that statistical comparisons of MRSA infections between provinces and CNISP appear valid, except for Ontario. To ensure the plausibility of the comparison, we recommend that hospitals document the proportion of each discordant element discussed in this work, in their data.

#### Validation

Prior to implementing the multivariate Bernoulli model, an initial analytical approach was explored by incorporating a weighting variable into the simulation. This weighting variable forced the model to preferentially select certain profiles over others, thereby allowing the marginal proportions of the target variables to be adjusted. However, this approach did not guarantee preservation of the associations between variables, as measured by the ORs. Consequently, the multivariate Bernoulli approach was adopted. This method allows the specification of desired marginal proportions while simultaneously accounting for the dependence structure among the variables.

The next objective was to determine whether the ‘forced’ associations were maintained when modifying the marginal proportions of the variables (that is, determining whether multivariate distributions existed, that were characterized by the ORs and marginal proportions forced into the model, when varying one element’s proportion from 5 to 95%). To assess this, a validation study was conducted using the simulated datasets. A total of 500 simulations, each consisting of 1,000 observations, were generated, and the behavior of both the ORs and the marginal proportions was evaluated. For both infections, the marginal proportions closely matched the predefined target values across all simulations. Likewise, most associations between elements were adequately preserved across the simulated scenarios, although preservation deteriorated for some ORs involving extreme marginal distributions or extreme estimated associations between variables were successfully preserved. Tables S10 to S13 in Supplemental files present statistics from the validation study. For instance, in Table S11 (CDI analysis), except for 3 cells out of 81, all average relative differences between theoretical values for the marginal proportions and the furthest marginal 95% CI limit for proportions in simulation studies were under 4.1%. That is, when varying the marginal proportion of an element from 5 to 95%, looking at the proportion of another element, 95% or more empirical proportions of that second element that were obtained in simulation studies were within 4.1% of their theoretical value (with each of the 81 cells representing the average taken across the 19 scenarios in which the first element varied from 5 to 95%). Results were similar for MRSA (Table S13) with all cells under 6.2%. Tables S10 and S12 present colored heatmaps showing the proportion out of 19 scenarios (representing 5 to 95% of an element varying) of situations in which the theoretical marginal proportion of OR was contained in the simulation 95% CI. Again, the marginal proportions were overall very well preserved while some ORs, especially those related to the variable One year or older, were hardly preserved. This is not surprising, as some estimated ORs in the original data exhibited extreme values (very low or very high ORs) which could lead to unrealistic data generation mechanisms and no existing multivariate distribution that matches our restrictions on proportions and ORs.

### Limitations

Since beginning this work, Nova Scotia has switched to using CNISP case definitions for their surveillance. This was not considered in our analysis.

The most significant limitation of this work is the lack of HAI representative data for each province. We had access to real-world Quebec data, which could have been used simultaneously with parametric simulations to perform the analysis. However, we decided not to use Quebec data directly since these patients were influenced by surveillance elements, such as the fact that surveillance in Quebec is limited to the population aged one year and older for CDI. Having few or no children under one year of age in our cohorts, based on Quebec data, would have underrepresented this population for provinces that accept them. Since a database with Canadian patients was not available, we used Quebec data, but only to estimate relationships (i.e., the ORs) between the elements to generate realistic parametric simulations. Future work could reproduce the analysis varying the assumptions on the ORs; for instance, we could assume in simulation studies that the different elements used in case definitions are independent (ORs all equal to 1) or allow departure from Quebec estimated ORs.

Second, the multiplicity of tests was not considered, so the results should be viewed with caution and the tests should not be interpreted as having a 5% nominal significance level. Third, as demonstrated in the analysis for the element Information on diarrhea available, some analyses with specific element proportions and ORs between elements led to spurious results and might have not corresponded to realistic multivariate distributions. The corresponding results should be interpreted with caution. Fourth, our results are highly dependent on the sample sizes used for each province. The results could be altered if these sizes vary from year to year. Fifth, marginal proportions were from different studies, meaning that the precision could not be standardized across elements and provinces. Moreover, because CNISP only makes surveillance for MRSA BSI, our results only apply for bloodstream infections. Results are not applicable for infections from all body parts. Finally, in simulations, for a small number of variables, the specified associations could not be preserved after data-generation. For CDI, it was particularly difficult to preserve the right association between the variable One year or older and other variables after multivariate data-generation. We think this is due to the small proportion of patients for whom that variable is coded as 0 in the original dataset (large proportion of 1 such that generated datasets can end up with 100% patients who are one year or older, leading to extreme ORs). For MRSA, the association between Mental or mother and children’s patients and Previous admission within the last 7 or up to 90 days was generally not preserved when varying the proportion of the Mother status variable. Therefore, results involving these variables should be interpreted with caution. Generally, it was challenging to preserve associations between variables when simulations were performed using extreme marginal proportions of elements (i.e., below 15% or above 85%). However, the practical impact of this limitation is expected to be limited, as such extreme distributions are unlikely to represent real-world conditions in the vast majority of scenarios.

## Conclusion

These results will be presented to the CNISP executive to support decision on harmonization of definitions across the country. We have shown that, under most circumstances, differences in HAI definitions still allow for results to be compared. Repeating the current exercise every 3–5 years, or whenever provinces or territories modify their surveillance definition, will allow for CNISP to know if and when discrepancies will arise.

## Electronic supplementary material

Below is the link to the electronic supplementary material.


Supplementary Material 1


## Data Availability

The Quebec patient data used to estimate some of the odds ratios are confidential and cannot be shared.
