# Supplementary material for: National healthcare-associated infections surveillance programs – comparison between provincial and national case definitions in Canada
Source: Popul Health Metr. 2026 Aug 25;24:53. doi: 10.1186/s12963-026-00508-y (PMC13536607; doi:10.1186/s12963-026-00508-y)
Supplement: Supplementary file 1 [file 12963_2026_508_MOESM1_ESM.docx]

**Supplemental files**

**Table S1-** Combinations of CDI and MRSA elements for the OR calculations

| ***Clostridioides* *difficile* infections** | | | | | | | | | |
| --- | --- | --- | --- | --- | --- | --- | --- | --- | --- |
| **Elements** | **2d** | **3d** | **Fap** | **Di** | **Ad** | **Oy** | **Rec** | **H4w** |  |
| **3d** | 2d-3d |  |  |  |  |  |  |  |  |
| **Fap** | 2d-Fap | 3d-Fap |  |  |  |  |  |  |  |
| **Di** | 2d-Di | 3d-Di | Fap-Di |  |  |  |  |  |  |
| **Ad** | 2d-Ad | 3d-Ad | Fap-Ad | Di-Ad |  |  |  |  |  |
| **Oy** | 2d-Oy | 3d-Oy | Fap-Oy | Di-Oy | Ad-Oy |  |  |  |  |
| **Rec** | 2d-Rec | 3d-Rec | Fap-Rec | Di-Rec | Ad-Rec | Oy-Rec |  |  |  |
| **H4w** | 2d-H4w | 3d-H4w | Fap-H4w | Di-H4w | Ad-H4w | Oy-H4w | Rec-H4w |  |  |
| **Inf** | 2d-Inf | 3d-Inf | Fap-Inf | Di-Inf | Ad-Inf | Oy-Inf | Rec-Inf | H4w-Inf |  |
| **Methicillin-resistant *Staphylococcus* *aureus*** | | | | | |  |  |  |  |
| **Elements** | **3d** | **Ms** | **Ad** | **Mmc** | **A7/90** |  |  |  |  |
| **Ms** | 3d-Ms |  |  |  |  |  |  |  |  |
| **Ad** | 3d-Ad | Ms-Ad |  |  |  |  |  |  |  |
| **Mmc** | 3d-Mmc | Ms-Mmc | Ad-Mmc |  |  |  |  |  |  |
| **A7/90** | 3d-A7/90 | Ms-A7/90 | Ad-A7/90 | Mmc-A7/90 |  |  |  |  |  |
| **Ad/3** | 3d-Ad/3 | Ms-Ad/3 | Ad-Ad/3 | Mmc-Ad/3 | A7/90-Ad/3 |  |  |  |  |

Abbreviations: 2d, two days of admission; 3d, three days of admission; A7/90, Previous admission withing the last 7 or up to 90 days; Ad, Admission; Ad/3, Previous admission within the last three days; Di, Diarrhea; Fap, Fever and abdominal pain; H4w, Hospitalization within the last four weeks; Inf, Information on diarrhea available; Mmc, Mental or mother and children patients; Ms, Mother status; Oy, One year or more; Rec, Recurrence between four and eight weeks

**Table S2-** Heatmap of the comparison of proportions of patients meeting case definition: provinces vs. CNISP, by element and per province, CDI

| **Two days of admission (Reference 7.2%)** | | | | | | | | | | | | | | | | | | | |
| --- | --- | --- | --- | --- | --- | --- | --- | --- | --- | --- | --- | --- | --- | --- | --- | --- | --- | --- | --- |
|  | 5% | 10% | 15% | 20% | 25% | 30% | 35% | 40% | 45% | 50% | 55% | 60% | 65% | 70% | 75% | 80% | 85% | 90% | 95% |
| BC |  |  |  |  |  | H | H |  | L | L | L | L | L | L | L | L | L | L | L |
| MB |  |  |  |  |  |  |  |  | L | L | L | L | L | L | L | L | L | L | L |
| NL |  |  |  |  |  | H |  |  |  |  |  |  | L | L | L | L | L | L | L |
| NS |  |  |  |  |  |  |  |  |  | L | L | L | L | L | L | L | L | L | L |
| ON |  |  |  |  |  |  |  |  | L | L | L | L | L | L | L | L | L | L | L |
| PEI |  |  |  |  | H | H | H | H | H | H | H | H | H | H | H | H | H | H | H |
| QC |  |  |  |  |  |  |  |  | L | L | L | L | L | L | L | L | L | L | L |
| SK |  |  |  |  |  |  |  |  |  |  | L | L | L | L | L | L | L | L | L |
| **Three days of admission (Reference 49.3%)** | | | | | | | | | | | | | | | | | | | |
|  | 5% | 10% | 15% | 20% | 25% | 30% | 35% | 40% | 45% | 50% | 55% | 60% | 65% | 70% | 75% | 80% | 85% | 90% | 95% |
| BC | L | L | L | L | L | L | L | L |  |  | H | H | H | H | H | H | H | H | H |
| MB | L | L | L | L | L | L | L | L | L |  | H | H | H | H | H | H | H | H | H |
| NL | L | L | L | L | L | L |  |  |  | H | H | H | H | H | H | H | H | H | H |
| NS | L | L | L | L | L | L | L | L |  |  |  | H | H | H | H | H | H | H | H |
| ON | L | L | L | L | L | L | L | L | L |  | H | H | H | H | H | H | H | H | H |
| PEI | L | L | L | L | L | L |  |  |  |  |  | H | H | H | H | H | H | H | H |
| QC | L | L | L | L | L | L | L | L | L |  | H | H | H | H | H | H | H | H | H |
| SK | L | L | L | L | L | L | L | L |  |  |  | H | H | H | H | H | H | H | H |
| **Fever and abdominal pain (Reference 6.3%)** | | | | | | | | | | | | | | | | | | | |
|  | 5% | 10% | 15% | 20% | 25% | 30% | 35% | 40% | 45% | 50% | 55% | 60% | 65% | 70% | 75% | 80% | 85% | 90% | 95% |
| BC |  |  |  |  |  |  |  |  |  |  |  |  |  |  |  |  |  |  |  |
| MB |  |  |  |  |  |  |  |  |  |  |  |  |  |  |  |  |  |  |  |
| NL |  |  |  |  |  |  |  | H |  |  |  |  |  |  |  |  | H | H | H |
| NS |  |  |  |  |  |  |  |  |  |  |  |  |  |  |  |  |  |  |  |
| ON |  |  |  |  |  |  |  |  |  |  |  |  |  |  |  |  |  |  |  |
| PEI |  |  |  |  |  |  |  |  |  |  |  |  |  |  |  |  |  |  |  |
| QC |  |  |  |  |  |  |  |  |  |  |  |  |  |  |  |  |  |  |  |
| SK |  |  |  |  |  |  |  |  |  |  |  |  |  |  |  |  |  |  |  |
| **Diarrhea (Reference 84.0%)** | | | | | | | | | | | | | | | | | | | |
|  | 5% | 10% | 15% | 20% | 25% | 30% | 35% | 40% | 45% | 50% | 55% | 60% | 65% | 70% | 75% | 80% | 85% | 90% | 95% |
| BC | L | L | L | L | L | L | L | L | L | L | L | L | L | L |  |  |  | H | H |
| MB | L | L | L | L | L | L | L | L | L | L | L | L | L | L | L |  |  |  | H |
| NL | L | L | L | L | L | L | L | L | L | L |  |  |  |  |  |  | H | H | H |
| NS | L | L | L | L | L | L | L | L | L | L | L | L | L | L |  |  |  |  | H |
| ON | L | L | L | L | L | L | L | L | L | L | L | L | L | L | L | L |  | H | H |
| PEI | L | L | L | L | L | L | L | L | L | L |  |  |  |  |  |  |  |  | H |
| QC | L | L | L | L | L | L | L | L | L | L | L | L | L | L | L |  |  | H | H |
| SK | L | L | L | L | L | L | L | L | L | L | L | L | L |  |  |  |  |  | H |
| **One year old and more (Reference 98.5%)** | | | | | | | | | | | | | | | | | | | |
|  | 5% | 10% | 15% | 20% | 25% | 30% | 35% | 40% | 45% | 50% | 55% | 60% | 65% | 70% | 75% | 80% | 85% | 90% | 95% |
| BC | L | L | L | L | L | L | L | L | L | L | L | L | L | L |  |  |  |  |  |
| MB | L | L | L | L | L | L | L | L | L | L | L | L | L | L | L |  |  |  |  |
| NL | L | L | L | L | L | L | L | L | L | L |  |  |  |  |  |  |  |  |  |
| NS | L | L | L | L | L | L | L | L | L | L | L | L |  |  |  |  |  |  |  |
| ON | L | L | L | L | L | L | L | L | L | L | L | L | L | L | L | L |  |  |  |
| PEI | H | H | H | H | H | H | H | H | H | H | H |  |  |  |  |  |  |  |  |
| QC | L | L | L | L | L | L | L | L | L | L | L | L | L | L | L |  |  |  |  |
| SK | L | L | L | L | L | L | L | L | L | L | L | L | L |  |  |  |  |  |  |
| **Admission (Reference 77.0%)** | | | | | | | | | | | | | | | | | | | |
|  | 5% | 10% | 15% | 20% | 25% | 30% | 35% | 40% | 45% | 50% | 55% | 60% | 65% | 70% | 75% | 80% | 85% | 90% | 95% |
| BC | LH | LH | LH | LH | L | L | L | L | L |  |  |  |  |  |  |  |  |  |  |
| MB | L | L | L | L | L | L | L | L | L |  |  |  |  |  |  |  |  |  |  |
| NL | L | L | L | L | L | L |  |  |  |  |  |  |  |  |  |  |  | H |  |
| NS | L | L | L | L | L | L | L | L | L |  |  |  |  |  |  |  |  |  |  |
| ON | L | L | L | L | L | L | L | L | L | L |  |  |  |  |  |  |  |  |  |
| PEI | L | L | L | L | L |  |  |  |  |  |  |  |  |  |  |  |  |  |  |
| QC | L | L | L | L | L | L | L | L | L |  |  |  |  |  |  |  |  |  |  |
| SK | LH | LH | L | L | L | L | L |  |  |  |  |  |  |  |  |  |  |  |  |
| **Four weeks recurrence (Reference 18.4%)** | | | | | | | | | | | | | | | | | | | |
|  | 5% | 10% | 15% | 20% | 25% | 30% | 35% | 40% | 45% | 50% | 55% | 60% | 65% | 70% | 75% | 80% | 85% | 90% | 95% |
| BC | H | H |  |  |  | L | L | L | L | L | L | L | L | L | L | L | L | L | L |
| MB | H |  |  |  |  | L | L | L | L | L | L | L | L | L | L | L | L | L | L |
| NL |  |  |  |  |  | H | H | H | H | H | H | H | H | H | H | H | H | H | H |
| NS | H |  |  |  |  | L | L | L | L | L | L | L | L | L | L | L | L | L | L |
| ON | H | H |  |  | L | L | L | L | L | L | L | L | L | L | L | L | L | L | L |
| PEI |  |  |  |  |  |  |  |  |  | L | L | L | L | L | L | L | L | L | L |
| QC | H | H |  |  | L | L | L | L | L | L | L | L | L | L | L | L | L | L | L |
| SK | H |  |  |  |  |  | L | L | L | L | L | L | L | L | L | L | L | L | L |
| **Hospitalisation within four weeks (Reference 18.5%)** | | | | | | | | | | | | | | | | | | | |
|  | 5% | 10% | 15% | 20% | 25% | 30% | 35% | 40% | 45% | 50% | 55% | 60% | 65% | 70% | 75% | 80% | 85% | 90% | 95% |
| BC |  |  |  |  |  |  | H | H | H | H | H | H | H | H | H | H | H | H | H |
| MB | L |  |  |  |  | H | H | H | H | H | H | H | H | H | H | H | H | H | H |
| NL |  |  |  | H | H | H | H | H | H | H | H | H | H | H | H | H | H | H | H |
| NS |  |  |  |  |  |  | H | H | H | H | H | H | H | H | H | H | H | H | H |
| ON | L | L |  |  |  | H | H | H | H | H | H | H | H | H | H | H | H | H | H |
| PEI |  |  |  |  |  |  |  |  |  | H | H | H | H | H | H | H | H | H | H |
| QC | L |  |  |  |  | H | H | H | H | H | H | H | H | H | H | H | H | H | H |
| SK |  |  |  |  |  |  | H | H | H | H | H | H | H | H | H | H | H | H | H |
| **Information on diarrhea available (Reference 83%)** | | | | | | | | | | | | | | | | | | | |
|  | 5% | 10% | 15% | 20% | 25% | 30% | 35% | 40% | 45% | 50% | 55% | 60% | 65% | 70% | 75% | **80%** | 85% | 90% | 95% |
| BC | L | L | L | L |  |  | H | H | H | H | H | H | H | H | H |  |  |  |  |
| MB | L | L | L | L |  |  | H | H | H | H | H | H | H | H | H |  |  |  |  |
| NL | L | L | L |  |  |  |  | H | H | H | H | H | H | H | H | H |  |  |  |
| NS | L | L | L | L |  |  | H | H | H | H | H | H | H | H | H |  |  |  |  |
| ON | L | L | L | L |  |  | H | H | H | H | H | H | H | H | H |  |  |  |  |
| PEI | L | L |  |  |  |  |  | H | H | H | H | H | H |  |  |  |  |  |  |
| QC | L | L | L | L |  |  | H | H | H | H | H | H | H | H | H |  |  |  |  |
| SK | L | L | L |  |  |  | H | H | H | H | H | H | H | H |  |  |  |  |  |

**Table S3-** Heatmap of the comparison of proportions of patients meeting case definition: provinces vs. CNISP, by province CDI

| **Nova Scotia** | **Information (Reference 83.0%)** | | | | | | | | | | | | | | | | | | | |
| --- | --- | --- | --- | --- | --- | --- | --- | --- | --- | --- | --- | --- | --- | --- | --- | --- | --- | --- | --- | --- |
| **Admission (Reference 77.0%)** |  | **5%** | **10%** | **15%** | **20%** | **25%** | **30%** | **35%** | **40%** | **45%** | **50%** | **55%** | **60%** | **65%** | **70%** | **75%** | **80%** | **85%** | **90%** | **95%** |
|  | **5%** | LH | LH | LH | LH | LH | L | L | L | L | L | L | L | L | L | L | L | L | L | L |
|  | **10%** | LH | L | L | L | L | L | L | L | L | L | L | L | L | L | L | L | L | L | L |
|  | **15%** | LH | L | L | L | L | L | L | L | L | L | L | L | L | L | L | L | L | L | L |
|  | **20%** | LH | L | L | L | L | L | L | L | L | L | L | L | L | L | L | L | L | L | L |
|  | **25%** | L | L | L | L | L | L | L | L | L | L |  | L | L | L | L | L | L | L | L |
|  | **30%** | L | L | L | L |  |  |  |  |  |  |  |  |  | L | L | L | L | L | L |
|  | **35%** | L | L | L | L |  |  |  |  |  |  |  |  |  |  | L | L | L | L | L |
|  | **40%** | L | L | L | L |  |  |  |  |  |  | H |  |  |  |  | L | L | L | L |
|  | **45%** | L | L | L | L |  |  |  |  | H | H | H | H |  |  |  |  | L | L | L |
|  | **50%** | L | L | L |  |  |  |  | H | H | H | H | H | H |  |  |  |  |  |  |
|  | **55%** | L | L | L |  |  |  |  | H | H | H | H | H | H | H |  |  |  |  |  |
|  | **60%** | L | L | L |  |  |  |  | H | H | H | H | H | H | H | H |  |  |  |  |
|  | **65%** | L | L | L |  |  |  |  | H | H | H | H | H | H | H | H |  |  |  |  |
|  | **70%** | L | L | L |  |  |  | H | H | H | H | H | H | H | H | H |  |  |  |  |
|  | **75%** | L | L | L | L |  |  | H | H | H | H | H | H | H | H | H |  |  |  |  |
|  | **80%** | L | L | L | L |  |  | H | H | H | H | H | H | H | H | H |  |  |  |  |
|  | **85%** | L | L | L | L |  |  | H | H | H | H | H | H | H | H |  |  |  |  |  |
|  | **90%** | L | L | L | L |  |  | H | H | H | H | H | H | H | H | H |  |  |  |  |
|  | **95%** | L | L | L | L |  |  | H | H | H | H | H | H | H | H | H |  |  |  |  |
| **Prince Edward Island** | **Fever and abdominal pain (Reference 6.3%)** | | | | | | | | | | | | | | | | | | | |
| **Diarrhea (Reference 84.0%)** |  | **5%** | **10%** | **15%** | **20%** | **25%** | **30%** | **35%** | **40%** | **45%** | **50%** | **55%** | **60%** | **65%** | **70%** | **75%** | **80%** | **85%** | **90%** | **95%** |
|  | **5%** | L | L | L | L | L | L | L | L | L | L | L | L | L | L | L | L | L | L | L |
|  | **10%** | L | L | L | L | L | L | L | L | L | L | L | L | L | L | L | L | L | L | L |
|  | **15%** | L | L | L | L | L | L | L | L | L | L | L | L | L | L | L | L | L | L | L |
|  | **20%** | L | L | L | L | L | L | L | L | L | L | L | L | L | L | L | L | L | L | L |
|  | **25%** | L | L | L | L | L | L | L | L | L | L | L | L | L | L | L | L | L | L | L |
|  | **30%** | L | L | L | L | L | L | L | L | L | L | L | L | L | L | L | L | L | L | L |
|  | **35%** | L | L | L | L | L | L | L | L | L | L | L | L | L | L | L | L | L | L | L |
|  | **40%** | L | L | L | L | L | L | L | L | L | L | L | L | L | L | L | L | L | L | L |
|  | **45%** | L | L | L | L | L | L | L | L | L | L | L | L | L | L | L | L | L | L | L |
|  | **50%** | L | L | L | L | L | L | L | L | L | L | L | L |  |  |  |  | L | L | L |
|  | **55%** |  |  |  |  |  |  |  |  |  |  |  |  |  |  |  |  |  |  |  |
|  | **60%** |  |  |  |  |  |  |  |  |  |  |  |  |  |  |  |  |  |  |  |
|  | **65%** |  |  |  |  |  |  |  |  |  |  |  |  |  |  |  |  |  |  |  |
|  | **70%** |  |  |  |  |  |  |  |  |  |  |  |  |  |  |  |  |  |  |  |
|  | **75%** |  |  |  |  |  |  |  |  |  |  |  |  |  |  |  |  |  |  |  |
|  | **80%** |  |  |  |  |  |  |  |  |  |  |  |  |  |  |  |  |  |  |  |
|  | **85%** |  |  |  |  |  |  |  |  |  |  |  |  |  |  |  |  |  |  |  |
|  | **90%** |  |  |  |  |  |  |  |  |  |  |  |  |  |  |  |  |  |  |  |
|  | **95%** |  |  | H |  |  |  |  |  |  |  |  |  |  |  |  |  | H | H | H |
| **Prince Edward Island** | **Two days of admission (Reference 7.2%)** | | | | | | | | | | | | | | | | | | | |
| **One year old and more (Reference 98.5%)** |  | **5%** | **10%** | **15%** | **20%** | **25%** | **30%** | **35%** | **40%** | **45%** | **50%** | **55%** | **60%** | **65%** | **70%** | **75%** | **80%** | **85%** | **90%** | **95%** |
|  | **5%** | H | H | H | H | **H** | **H** | **H** | **H** | **H** | **H** | **H** | **H** | **H** | H | H | H | H | H | H |
|  | **10%** | H | H | H | H | **H** | **H** | **H** | **H** | **H** | **H** | **H** | **H** | **H** | H | H | H | H | H | H |
|  | **15%** | H | H | H | H | **H** | **H** | **H** | **H** | **H** | **H** | **H** | **H** | **H** | **H** | H | H | H | H | H |
|  | **20%** | H | H | H | H | **H** | **H** | **H** | **H** | **H** | **H** | **H** | **H** | **H** | **H** | H | H | H | H | **H** |
|  | **25%** | H | H | H | H | **H** | **H** | **H** | **H** | **H** | **H** | **H** | **H** | **H** | **H** | H | H | H | H | H |
|  | **30%** | H | H | H | H | **H** | **H** | **H** | **H** | **H** | **H** | **H** | **H** | **H** | H | H | H | H | H | H |
|  | **35%** | H | H | H | H | **H** | **H** | **H** | **H** | **H** | **H** | **H** | **H** | **H** | **H** | H | H | H | H | H |
|  | **40%** | H | H | H | H | **H** | **H** | **H** | **H** | **H** | **H** | **H** | **H** | **H** | **H** | H | H | H | H | **H** |
|  | **45%** | H | H | H | H | **H** | **H** | **H** | **H** | **H** | **H** | **H** | **H** | **H** | H | H | H | H | H | H |
|  | **50%** | H | H | H | H | **H** | **H** | **H** | **H** | **H** | **H** | **H** | **H** | **H** | H | H | H | H | H | H |
|  | **55%** | H | H | H | H | **H** | **H** | **H** | **H** | **H** | **H** | **H** | **H** | **H** | H | H | H | H | H | **H** |
|  | **60%** |  | H | H | **H** | **H** | **H** | **H** | **H** | **H** | **H** | **H** | **H** | **H** | H | H | H | H | H | **H** |
|  | **65%** |  |  | H | H | **H** | **H** | **H** | **H** | **H** | **H** | **H** | **H** | **H** | H | H | H | H | H | **H** |
|  | **70%** |  |  |  | H | **H** | **H** | **H** | **H** | **H** | **H** | **H** | **H** | **H** | H | H | H | H | H | H |
|  | **75%** |  |  |  | H | **H** | **H** | **H** | **H** | **H** | **H** | **H** | **H** | H | H | H | H | H | H | **H** |
|  | **80%** |  |  |  |  | **H** | **H** | **H** | **H** | **H** | **H** | **H** | **H** | H | H | H | H | H | H | **H** |
|  | **85%** |  |  |  |  | **H** | **H** | **H** | **H** | **H** | **H** | **H** | **H** | H | H | H | H | H | H | **H** |
|  | **90%** |  |  |  |  | **H** | **H** | **H** | **H** | **H** | **H** | **H** | **H** | H | H | H | H | H | H | H |
|  | **95%** |  |  |  |  | **H** | **H** | **H** | **H** | **H** | **H** | **H** | **H** | **H** | H | H | H | H | H | **H** |

**Table S4-** Heatmap of the comparison of proportions of patients meeting case definition: provinces vs. CNISP, by element and by province, MRSA

| **Three days of admission (Reference 34.0%)** | | | | | | | | | | | | | | | | | | | |
| --- | --- | --- | --- | --- | --- | --- | --- | --- | --- | --- | --- | --- | --- | --- | --- | --- | --- | --- | --- |
|  | 5% | 10% | 15% | 20% | 25% | 30% | 35% | 40% | 45% | 50% | 55% | 60% | 65% | 70% | 75% | 80% | 85% | 90% | 95% |
| BC |  |  |  |  |  |  |  |  |  |  |  |  |  |  | H | H | H | H | H |
| MB |  |  |  |  |  |  |  |  |  |  |  |  |  |  |  | H | H | H | H |
| NL |  |  |  |  |  |  |  |  |  |  |  |  | H | H | H | H | H | H | H |
| NS |  |  |  |  |  |  |  |  |  |  |  |  |  |  |  |  |  | H | H |
| ON | L | L | L | L | L | L | L | L | L | L | L | L | L | L | L | L | L | L | L |
| PEI |  |  |  |  |  |  |  |  |  |  |  |  |  |  |  |  |  | H | H |
| QC |  |  |  |  |  |  |  |  |  |  |  |  |  |  |  |  |  | L | L |
| **Mother status (Reference 5.5%)** | | | | | | | | | | | | | | | | | | | |
|  | 5% | 10% | 15% | 20% | 25% | 30% | 35% | 40% | 45% | 50% | 55% | 60% | 65% | 70% | 75% | 80% | 85% | 90% | 95% |
| BC |  |  |  |  |  |  |  | L | L | L | L | L | L | L | L | L | L | L | L |
| MB |  |  |  |  |  |  |  |  |  | L | L | L | L | L | L | L | L | L | L |
| NL |  |  |  |  |  |  |  |  |  | H | H | LH | LH | LH | LH | LH | LH | LH | LH |
| NS |  |  |  |  |  |  |  |  |  |  |  |  |  |  | H | H | H | H | H |
| ON | L | L | L | L | L | L | L | L | L | L | L | L | L | L | L | L | L | L | L |
| PEI |  |  |  |  |  |  |  |  |  |  |  |  |  |  |  |  |  | L | L |
| QC |  |  |  |  |  |  |  |  |  |  | H | H | H | H | LH | L | L | L | L |
| **Admission (Reference 71,8%)** | | | | | | | | | | | | | | | | | | | |
|  | 5% | 10% | 15% | 20% | 25% | 30% | 35% | 40% | 45% | 50% | 55% | 60% | 65% | 70% | 75% | 80% | 85% | 90% | 95% |
| BC | L | L | L | L | L | L | L | L | L | L | L | L |  |  |  |  |  |  |  |
| MB | L | L | L | L | L | L | L | L | L | L | L | L |  |  |  |  |  |  |  |
| NL | L | L | L | L | L | L | L | L | L | L | L | L |  |  |  |  |  |  |  |
| NS | L | L | L | L | L | L | L | L |  |  |  |  |  |  |  |  |  |  |  |
| ON | L | L | L | L | L | L | L | L | L | L | L | L | L | L | L | L | L | L | L |
| PEI | L | L | L | L | L | L | L | L |  |  |  |  |  |  |  |  |  |  |  |
| QC | LH | LH | LH | LH | LH | H | H | H | H |  |  |  |  |  |  |  |  |  |  |
| **Patient from mental or mother and children department (Reference 13.2%)** | | | | | | | | | | | | | | | | | | | |
|  | 5% | 10% | 15% | 20% | 25% | 30% | 35% | 40% | 45% | 50% | 55% | 60% | 65% | 70% | 75% | 80% | 85% | 90% | 95% |
| BC |  |  |  |  |  |  |  |  |  |  | L | L | L | L | L | L |  |  |  |
| MB |  |  |  |  |  |  |  |  |  |  |  |  |  |  |  |  |  |  |  |
| NL |  |  |  |  |  |  |  |  |  |  |  |  |  |  |  |  |  |  |  |
| NS |  |  |  |  |  |  |  |  |  |  |  |  |  |  |  |  |  |  |  |
| ON | L | L | L | L | L | L | L | L | L | L | L | L | L | L | L | L | L | L | L |
| PEI |  |  |  |  |  |  |  |  |  |  |  |  |  |  |  |  |  |  |  |
| QC |  |  | L | L | L | L | L | L | L | L | L | L | L | L | L | L | L | L | L |
| **Previous admission within the last 7 or up to 90 days (Reference 66.0%)** | | | | | | | | | | | | | | | | | | | |
|  | 5% | 10% | 15% | 20% | 25% | 30% | 35% | 40% | 45% | 50% | 55% | 60% | 65% | 70% | 75% | 80% | 85% | 90% | 95% |
| BC | L | L | L | L | L | L | L | L | L | L | L | L |  |  |  |  |  |  |  |
| MB | L | L | L | L | L | L | L | L | L | L | L |  |  |  |  |  |  |  |  |
| NL | L | L | L | L | L | L | L | L | L | L | L |  |  |  |  |  |  |  |  |
| NS | L | L | L | L | L | L |  |  |  |  |  |  |  |  |  |  |  |  |  |
| ON | L | L | L | L | L | L | L | L | L | L | L | L | L | L | L | L | L | L | L |
| PEI | L | L | L | L | L | L |  |  |  |  |  |  |  |  |  |  |  |  |  |
| QC | L | L | L | L | L | L | L | L | L | L | L | L | L |  |  |  |  | H | H |
| **Previous admission within the last 3 days (Reference 3.0%)** | | | | | | | | | | | | | | | | | | | |
|  | 5% | 10% | 15% | 20% | 25% | 30% | 35% | 40% | 45% | 50% | 55% | 60% | 65% | 70% | 75% | 80% | 85% | 90% | 95% |
| BC |  |  |  |  |  |  |  |  |  |  |  |  |  | H | H | H | H | H |  |
| MB |  |  |  |  |  |  |  |  |  |  |  |  |  | H |  |  |  |  |  |
| NL |  |  |  |  |  |  |  |  |  |  |  |  |  | H | H | H | H | H |  |
| NS |  |  |  |  |  |  |  |  |  |  |  |  |  |  |  |  |  |  |  |
| ON | L | L | L | L | L | L | L | L | L | L | L | L |  | H | H | H | H | H |  |
| PEI |  |  |  |  |  |  |  |  |  |  |  |  |  |  |  |  |  |  |  |
| QC |  |  |  |  |  |  |  |  |  |  |  |  |  |  |  |  | H | H | H |

**Table S5-** Heatmap of the comparison of proportions of patients meeting case definition: provinces vs. CNISP, by province MRSA

| **Quebec** | **Patient from mental or mother and children department (Reference 13.2%)** | | | | | | | | | | | | | | | | | | | |
| --- | --- | --- | --- | --- | --- | --- | --- | --- | --- | --- | --- | --- | --- | --- | --- | --- | --- | --- | --- | --- |
| **Admission (Reference 71.8%)** |  | **5%** | **10%** | **15%** | **20%** | **25%** | **30%** | **35%** | **40%** | **45%** | **50%** | **55%** | **60%** | **65%** | **70%** | **75%** | **80%** | **85%** | **90%** | **95%** |
|  | **5%** | LH | LH | LH | LH | LH | LH | LH | LH | LH | LH | LH | L | L | L | L | L | L | L | L |
|  | **10%** | LH | LH | LH | LH | LH | LH | LH | LH | L | L | L | L | L | L | L | L | L | L | L |
|  | **15%** | H | LH | LH | LH | LH | LH | L | L | L | L | L | L | L | L | L | L | L | L | L |
|  | **20%** | H | LH | LH | LH | LH | LH | L | L | L | L | L | L | L | L | L | L | L | L | L |
|  | **25%** | H | H | LH | LH | LH | LH | L | L | L | L | L | L | L | L | L | L | L | L | L |
|  | **30%** | H | H | H | LH | LH | LH | L | L | L | L | L | L | L | L | L | L | L | L | L |
|  | **35%** | H | H | H | H | H | H |  |  |  | L | L | L | L | L | L | L | L | L | L |
|  | **40%** | H | H | H | H | H |  |  |  |  | L | L | L | L | L | L | L | L | L | L |
|  | **45%** | H | H | H | H |  |  |  |  |  |  | L | L | L | L | L | L | L | L | L |
|  | **50%** | H | H | H |  |  |  |  |  |  |  | L | L | L | L | L | L | L | L | L |
|  | **55%** | H | H |  |  |  |  |  |  |  |  | L | L | L | L | L | L | L | L | L |
|  | **60%** | H |  |  |  |  |  |  |  |  |  | L | L | L | L | L | L | L | L | L |
|  | **65%** |  |  |  |  |  |  |  |  |  |  | L | L | L | L | L | L | L | L | L |
|  | **70%** |  |  |  |  |  |  |  |  |  |  | L | L | L | L | L | L | L | L | L |
|  | **75%** |  |  |  |  |  |  |  |  |  | L | L | L | L | L | L | L | L | L | L |
|  | **80%** |  |  |  |  |  |  |  | L | L | L | L | L | L | L | L | L | L | L | L |
|  | **85%** |  |  |  |  |  |  |  | L | L | L | L | L | L | L | L | L | L | L | L |
|  | **90%** |  |  |  |  |  |  | L | L | L | L | L | L | L | L | L | L | L | L | L |
|  | **95%** |  |  |  |  |  | L | L | L | L | L | L | L | L | L | L | L | L | L | L |
| **Ontario** | **Previous admission within the last 3 days (Reference 3.0%)** | | | | | | | | | | | | | | | | | | | |
| **Previous admission within the last 7 or up to 90 days (Reference 66.0%)** |  | **5%** | **10%** | **15%** | **20%** | **25%** | **30%** | **35%** | **40%** | **45%** | **50%** | **55%** | **60%** | **65%** | **70%** | **75%** | **80%** | **85%** | **90%** | **95%** |
|  | **5%** | L |  |  |  |  |  |  |  |  |  |  |  |  |  |  |  |  |  |  |
|  | **10%** | L | L |  |  |  |  |  |  |  |  |  |  |  |  |  |  |  |  |  |
|  | **15%** | L | L | L |  |  |  |  |  |  |  |  |  |  |  |  |  |  |  |  |
|  | **20%** | L | L | L | L |  |  |  |  |  |  |  |  |  |  |  |  |  |  |  |
|  | **25%** | L | L | L | L | L |  |  |  |  |  |  |  |  |  |  |  |  |  |  |
|  | **30%** | L | L | L | L | L | L |  |  |  |  |  |  |  |  |  |  |  |  |  |
|  | **35%** | L | L | L | L | L | L | L |  |  |  |  |  |  |  |  |  |  |  |  |
|  | **40%** | L | L | L | L | L | L | L | L |  |  |  |  |  |  |  |  |  |  |  |
|  | **45%** | L | L | L | L | L | L | L | L | L |  |  |  |  |  |  |  |  |  |  |
|  | **50%** | L | L | L | L | L | L | L | L | L | L |  |  |  |  |  |  |  |  |  |
|  | **55%** | L | L | L | L | L | L | L | L | L | L | L |  |  |  |  |  |  |  |  |
|  | **60%** | L | L | L | L | L | L | L | L | L | L | L | L |  |  |  |  |  |  |  |
|  | **65%** | L | L | L | L | L | L | L | L | L | L | L | L |  |  |  |  |  |  |  |
|  | **70%** | L | L | L | L | L | L | L | L | L | L | L | L | L |  |  |  |  |  |  |
|  | **75%** | L | L | L | L | L | L | L | L | L | L | L | L | L |  | H |  |  |  |  |
|  | **80%** | L | L | L | L | L | L | L | L | L | L | L | L | L |  | H | H |  |  |  |
|  | **85%** | L | L | L | L | L | L | L | L | L | L | L | L | L |  | H | H | H |  |  |
|  | **90%** | L | L | L | L | L | L | L | L | L | L | L | L | L |  | H | H | H | H |  |
|  | **95%** | L | L | L | L | L | L | L | L | L | L | L | L | L |  |  |  |  |  |  |

**Table S6-** Distribution of absolute and relative differences of proportions of patients meeting case definition: provinces with varying proportions of elements vs. elements fixed at their reference proportions for CNISP, by province CDI

| **Two days of admission (Reference 7.2%)** | | | | | | | | | | | | | | | | | | | | | | | | | | | | | | | | | | | | | | | | | | | | | | | | | | | | | | | | | | | | | | | | | | | | | | | | | | | | | | | | | | | | | | | | | | | | | | | | | | | | | | | | | | | | | | | | | | | | | | | | | | | | | | | | | | | | | | | | | | | | | | | | | | | | | | | | | | | | | | | | | | | | | | | | | | | | | | | | | |  |
| --- | --- | --- | --- | --- | --- | --- | --- | --- | --- | --- | --- | --- | --- | --- | --- | --- | --- | --- | --- | --- | --- | --- | --- | --- | --- | --- | --- | --- | --- | --- | --- | --- | --- | --- | --- | --- | --- | --- | --- | --- | --- | --- | --- | --- | --- | --- | --- | --- | --- | --- | --- | --- | --- | --- | --- | --- | --- | --- | --- | --- | --- | --- | --- | --- | --- | --- | --- | --- | --- | --- | --- | --- | --- | --- | --- | --- | --- | --- | --- | --- | --- | --- | --- | --- | --- | --- | --- | --- | --- | --- | --- | --- | --- | --- | --- | --- | --- | --- | --- | --- | --- | --- | --- | --- | --- | --- | --- | --- | --- | --- | --- | --- | --- | --- | --- | --- | --- | --- | --- | --- | --- | --- | --- | --- | --- | --- | --- | --- | --- | --- | --- | --- | --- | --- | --- | --- | --- | --- | --- | --- | --- | --- | --- | --- | --- | --- | --- | --- | --- | --- | --- | --- | --- | --- | --- | --- | --- | --- | --- | --- | --- | --- | --- | --- | --- | --- | --- | --- | --- | --- | --- | --- | --- | --- | --- | --- | --- | --- |
|  | | | | | | | | | | 5% | | | | | | | | | 10% | | | | | | | | | 15% | | | | | | | | | 20% | | | | | | | | | 25% | | | | | | | | | | 30% | | | | | | | | | 35% | | | | | | | | | 40% | | | | | | | | | 45% | | | | | | | | | 50% | | | | | | | | | 55% | | | | | | | | | 60% | | | | | | | | | 65% | | | | | | | | | 70% | | | | | | | | | 75% | | | | | | | | | 80% | | | | | | | | 85% | | | | | | | | 90% | | | | | | | | 95% | | | | | | | |  |
| BC | | | | | | | | | | 1%; 1% | | | | | | | | | 1%; 1% | | | | | | | | | 1%; 2% | | | | | | | | | 1%; 2% | | | | | | | | | 2%; 4% | | | | | | | | | | 4%; 7% | | | | | | | | | 4%; 8% | | | | | | | | | 1%; 1% | | | | | | | | | -4%; -8% | | | | | | | | | -6%; -12% | | | | | | | | | -9%; -18% | | | | | | | | | -11%; -23% | | | | | | | | | -14%; -29% | | | | | | | | | -19%; -38% | | | | | | | | | -24%; -48% | | | | | | | | | -27%; -55% | | | | | | | | -29%; -58% | | | | | | | | -30%; -61% | | | | | | | | -31%; -63% | | | | | | | |  |
| MB | | | | | | | | | | 0%; 0% | | | | | | | | | 0%; 0% | | | | | | | | | 0%; 1% | | | | | | | | | 0%; 1% | | | | | | | | | 1%; 2% | | | | | | | | | | 2%; 4% | | | | | | | | | 2%; 4% | | | | | | | | | -1%; -3% | | | | | | | | | -5%; -11% | | | | | | | | | -8%; -15% | | | | | | | | | -10%; -21% | | | | | | | | | -13%; -26% | | | | | | | | | -16%; -32% | | | | | | | | | -20%; -40% | | | | | | | | | -25%; -51% | | | | | | | | | -28%; -57% | | | | | | | | -30%; -60% | | | | | | | | -31%; -63% | | | | | | | | -32%; -65% | | | | | | | |  |
| NL | | | | | | | | | | 6%; 13% | | | | | | | | | 7%; 13% | | | | | | | | | 7%; 14% | | | | | | | | | 7%; 14% | | | | | | | | | 7%; 15% | | | | | | | | | | 8%; 15% | | | | | | | | | 7%; 13% | | | | | | | | | 2%; 4% | | | | | | | | | -2%; -3% | | | | | | | | | -4%; -9% | | | | | | | | | -6%; -11% | | | | | | | | | -8%; -16% | | | | | | | | | -12%; -23% | | | | | | | | | -16%; -33% | | | | | | | | | -22%; -44% | | | | | | | | | -25%; -51% | | | | | | | | -26%; -52% | | | | | | | | -26%; -53% | | | | | | | | -26%; -53% | | | | | | | |  |
| NS | | | | | | | | | | 0%; 0% | | | | | | | | | 0%; 1% | | | | | | | | | 1%; 1% | | | | | | | | | 1%; 1% | | | | | | | | | 2%; 4% | | | | | | | | | | 4%; 7% | | | | | | | | | 4%; 8% | | | | | | | | | 1%; 1% | | | | | | | | | -4%; -8% | | | | | | | | | -6%; -12% | | | | | | | | | -9%; -18% | | | | | | | | | -11%; -23% | | | | | | | | | -15%; -29% | | | | | | | | | -19%; -38% | | | | | | | | | -24%; -49% | | | | | | | | | -27%; -55% | | | | | | | | -29%; -58% | | | | | | | | -30%; -61% | | | | | | | | -31%; -63% | | | | | | | |  |
| ON | | | | | | | | | | 0%; -1% | | | | | | | | | 0%; 0% | | | | | | | | | 0%; 0% | | | | | | | | | 0%; 0% | | | | | | | | | 1%; 2% | | | | | | | | | | 2%; 4% | | | | | | | | | 2%; 4% | | | | | | | | | -1%; -2% | | | | | | | | | -5%; -11% | | | | | | | | | -8%; -16% | | | | | | | | | -10%; -21% | | | | | | | | | -13%; -26% | | | | | | | | | -16%; -32% | | | | | | | | | -20%; -40% | | | | | | | | | -25%; -51% | | | | | | | | | -28%; -58% | | | | | | | | -30%; -60% | | | | | | | | -31%; -63% | | | | | | | | -32%; -65% | | | | | | | |  |
| PEI | | | | | | | | | | 2%; 4% | | | | | | | | | 5%; 10% | | | | | | | | | 8%; 16% | | | | | | | | | 10%; 19% | | | | | | | | | 14%; 29% | | | | | | | | | | 18%; 36% | | | | | | | | | 20%; 41% | | | | | | | | | 20%; 41% | | | | | | | | | 19%; 38% | | | | | | | | | 19%; 39% | | | | | | | | | 19%; 38% | | | | | | | | | 18%; 35% | | | | | | | | | 15%; 31% | | | | | | | | | 14%; 29% | | | | | | | | | 12%; 24% | | | | | | | | | 10%; 20% | | | | | | | | 10%; 19% | | | | | | | | 12%; 24% | | | | | | | | 16%; 33% | | | | | | | |  |
| QC | | | | | | | | | | 0%; 0% | | | | | | | | | 0%; 0% | | | | | | | | | 0%; 1% | | | | | | | | | 0%; 1% | | | | | | | | | 1%; 2% | | | | | | | | | | 2%; 4% | | | | | | | | | 2%; 4% | | | | | | | | | -1%; -2% | | | | | | | | | -5%; -11% | | | | | | | | | -8%; -16% | | | | | | | | | -10%; -21% | | | | | | | | | -13%; -26% | | | | | | | | | -16%; -32% | | | | | | | | | -20%; -40% | | | | | | | | | -25%; -51% | | | | | | | | | -28%; -57% | | | | | | | | -30%; -60% | | | | | | | | -31%; -63% | | | | | | | | -32%; -65% | | | | | | | |  |
| SK | | | | | | | | | | 1%; 1% | | | | | | | | | 1%; 1% | | | | | | | | | 1%; 2% | | | | | | | | | 1%; 2% | | | | | | | | | 2%; 4% | | | | | | | | | | 4%; 7% | | | | | | | | | 4%; 9% | | | | | | | | | 1%; 2% | | | | | | | | | -4%; -8% | | | | | | | | | -6%; -12% | | | | | | | | | -9%; -18% | | | | | | | | | -11%; -23% | | | | | | | | | -15%; -30% | | | | | | | | | -19%; -38% | | | | | | | | | -24%; -48% | | | | | | | | | -27%; -55% | | | | | | | | -28%; -58% | | | | | | | | -30%; -60% | | | | | | | | -31%; -63% | | | | | | | |  |
| **Three days of admission (Reference 49.3%)** | | | | | | | | | | | | | | | | | | | | | | | | | | | | | | | | | | | | | | | | | | | | | | | | | | | | | | | | | | | | | | | | | | | | | | | | | | | | | | | | | | | | | | | | | | | | | | | | | | | | | | | | | | | | | | | | | | | | | | | | | | | | | | | | | | | | | | | | | | | | | | | | | | | | | | | | | | | | | | | | | | | | | | | | | | | | | | | | |  |  |
|  | | | | | | | | | 5% | | | | | | | | | 10% | | | | | | | | | 15% | | | | | | | | | 20% | | | | | | | | | 25% | | | | | | | | | | 30% | | | | | | | | | 35% | | | | | | | | | 40% | | | | | | | | | 45% | | | | | | | | 50% | | | | | | | | 55% | | | | | | | | | 60% | | | | | | | | | | 65% | | | | | | | | | | 70% | | | | | | | | | 75% | | | | | | | | | 80% | | | | | | | | 85% | | | | | | | | 90% | | | | | | | | 95% | | | | | | | |  |  |
| BC | | | | | | | | | -31%; -64% | | | | | | | | | -28%; -57% | | | | | | | | | -24%; -49% | | | | | | | | | -21%; -42% | | | | | | | | | -17%; -35% | | | | | | | | | | -13%; -27% | | | | | | | | | -10%; -20% | | | | | | | | | -6%; -12% | | | | | | | | | -3%; -5% | | | | | | | | 1%; 2% | | | | | | | | 5%; 10% | | | | | | | | | 8%; 17% | | | | | | | | | | 12%; 24% | | | | | | | | | | 15%; 31% | | | | | | | | | 18%; 37% | | | | | | | | | 22%; 44% | | | | | | | | 25%; 50% | | | | | | | | 28%; 56% | | | | | | | | 32%; 65% | | | | | | | |  |  |
| MB | | | | | | | | | -32%; -64% | | | | | | | | | -28%; -57% | | | | | | | | | -25%; -50% | | | | | | | | | -21%; -42% | | | | | | | | | -17%; -35% | | | | | | | | | | -14%; -28% | | | | | | | | | -10%; -20% | | | | | | | | | -7%; -13% | | | | | | | | | -3%; -6% | | | | | | | | 1%; 1% | | | | | | | | 4%; 8% | | | | | | | | | 7%; 15% | | | | | | | | | | 11%; 21% | | | | | | | | | | 14%; 28% | | | | | | | | | 16%; 32% | | | | | | | | | 18%; 37% | | | | | | | | 22%; 45% | | | | | | | | 25%; 51% | | | | | | | | 30%; 60% | | | | | | | |  |  |
| NL | | | | | | | | | -28%; -56% | | | | | | | | | -24%; -49% | | | | | | | | | -20%; -41% | | | | | | | | | -16%; -33% | | | | | | | | | -12%; -25% | | | | | | | | | | -9%; -18% | | | | | | | | | -5%; -10% | | | | | | | | | -1%; -1% | | | | | | | | | 3%; 6% | | | | | | | | 7%; 14% | | | | | | | | 11%; 23% | | | | | | | | | 15%; 30% | | | | | | | | | | 19%; 39% | | | | | | | | | | 22%; 45% | | | | | | | | | 25%; 50% | | | | | | | | | 27%; 56% | | | | | | | | 31%; 64% | | | | | | | | 35%; 71% | | | | | | | | 40%; 81% | | | | | | | |  |  |
| NS | | | | | | | | | -32%; -64% | | | | | | | | | -28%; -57% | | | | | | | | | -25%; -50% | | | | | | | | | -21%; -42% | | | | | | | | | -17%; -35% | | | | | | | | | | -14%; -27% | | | | | | | | | -10%; -20% | | | | | | | | | -7%; -13% | | | | | | | | | -3%; -6% | | | | | | | | 1%; 2% | | | | | | | | 5%; 9% | | | | | | | | | 8%; 16% | | | | | | | | | | 12%; 23% | | | | | | | | | | 15%; 30% | | | | | | | | | 18%; 36% | | | | | | | | | 21%; 43% | | | | | | | | 25%; 50% | | | | | | | | 28%; 56% | | | | | | | | 32%; 65% | | | | | | | |  |  |
| ON | | | | | | | | | -32%; -64% | | | | | | | | | -28%; -57% | | | | | | | | | -25%; -50% | | | | | | | | | -21%; -43% | | | | | | | | | -18%; -35% | | | | | | | | | | -14%; -28% | | | | | | | | | -10%; -21% | | | | | | | | | -7%; -14% | | | | | | | | | -3%; -6% | | | | | | | | 0%; 1% | | | | | | | | 4%; 7% | | | | | | | | | 7%; 14% | | | | | | | | | | 10%; 21% | | | | | | | | | | 13%; 27% | | | | | | | | | 15%; 31% | | | | | | | | | 18%; 37% | | | | | | | | 22%; 44% | | | | | | | | 25%; 51% | | | | | | | | 30%; 60% | | | | | | | |  |  |
| PEI | | | | | | | | | -29%; -58% | | | | | | | | | -25%; -51% | | | | | | | | | -21%; -43% | | | | | | | | | -18%; -35% | | | | | | | | | -14%; -29% | | | | | | | | | | -11%; -22% | | | | | | | | | -7%; -14% | | | | | | | | | -3%; -7% | | | | | | | | | 0%; 1% | | | | | | | | 3%; 7% | | | | | | | | 7%; 15% | | | | | | | | | 11%; 22% | | | | | | | | | | 14%; 28% | | | | | | | | | | 17%; 35% | | | | | | | | | 19%; 39% | | | | | | | | | 22%; 45% | | | | | | | | 26%; 53% | | | | | | | | 29%; 59% | | | | | | | | 34%; 68% | | | | | | | |  |  |
| QC | | | | | | | | | -32%; -64% | | | | | | | | | -28%; -57% | | | | | | | | | -25%; -50% | | | | | | | | | -21%; -42% | | | | | | | | | -17%; -35% | | | | | | | | | | -14%; -28% | | | | | | | | | -10%; -20% | | | | | | | | | -7%; -13% | | | | | | | | | -3%; -6% | | | | | | | | 1%; 1% | | | | | | | | 4%; 8% | | | | | | | | | 7%; 15% | | | | | | | | | | 11%; 21% | | | | | | | | | | 14%; 27% | | | | | | | | | 16%; 32% | | | | | | | | | 18%; 37% | | | | | | | | 22%; 44% | | | | | | | | 25%; 51% | | | | | | | | 30%; 60% | | | | | | | |  |  |
| SK | | | | | | | | | -31%; -64% | | | | | | | | | -28%; -56% | | | | | | | | | -24%; -49% | | | | | | | | | -21%; -42% | | | | | | | | | -17%; -34% | | | | | | | | | | -13%; -27% | | | | | | | | | -10%; -20% | | | | | | | | | -6%; -13% | | | | | | | | | -2%; -5% | | | | | | | | 1%; 2% | | | | | | | | 5%; 10% | | | | | | | | | 8%; 17% | | | | | | | | | | 12%; 24% | | | | | | | | | | 16%; 31% | | | | | | | | | 18%; 37% | | | | | | | | | 22%; 44% | | | | | | | | 25%; 50% | | | | | | | | 28%; 56% | | | | | | | | 32%; 65% | | | | | | | |  |  |
| **Fever and abdominal pain (Reference 6.3%)** | | | | | | | | | | | | | | | | | | | | | | | | | | | | | | | | | | | | | | | | | | | | | | | | | | | | | | | | | | | | | | | | | | | | | | | | | | | | | | | | | | | | | | | | | | | | | | | | | | | | | | | | | | | | | | | | | | | | | | | | | | | | | | | | | | | | | | | | | | | | | | | | | | | | | | | | | | | | | | | | | | | | | | | | | | | | | | | |  |  |  |
|  | | | | | | | | 5% | | | | | | | | | 10% | | | | | | | | | 15% | | | | | | | | | 20% | | | | | | | | | 25% | | | | | | | | | | 30% | | | | | | | | 35% | | | | | | | | | 40% | | | | | | | | 45% | | | | | | | | | 50% | | | | | | | | | 55% | | | | | | | | | 60% | | | | | | | | | 65% | | | | | | | | | | 70% | | | | | | | | | 75% | | | | | | | | | | 80% | | | | | | | | 85% | | | | | | | | 90% | | | | | | | | 95% | | | | | | | |  |  |  |
| BC | | | | | | | | 1%; 1% | | | | | | | | | 1%; 1% | | | | | | | | | 1%; 1% | | | | | | | | | 1%; 2% | | | | | | | | | 1%; 2% | | | | | | | | | | 1%; 2% | | | | | | | | 1%; 2% | | | | | | | | | 1%; 3% | | | | | | | | 1%; 3% | | | | | | | | | 1%; 3% | | | | | | | | | 2%; 3% | | | | | | | | | 2%; 4% | | | | | | | | | 2%; 4% | | | | | | | | | | 2%; 3% | | | | | | | | | 2%; 4% | | | | | | | | | | 3%; 6% | | | | | | | | 3%; 5% | | | | | | | | 3%; 6% | | | | | | | | 3%; 5% | | | | | | | |  |  |  |
| MB | | | | | | | | 0%; 0% | | | | | | | | | 0%; 0% | | | | | | | | | 0%; 0% | | | | | | | | | 0%; 0% | | | | | | | | | 0%; 0% | | | | | | | | | | 0%; 1% | | | | | | | | 1%; 1% | | | | | | | | | 1%; 1% | | | | | | | | 1%; 2% | | | | | | | | | 1%; 2% | | | | | | | | | 1%; 2% | | | | | | | | | 1%; 2% | | | | | | | | | 1%; 2% | | | | | | | | | | 0%; 1% | | | | | | | | | 1%; 1% | | | | | | | | | | 2%; 3% | | | | | | | | 2%; 5% | | | | | | | | 3%; 6% | | | | | | | | 3%; 5% | | | | | | | |  |  |  |
| NL | | | | | | | | 7%; 13% | | | | | | | | | 7%; 13% | | | | | | | | | 7%; 13% | | | | | | | | | 7%; 14% | | | | | | | | | 7%; 14% | | | | | | | | | | 7%; 14% | | | | | | | | 7%; 15% | | | | | | | | | 7%; 15% | | | | | | | | 7%; 15% | | | | | | | | | 7%; 14% | | | | | | | | | 7%; 14% | | | | | | | | | 7%; 14% | | | | | | | | | 7%; 13% | | | | | | | | | | 6%; 13% | | | | | | | | | 6%; 12% | | | | | | | | | | 7%; 15% | | | | | | | | 9%; 18% | | | | | | | | 9%; 18% | | | | | | | | 9%; 18% | | | | | | | |  |  |  |
| NS | | | | | | | | 1%; 1% | | | | | | | | | 0%; 1% | | | | | | | | | 1%; 1% | | | | | | | | | 1%; 1% | | | | | | | | | 1%; 1% | | | | | | | | | | 1%; 2% | | | | | | | | 1%; 2% | | | | | | | | | 1%; 2% | | | | | | | | 1%; 2% | | | | | | | | | 1%; 2% | | | | | | | | | 1%; 2% | | | | | | | | | 1%; 2% | | | | | | | | | 1%; 3% | | | | | | | | | | 1%; 2% | | | | | | | | | 1%; 2% | | | | | | | | | | 2%; 4% | | | | | | | | 3%; 5% | | | | | | | | 3%; 6% | | | | | | | | 3%; 5% | | | | | | | |  |  |  |
| ON | | | | | | | | 0%; -1% | | | | | | | | | 0%; 0% | | | | | | | | | 0%; 0% | | | | | | | | | 0%; 0% | | | | | | | | | 0%; 0% | | | | | | | | | | 0%; 0% | | | | | | | | 0%; 1% | | | | | | | | | 1%; 1% | | | | | | | | 1%; 1% | | | | | | | | | 1%; 1% | | | | | | | | | 1%; 1% | | | | | | | | | 1%; 2% | | | | | | | | | 1%; 2% | | | | | | | | | | 0%; 1% | | | | | | | | | 0%; 1% | | | | | | | | | | 1%; 3% | | | | | | | | 2%; 5% | | | | | | | | 3%; 5% | | | | | | | | 2%; 5% | | | | | | | |  |  |  |
| PEI | | | | | | | | 3%; 7% | | | | | | | | | 3%; 6% | | | | | | | | | 3%; 6% | | | | | | | | | 3%; 6% | | | | | | | | | 3%; 7% | | | | | | | | | | 3%; 6% | | | | | | | | 3%; 6% | | | | | | | | | 3%; 7% | | | | | | | | 3%; 6% | | | | | | | | | 3%; 6% | | | | | | | | | 3%; 6% | | | | | | | | | 3%; 5% | | | | | | | | | 2%; 5% | | | | | | | | | | 3%; 6% | | | | | | | | | 3%; 7% | | | | | | | | | | 3%; 5% | | | | | | | | 4%; 8% | | | | | | | | 5%; 9% | | | | | | | | 5%; 9% | | | | | | | |  |  |  |
| QC | | | | | | | | 0%; 0% | | | | | | | | | 0%; 0% | | | | | | | | | 0%; 0% | | | | | | | | | 0%; 0% | | | | | | | | | 0%; 1% | | | | | | | | | | 0%; 1% | | | | | | | | 1%; 1% | | | | | | | | | 1%; 1% | | | | | | | | 1%; 2% | | | | | | | | | 1%; 2% | | | | | | | | | 1%; 2% | | | | | | | | | 1%; 2% | | | | | | | | | 1%; 2% | | | | | | | | | | 1%; 1% | | | | | | | | | 1%; 1% | | | | | | | | | | 2%; 3% | | | | | | | | 2%; 5% | | | | | | | | 3%; 6% | | | | | | | | 3%; 5% | | | | | | | |  |  |  |
| SK | | | | | | | | 1%; 2% | | | | | | | | | 1%; 2% | | | | | | | | | 1%; 1% | | | | | | | | | 1%; 2% | | | | | | | | | 1%; 1% | | | | | | | | | | 1%; 2% | | | | | | | | 1%; 2% | | | | | | | | | 1%; 3% | | | | | | | | 1%; 3% | | | | | | | | | 1%; 3% | | | | | | | | | 2%; 3% | | | | | | | | | 2%; 3% | | | | | | | | | 2%; 4% | | | | | | | | | | 2%; 3% | | | | | | | | | 2%; 4% | | | | | | | | | | 3%; 6% | | | | | | | | 3%; 5% | | | | | | | | 3%; 6% | | | | | | | | 3%; 5% | | | | | | | |  |  |  |
| **Diarrhea (Reference 84.0%)** | | | | | | | | | | | | | | | | | | | | | | | | | | | | | | | | | | | | | | | | | | | | | | | | | | | | | | | | | | | | | | | | | | | | | | | | | | | | | | | | | | | | | | | | | | | | | | | | | | | | | | | | | | | | | | | | | | | | | | | | | | | | | | | | | | | | | | | | | | | | | | | | | | | | | | | | | | | | | | | | | | | | | | | | | | | | | | |  |  |  |  |
|  |  | | | | | | 5% | | | | | | | | | 10% | | | | | | | | | 15% | | | | | | | | | 20% | | | | | | | | | 25% | | | | | | | | | | 30% | | | | | | | | | | 35% | | | | | | | | | 40% | | | | | | | | | 45% | | | | | | | | | | 50% | | | | | | | | | 55% | | | | | | | | | 60% | | | | | | | | | 65% | | | | | | | | 70% | | | | | | | | | 75% | | | | | | | | 80% | | | | | | | | 85% | | | | | | | | 90% | | | | | | | | 95% | | | | | | | | | | | |
|  | BC | | | | | | -41%; -83% | | | | | | | | | -38%; -77% | | | | | | | | | -35%; -70% | | | | | | | | | -31%; -63% | | | | | | | | | -27%; -56% | | | | | | | | | | -24%; -48% | | | | | | | | | | -20%; -41% | | | | | | | | | -17%; -35% | | | | | | | | | -15%; -29% | | | | | | | | | | -12%; -24% | | | | | | | | | -10%; -20% | | | | | | | | | -8%; -16% | | | | | | | | | -6%; -13% | | | | | | | | -5%; -9% | | | | | | | | | -3%; -6% | | | | | | | | -2%; -3% | | | | | | | | 1%; 2% | | | | | | | | 4%; 8% | | | | | | | | 7%; 14% | | | | | | | | | | | |
|  | MB | | | | | | -42%; -84% | | | | | | | | | -39%; -78% | | | | | | | | | -35%; -71% | | | | | | | | | -32%; -64% | | | | | | | | | -28%; -57% | | | | | | | | | | -24%; -49% | | | | | | | | | | -21%; -42% | | | | | | | | | -18%; -36% | | | | | | | | | -15%; -30% | | | | | | | | | | -13%; -25% | | | | | | | | | -10%; -21% | | | | | | | | | -8%; -17% | | | | | | | | | -7%; -14% | | | | | | | | -5%; -11% | | | | | | | | | -4%; -8% | | | | | | | | -2%; -4% | | | | | | | | 1%; 1% | | | | | | | | 4%; 7% | | | | | | | | 7%; 14% | | | | | | | | | | | |
|  | NL | | | | | | -41%; -83% | | | | | | | | | -38%; -76% | | | | | | | | | -34%; -68% | | | | | | | | | -29%; -59% | | | | | | | | | -25%; -51% | | | | | | | | | | -21%; -42% | | | | | | | | | | -17%; -34% | | | | | | | | | -13%; -27% | | | | | | | | | -10%; -20% | | | | | | | | | | -7%; -15% | | | | | | | | | -5%; -10% | | | | | | | | | -3%; -6% | | | | | | | | | 0%; 0% | | | | | | | | 1%; 2% | | | | | | | | | 3%; 5% | | | | | | | | 4%; 9% | | | | | | | | 7%; 15% | | | | | | | | 11%; 21% | | | | | | | | 14%; 28% | | | | | | | | | | | |
|  | NS | | | | | | -41%; -83% | | | | | | | | | -38%; -77% | | | | | | | | | -35%; -70% | | | | | | | | | -31%; -63% | | | | | | | | | -28%; -56% | | | | | | | | | | -24%; -48% | | | | | | | | | | -21%; -42% | | | | | | | | | -17%; -35% | | | | | | | | | -15%; -30% | | | | | | | | | | -12%; -25% | | | | | | | | | -10%; -20% | | | | | | | | | -8%; -17% | | | | | | | | | -6%; -13% | | | | | | | | -5%; -10% | | | | | | | | | -3%; -6% | | | | | | | | -2%; -4% | | | | | | | | 1%; 2% | | | | | | | | 4%; 8% | | | | | | | | 7%; 14% | | | | | | | | | | | |
|  | ON | | | | | | -42%; -84% | | | | | | | | | -39%; -78% | | | | | | | | | -35%; -71% | | | | | | | | | -32%; -64% | | | | | | | | | -28%; -57% | | | | | | | | | | -24%; -49% | | | | | | | | | | -21%; -43% | | | | | | | | | -18%; -36% | | | | | | | | | -15%; -30% | | | | | | | | | | -13%; -26% | | | | | | | | | -11%; -21% | | | | | | | | | -9%; -17% | | | | | | | | | -7%; -14% | | | | | | | | -5%; -11% | | | | | | | | | -4%; -8% | | | | | | | | -2%; -5% | | | | | | | | 0%; 1% | | | | | | | | 3%; 7% | | | | | | | | 6%; 13% | | | | | | | | | | | |
|  | PEI | | | | | | -45%; -91% | | | | | | | | | -41%; -83% | | | | | | | | | -36%; -73% | | | | | | | | | -32%; -66% | | | | | | | | | -28%; -57% | | | | | | | | | | -24%; -49% | | | | | | | | | | -20%; -41% | | | | | | | | | -17%; -34% | | | | | | | | | -14%; -29% | | | | | | | | | | -11%; -23% | | | | | | | | | -9%; -18% | | | | | | | | | -7%; -13% | | | | | | | | | -4%; -9% | | | | | | | | -3%; -6% | | | | | | | | | -1%; -2% | | | | | | | | 1%; 2% | | | | | | | | 4%; 8% | | | | | | | | 8%; 16% | | | | | | | | 11%; 23% | | | | | | | | | | | |
|  | QC | | | | | | -42%; -84% | | | | | | | | | -39%; -78% | | | | | | | | | -35%; -71% | | | | | | | | | -32%; -64% | | | | | | | | | -28%; -57% | | | | | | | | | | -24%; -49% | | | | | | | | | | -21%; -42% | | | | | | | | | -18%; -36% | | | | | | | | | -15%; -30% | | | | | | | | | | -13%; -25% | | | | | | | | | -10%; -21% | | | | | | | | | -8%; -17% | | | | | | | | | -7%; -13% | | | | | | | | -5%; -11% | | | | | | | | | -4%; -8% | | | | | | | | -2%; -4% | | | | | | | | 1%; 1% | | | | | | | | 4%; 7% | | | | | | | | 7%; 14% | | | | | | | | | | | |
|  | SK | | | | | | -41%; -83% | | | | | | | | | -38%; -77% | | | | | | | | | -35%; -70% | | | | | | | | | -31%; -63% | | | | | | | | | -28%; -56% | | | | | | | | | | -24%; -48% | | | | | | | | | | -20%; -41% | | | | | | | | | -17%; -35% | | | | | | | | | -14%; -29% | | | | | | | | | | -12%; -25% | | | | | | | | | -10%; -20% | | | | | | | | | -8%; -16% | | | | | | | | | -6%; -13% | | | | | | | | -5%; -9% | | | | | | | | | -3%; -6% | | | | | | | | -2%; -3% | | | | | | | | 1%; 3% | | | | | | | | 4%; 8% | | | | | | | | 7%; 14% | | | | | | | | | | | |
|  | **One year old and more (Reference 98.5%)** | | | | | | | | | | | | | | | | | | | | | | | | | | | | | | | | | | | | | | | | | | | | | | | | | | | | | | | | | | | | | | | | | | | | | | | | | | | | | | | | | | | | | | | | | | | | | | | | | | | | | | | | | | | | | | | | | | | | | | | | | | | | | | | | | | | | | | | | | | | | | | | | | | | | | | | | | | | | | | | | | | | | | | | | | | | | |  |  |  |  |  |
|  |  | | | | | 5% | | | | | | | | | 10% | | | | | | | | | 15% | | | | | | | | | 20% | | | | | | | | | 25% | | | | | | | | | | 30% | | | | | | | | | 35% | | | | | | | | | 40% | | | | | | | | | | 45% | | | | | | | | | 50% | | | | | | | | | | 55% | | | | | | | | | 60% | | | | | | | | 65% | | | | | | | | 70% | | | | | | | | | 75% | | | | | | | | | 80% | | | | | | | | 85% | | | | | | | | 90% | | | | | | | | 95% | | | | | | | |  |  |  |  |  |
|  | BC | | | | | -45%; -91% | | | | | | | | | -41%; -83% | | | | | | | | | -37%; -74% | | | | | | | | | -32%; -66% | | | | | | | | | -28%; -57% | | | | | | | | | | -24%; -49% | | | | | | | | | -21%; -42% | | | | | | | | | -17%; -34% | | | | | | | | | | -14%; -28% | | | | | | | | | -11%; -22% | | | | | | | | | | -9%;  -17% | | | | | | | | | -7%;  -14% | | | | | | | | -5%;  -11% | | | | | | | | -4%;  -8% | | | | | | | | | -3%;  -6% | | | | | | | | | -2%;  -4% | | | | | | | | -1%;  -2% | | | | | | | | 0%;  -1% | | | | | | | | 0%; 1% | | | | | | | |  |  |  |  |  |
|  | MB | | | | | -45%; -91% | | | | | | | | | -41%; -83% | | | | | | | | | -37%; -75% | | | | | | | | | -33%; -67% | | | | | | | | | -29%; -58% | | | | | | | | | | -25%; -50% | | | | | | | | | -21%; -42% | | | | | | | | | -17%; -35% | | | | | | | | | | -14%; -28% | | | | | | | | | -11%; -23% | | | | | | | | | | -9%;  -18% | | | | | | | | | -7%;  -15% | | | | | | | | -6%;  -11% | | | | | | | | -4%;  -9% | | | | | | | | | -3%;  -6% | | | | | | | | | -2%;  -5% | | | | | | | | -2%;  -3% | | | | | | | | -1%;  -2% | | | | | | | | 0%;  -1% | | | | | | | |  |  |  |  |  |
|  | NL | | | | | -45%; -91% | | | | | | | | | -41%; -83% | | | | | | | | | -37%; -74% | | | | | | | | | -32%; -65% | | | | | | | | | -28%; -57% | | | | | | | | | | -23%; -47% | | | | | | | | | -20%; -40% | | | | | | | | | -15%; -31% | | | | | | | | | | -12%; -23% | | | | | | | | | -8%;  -16% | | | | | | | | | | -6%;  -12% | | | | | | | | | -4%;  -8% | | | | | | | | -1%;  -3% | | | | | | | | 0%; 0% | | | | | | | | | 2%; 3% | | | | | | | | | 3%; 6% | | | | | | | | 4%; 8% | | | | | | | | 5%; 10% | | | | | | | | 6%; 12% | | | | | | | |  |  |  |  |  |
|  | NS | | | | | -45%; -91% | | | | | | | | | -41%; -83% | | | | | | | | | -37%; -74% | | | | | | | | | -32%; -66% | | | | | | | | | -28%; -57% | | | | | | | | | | -24%; -49% | | | | | | | | | -21%; -42% | | | | | | | | | -17%; -34% | | | | | | | | | | -14%; -28% | | | | | | | | | -11%; -22% | | | | | | | | | | -9%;  -18% | | | | | | | | | -7%;  -14% | | | | | | | | -6%;  -11% | | | | | | | | -4%;  -8% | | | | | | | | | -3%;  -6% | | | | | | | | | -2%;  -4% | | | | | | | | -1%;  -2% | | | | | | | | -1%;  -1% | | | | | | | | 0%; 0% | | | | | | | |  |  |  |  |  |
|  | ON | | | | | -45%; -91% | | | | | | | | | -41%; -84% | | | | | | | | | -37%; -75% | | | | | | | | | -33%; -67% | | | | | | | | | -29%; -58% | | | | | | | | | | -25%; -51% | | | | | | | | | -21%; -43% | | | | | | | | | -17%; -35% | | | | | | | | | | -14%; -29% | | | | | | | | | -11%; -23% | | | | | | | | | | -9%;  -18% | | | | | | | | | -7%;  -15% | | | | | | | | -6%;  -12% | | | | | | | | -5%;  -9% | | | | | | | | | -3%;  -7% | | | | | | | | | -3%;  -5% | | | | | | | | -2%;  -3% | | | | | | | | -1%;  -2% | | | | | | | | -1%;  -1% | | | | | | | |  |  |  |  |  |
|  | PEI | | | | | 3%; 5% | | | | | | | | | 3%; 6% | | | | | | | | | 3%; 7% | | | | | | | | | 3%; 7% | | | | | | | | | 3%; 7% | | | | | | | | | | 4%; 8% | | | | | | | | | 4%; 8% | | | | | | | | | 4%; 7% | | | | | | | | | | 3%; 6% | | | | | | | | | 3%; 6% | | | | | | | | | | 3%; 6% | | | | | | | | | 3%; 6% | | | | | | | | 3%; 6% | | | | | | | | 3%; 6% | | | | | | | | | 3%; 6% | | | | | | | | | 3%; 7% | | | | | | | | 4%; 7% | | | | | | | | 3%; 7% | | | | | | | | 3%; 7% | | | | | | | |  |  |  |  |  |
|  | QC | | | | | -45%; -91% | | | | | | | | | -41%; -84% | | | | | | | | | -37%; -75% | | | | | | | | | -33%; -67% | | | | | | | | | -29%; -58% | | | | | | | | | | -25%; -50% | | | | | | | | | -21%; -42% | | | | | | | | | -17%; -35% | | | | | | | | | | -14%; -28% | | | | | | | | | -11%; -22% | | | | | | | | | | -9%;  -18% | | | | | | | | | -7%;  -15% | | | | | | | | -6%;  -11% | | | | | | | | -4%;  -9% | | | | | | | | | -3%;  -6% | | | | | | | | | -2%;  -5% | | | | | | | | -2%;  -3% | | | | | | | | -1%;  -2% | | | | | | | | 0%;  -1% | | | | | | | |  |  |  |  |  |
|  | SK | | | | | -45%; -91% | | | | | | | | | -41%; -83% | | | | | | | | | -37%; -74% | | | | | | | | | -32%; -66% | | | | | | | | | -28%; -57% | | | | | | | | | | -24%; -49% | | | | | | | | | -21%; -42% | | | | | | | | | -17%; -34% | | | | | | | | | | -14%; -28% | | | | | | | | | -11%; -22% | | | | | | | | | | -9%;  -17% | | | | | | | | | -7%;  -13% | | | | | | | | -5%;  -11% | | | | | | | | -4%;  -8% | | | | | | | | | -3%;  -6% | | | | | | | | | -2%;  -4% | | | | | | | | -1%;  -2% | | | | | | | | 0%; 0% | | | | | | | | 0%; 1% | | | | | | | |  |  |  |  |  |
|  | **Admission (Reference 77.0%)** | | | | | | | | | | | | | | | | | | | | | | | | | | | | | | | | | | | | | | | | | | | | | | | | | | | | | | | | | | | | | | | | | | | | | | | | | | | | | | | | | | | | | | | | | | | | | | | | | | | | | | | | | | | | | | | | | | | | | | | | | | | | | | | | | | | | | | | | | | | | | | | | | | | | | | | | | | | | | | | | | | | | | | | | | | | |  |  |  |  |  |  |
|  |  | | | | 5% | | | | | | | | | 10% | | | | | | | | | 15% | | | | | | | | | 20% | | | | | | | | | 25% | | | | | | | | | | 30% | | | | | | | | | 35% | | | | | | | | | 40% | | | | | | | | | 45% | | | | | | | | | 50% | | | | | | | | | 55% | | | | | | | | | 60% | | | | | | | | | 65% | | | | | | | | | 70% | | | | | | | | | 75% | | | | | | | | | 80% | | | | | | | | 85% | | | | | | | | 90% | | | | | | | | 95% | | | | | | | |  |  |  |  |  |  |
|  | BC | | | | -32%; -65% | | | | | | | | | -27%; -54% | | | | | | | | | -22%; -45% | | | | | | | | | -18%; -37% | | | | | | | | | -15%; -30% | | | | | | | | | | -12%; -23% | | | | | | | | | -8%; -16% | | | | | | | | | -5%; -10% | | | | | | | | | -3%; -6% | | | | | | | | | -1%; -2% | | | | | | | | | 0%; 1% | | | | | | | | | 1%; 3% | | | | | | | | | 1%; 2% | | | | | | | | | 1%; 2% | | | | | | | | | 1%; 1% | | | | | | | | | 1%; 1% | | | | | | | | 1%; 1% | | | | | | | | 1%; 1% | | | | | | | | 1%; 1% | | | | | | | |  |  |  |  |  |  |
|  | MB | | | | -38%; -77% | | | | | | | | | -34%; -69% | | | | | | | | | -28%; -57% | | | | | | | | | -23%; -46% | | | | | | | | | -18%; -36% | | | | | | | | | | -13%; -27% | | | | | | | | | -9%; -19% | | | | | | | | | -6%; -13% | | | | | | | | | -4%; -8% | | | | | | | | | -2%; -3% | | | | | | | | | 0%; 0% | | | | | | | | | 1%; 2% | | | | | | | | | 1%; 1% | | | | | | | | | 0%; 0% | | | | | | | | | 0%; 0% | | | | | | | | | 0%; 0% | | | | | | | | 0%; 0% | | | | | | | | 0%; 0% | | | | | | | | 0%; 0% | | | | | | | |  |  |  |  |  |  |
|  | NL | | | | -34%; -68% | | | | | | | | | -30%; -60% | | | | | | | | | -24%; -49% | | | | | | | | | -18%; -37% | | | | | | | | | -14%; -28% | | | | | | | | | | -9%; -18% | | | | | | | | | -6%; -11% | | | | | | | | | -2%; -4% | | | | | | | | | 0%; 1% | | | | | | | | | 3%; 6% | | | | | | | | | 5%; 9% | | | | | | | | | 6%; 13% | | | | | | | | | 6%; 13% | | | | | | | | | 6%; 13% | | | | | | | | | 7%; 13% | | | | | | | | | 7%; 13% | | | | | | | | 7%; 14% | | | | | | | | 7%; 14% | | | | | | | | 7%; 14% | | | | | | | |  |  |  |  |  |  |
|  | NS | | | | -41%; -82% | | | | | | | | | -36%; -73% | | | | | | | | | -30%; -61% | | | | | | | | | -25%; -51% | | | | | | | | | -20%; -41% | | | | | | | | | | -16%; -31% | | | | | | | | | -12%; -24% | | | | | | | | | -9%; -17% | | | | | | | | | -6%; -11% | | | | | | | | | -3%; -6% | | | | | | | | | -1%; -2% | | | | | | | | | 1%; 2% | | | | | | | | | 1%; 1% | | | | | | | | | 1%; 1% | | | | | | | | | 0%; 1% | | | | | | | | | 0%; 1% | | | | | | | | 0%; 0% | | | | | | | | 0%; 0% | | | | | | | | 1%; 1% | | | | | | | |  |  |  |  |  |  |
|  | ON | | | | -47%; -94% | | | | | | | | | -43%; -87% | | | | | | | | | -36%; -72% | | | | | | | | | -29%; -59% | | | | | | | | | -23%; -46% | | | | | | | | | | -17%; -35% | | | | | | | | | -13%; -26% | | | | | | | | | -10%; -19% | | | | | | | | | -7%; -13% | | | | | | | | | -4%; -8% | | | | | | | | | -2%; -3% | | | | | | | | | 0%; 1% | | | | | | | | | 0%; 0% | | | | | | | | | 0%; 0% | | | | | | | | | 0%; 0% | | | | | | | | | 0%; -1% | | | | | | | | 0%; -1% | | | | | | | | 0%; -1% | | | | | | | | 0%; 0% | | | | | | | |  |  |  |  |  |  |
|  | PEI | | | | -33%; -67% | | | | | | | | | -27%; -55% | | | | | | | | | -22%; -45% | | | | | | | | | -17%; -35% | | | | | | | | | -13%; -26% | | | | | | | | | | -8%; -16% | | | | | | | | | -5%; -10% | | | | | | | | | -3%; -5% | | | | | | | | | 0%; 0% | | | | | | | | | 1%; 3% | | | | | | | | | 4%; 7% | | | | | | | | | 5%; 10% | | | | | | | | | 4%; 8% | | | | | | | | | 3%; 7% | | | | | | | | | 3%; 7% | | | | | | | | | 3%; 6% | | | | | | | | 3%; 7% | | | | | | | | 3%; 6% | | | | | | | | 3%; 6% | | | | | | | |  |  |  |  |  |  |
|  | QC | | | | -38%; -77% | | | | | | | | | -34%; -69% | | | | | | | | | -28%; -57% | | | | | | | | | -23%; -46% | | | | | | | | | -18%; -36% | | | | | | | | | | -13%; -27% | | | | | | | | | -9%; -19% | | | | | | | | | -6%; -13% | | | | | | | | | -4%; -8% | | | | | | | | | -2%; -3% | | | | | | | | | 0%; -1% | | | | | | | | | 1%; 2% | | | | | | | | | 1%; 1% | | | | | | | | | 0%; 0% | | | | | | | | | 0%; 0% | | | | | | | | | 0%; 0% | | | | | | | | 0%; 0% | | | | | | | | 0%; 0% | | | | | | | | 0%; 0% | | | | | | | |  |  |  |  |  |  |
|  | SK | | | | -32%; -64% | | | | | | | | | -27%; -54% | | | | | | | | | -22%; -45% | | | | | | | | | -18%; -37% | | | | | | | | | -15%; -30% | | | | | | | | | | -11%; -23% | | | | | | | | | -8%; -16% | | | | | | | | | -5%; -11% | | | | | | | | | -3%; -6% | | | | | | | | | -1%; -2% | | | | | | | | | 0%; 1% | | | | | | | | | 1%; 3% | | | | | | | | | 1%; 2% | | | | | | | | | 1%; 2% | | | | | | | | | 1%; 1% | | | | | | | | | 1%; 1% | | | | | | | | 1%; 1% | | | | | | | | 0%; 1% | | | | | | | | 1%; 1% | | | | | | | |  |  |  |  |  |  |
|  | **Four weeks recurrence (Reference 18.4%)** | | | | | | | | | | | | | | | | | | | | | | | | | | | | | | | | | | | | | | | | | | | | | | | | | | | | | | | | | | | | | | | | | | | | | | | | | | | | | | | | | | | | | | | | | | | | | | | | | | | | | | | | | | | | | | | | | | | | | | | | | | | | | | | | | | | | | | | | | | | | | | | | | | | | | | | | | | | | | | | | | | | | | | | | | | |  |  |  |  |  |  |  |
|  |  | | | 5% | | | | | | | | | 10% | | | | | | | | | 15% | | | | | | | | | 20% | | | | | | | | | 25% | | | | | | | | | | 30% | | | | | | | | | 35% | | | | | | | | 40% | | | | | | | | | 45% | | | | | | | | | 50% | | | | | | | | | 55% | | | | | | | | | 60% | | | | | | | | | 65% | | | | | | | | | 70% | | | | | | | | | 75% | | | | | | | | | 80% | | | | | | | | | 85% | | | | | | | | 90% | | | | | | | | 95% | | | | | | | | | | | | | | |
|  | BC | | | 6%; 11% | | | | | | | | | 4%; 8% | | | | | | | | | 2%; 4% | | | | | | | | | 0%; 0% | | | | | | | | | -2%;  -4% | | | | | | | | | | -4%;  -9% | | | | | | | | | -7%;  -14% | | | | | | | | -9%;  -19% | | | | | | | | | -12%; -24% | | | | | | | | | -15%; -29% | | | | | | | | | -17%; -35% | | | | | | | | | -20%; -41% | | | | | | | | | -24%; -48% | | | | | | | | | -27%; -54% | | | | | | | | | -30%; -61% | | | | | | | | | -34%; -68% | | | | | | | | | -37%; -75% | | | | | | | | -41%; -82% | | | | | | | | -45%; -90% | | | | | | | | | | | | | | |
|  | MB | | | 5%; 10% | | | | | | | | | 3%; 6% | | | | | | | | | 1%; 3% | | | | | | | | | -1%; -1% | | | | | | | | | -3%; -5% | | | | | | | | | | -5%; -10% | | | | | | | | | -7%;  -15% | | | | | | | | -10%; -20% | | | | | | | | | -12%; -25% | | | | | | | | | -15%; -30% | | | | | | | | | -18%; -36% | | | | | | | | | -21%; -42% | | | | | | | | | -24%; -48% | | | | | | | | | -27%; -55% | | | | | | | | | -30%; -61% | | | | | | | | | -34%; -69% | | | | | | | | | -37%; -75% | | | | | | | | -41%; -82% | | | | | | | | -45%; -90% | | | | | | | | | | | | | | |
|  | NL | | | 7%; 14% | | | | | | | | | 7%; 14% | | | | | | | | | 7%; 14% | | | | | | | | | 7%; 13% | | | | | | | | | 7%; 14% | | | | | | | | | | 7%; 14% | | | | | | | | | 7%; 13% | | | | | | | | 7%; 14% | | | | | | | | | 7%; 13% | | | | | | | | | 7%; 14% | | | | | | | | | 7%; 13% | | | | | | | | | 6%; 13% | | | | | | | | | 7%; 13% | | | | | | | | | 7%; 14% | | | | | | | | | 7%; 14% | | | | | | | | | 7%; 13% | | | | | | | | | 7%; 13% | | | | | | | | 7%; 13% | | | | | | | | 7%; 14% | | | | | | | | | | | | | | |
|  | NS | | | 5%; 11% | | | | | | | | | 3%; 7% | | | | | | | | | 2%; 3% | | | | | | | | | 0%;  -1% | | | | | | | | | -2%;  -5% | | | | | | | | | | -5%;  -9% | | | | | | | | | -7%;  -14% | | | | | | | | -9%;  -19% | | | | | | | | | -12%; -24% | | | | | | | | | -15%; -29% | | | | | | | | | -18%; -35% | | | | | | | | | -20%; -41% | | | | | | | | | -24%; -48% | | | | | | | | | -27%; -54% | | | | | | | | | -30%; -61% | | | | | | | | | -34%; -68% | | | | | | | | | -37%; -75% | | | | | | | | -41%; -82% | | | | | | | | -45%; -90% | | | | | | | | | | | | | | |
|  | ON | | | 5%; 9% | | | | | | | | | 3%; 6% | | | | | | | | | 1%; 2% | | | | | | | | | -1%;  -2% | | | | | | | | | -3%;  -6% | | | | | | | | | | -5%;  -10% | | | | | | | | | -7%;  -15% | | | | | | | | -10%; -20% | | | | | | | | | -12%; -25% | | | | | | | | | -15%; -31% | | | | | | | | | -18%; -36% | | | | | | | | | -21%; -42% | | | | | | | | | -24%; -48% | | | | | | | | | -27%; -55% | | | | | | | | | -30%; -62% | | | | | | | | | -34%; -68% | | | | | | | | | -37%; -75% | | | | | | | | -41%; -82% | | | | | | | | -45%; -90% | | | | | | | | | | | | | | |
|  | PEI | | | 9%; 19% | | | | | | | | | 7%; 14% | | | | | | | | | 5%; 10% | | | | | | | | | 3%; 6% | | | | | | | | | 0%; 1% | | | | | | | | | | -3%;  -6% | | | | | | | | | -5%;  -11% | | | | | | | | -8%;  -15% | | | | | | | | | -11%; -21% | | | | | | | | | -14%; -28% | | | | | | | | | -16%; -33% | | | | | | | | | -20%; -40% | | | | | | | | | -23%; -47% | | | | | | | | | -26%; -53% | | | | | | | | | -30%; -60% | | | | | | | | | -33%; -68% | | | | | | | | | -37%; -75% | | | | | | | | -40%; -82% | | | | | | | | -44%; -90% | | | | | | | | | | | | | | |
|  | QC | | | 5%; 10% | | | | | | | | | 3%; 6% | | | | | | | | | 1%; 3% | | | | | | | | | -1%;  -1% | | | | | | | | | -3%;  -6% | | | | | | | | | | -5%;  -10% | | | | | | | | | -7%;  -15% | | | | | | | | -10%; -20% | | | | | | | | | -12%; -25% | | | | | | | | | -15%; -30% | | | | | | | | | -18%; -36% | | | | | | | | | -21%; -42% | | | | | | | | | -24%; -48% | | | | | | | | | -27%; -55% | | | | | | | | | -30%; -62% | | | | | | | | | -34%; -68% | | | | | | | | | -37%; -75% | | | | | | | | -41%; -82% | | | | | | | | -45%; -90% | | | | | | | | | | | | | | |
|  | SK | | | 6%; 11% | | | | | | | | | 4%; 8% | | | | | | | | | 2%; 4% | | | | | | | | | 0%; 0% | | | | | | | | | -2%;  -5% | | | | | | | | | | -5%;  -9% | | | | | | | | | -7%;  -14% | | | | | | | | -9%;  -19% | | | | | | | | | -12%; -24% | | | | | | | | | -15%; -29% | | | | | | | | | -18%; -35% | | | | | | | | | -21%; -42% | | | | | | | | | -24%; -48% | | | | | | | | | -27%; -55% | | | | | | | | | -30%; -61% | | | | | | | | | -34%; -68% | | | | | | | | | -37%; -75% | | | | | | | | -41%; -82% | | | | | | | | -45%; -90% | | | | | | | | | | | | | | |
|  | **Hospitalisation within four weeks (Reference 18.5%)** | | | | | | | | | | | | | | | | | | | | | | | | | | | | | | | | | | | | | | | | | | | | | | | | | | | | | | | | | | | | | | | | | | | | | | | | | | | | | | | | | | | | | | | | | | | | | | | | | | | | | | | | | | | | | | | | | | | | | | | | | | | | | | | | | | | | | | | | | | | | | | | | | | | | | | | | | | | | | | | | | | | | | | | | | | | | | | | | | |
|  |  | | 5% | | | | | | | | | 10% | | | | | | | | 15% | | | | | | | | | 20% | | | | | | | | | 25% | | | | | | | | | 30% | | | | | | | | | | 35% | | | | | | | | | 40% | | | | | | | | | 45% | | | | | | | | | 50% | | | | | | | | | 55% | | | | | | | | | 60% | | | | | | | | | 65% | | | | | | | | | 70% | | | | | | | | | 75% | | | | | | | | | 80% | | | | | | | | | 85% | | | | | | | | | 90% | | | | | | | | 95% | | | | | | | | | | | | | | |
|  | BC | | -3%; -6% | | | | | | | | | -2%;  -3% | | | | | | | | 0%;  -1% | | | | | | | | | 1%; 2% | | | | | | | | | 3%; 5% | | | | | | | | | 4%; 8% | | | | | | | | | | 6%; 12% | | | | | | | | | 8%; 15% | | | | | | | | | 9%; 19% | | | | | | | | | 11%; 23% | | | | | | | | | 13%; 26% | | | | | | | | | 15%; 30% | | | | | | | | | 17%; 34% | | | | | | | | | 19%; 38% | | | | | | | | | 21%; 42% | | | | | | | | | 22%; 45% | | | | | | | | | 24%; 49% | | | | | | | | | 27%; 55% | | | | | | | | 30%; 60% | | | | | | | | | | | | | | |
|  | MB | | -4%; -8% | | | | | | | | | -2%;  -5% | | | | | | | | -1%;  -2% | | | | | | | | | 0%; 1% | | | | | | | | | 2%; 4% | | | | | | | | | 4%; 7% | | | | | | | | | | 5%; 11% | | | | | | | | | 7%; 14% | | | | | | | | | 9%; 17% | | | | | | | | | 10%; 21% | | | | | | | | | 12%; 25% | | | | | | | | | 14%; 28% | | | | | | | | | 16%; 32% | | | | | | | | | 18%; 37% | | | | | | | | | 20%; 41% | | | | | | | | | 21%; 42% | | | | | | | | | 22%; 44% | | | | | | | | | 22%; 44% | | | | | | | | 21%; 43% | | | | | | | | | | | | | | |
|  | NL | | 1%; 3% | | | | | | | | | 3%; 6% | | | | | | | | 5%; 11% | | | | | | | | | 7%; 15% | | | | | | | | | 9%; 19% | | | | | | | | | 11%; 23% | | | | | | | | | | 13%; 27% | | | | | | | | | 16%; 32% | | | | | | | | | 18%; 37% | | | | | | | | | 20%; 41% | | | | | | | | | 23%; 46% | | | | | | | | | 25%; 50% | | | | | | | | | 27%; 55% | | | | | | | | | 30%; 60% | | | | | | | | | 32%; 64% | | | | | | | | | 33%; 67% | | | | | | | | | 33%; 67% | | | | | | | | | 33%; 68% | | | | | | | | 33%; 67% | | | | | | | | | | | | | | |
|  | NS | | -3%; -6% | | | | | | | | | -2%;  -4% | | | | | | | | -1%;  -1% | | | | | | | | | 1%; 1% | | | | | | | | | 2%; 5% | | | | | | | | | 4%; 8% | | | | | | | | | | 5%; 10% | | | | | | | | | 7%; 14% | | | | | | | | | 8%; 17% | | | | | | | | | 10%; 20% | | | | | | | | | 12%; 24% | | | | | | | | | 13%; 27% | | | | | | | | | 15%; 30% | | | | | | | | | 16%; 33% | | | | | | | | | 17%; 34% | | | | | | | | | 17%; 34% | | | | | | | | | 17%; 35% | | | | | | | | | 18%; 37% | | | | | | | | 18%; 36% | | | | | | | | | | | | | | |
|  | ON | | -4%; -8% | | | | | | | | | -3%;  -5% | | | | | | | | -1%;  -2% | | | | | | | | | 0%; 0% | | | | | | | | | 2%; 3% | | | | | | | | | 3%; 6% | | | | | | | | | | 5%; 9% | | | | | | | | | 6%; 12% | | | | | | | | | 8%; 16% | | | | | | | | | 9%; 19% | | | | | | | | | 11%; 22% | | | | | | | | | 12%; 25% | | | | | | | | | 14%; 28% | | | | | | | | | 15%; 31% | | | | | | | | | 16%; 32% | | | | | | | | | 16%; 33% | | | | | | | | | 17%; 34% | | | | | | | | | 17%; 34% | | | | | | | | 16%; 33% | | | | | | | | | | | | | | |
|  | PEI | | 1%; 1% | | | | | | | | | 2%; 3% | | | | | | | | 2%; 5% | | | | | | | | | 4%; 8% | | | | | | | | | 5%; 10% | | | | | | | | | 7%; 13% | | | | | | | | | | 8%; 15% | | | | | | | | | 9%; 19% | | | | | | | | | 11%; 22% | | | | | | | | | 13%; 26% | | | | | | | | | 14%; 29% | | | | | | | | | 16%; 32% | | | | | | | | | 18%; 36% | | | | | | | | | 20%; 40% | | | | | | | | | 21%; 43% | | | | | | | | | 22%; 45% | | | | | | | | | 22%; 45% | | | | | | | | | 23%; 46% | | | | | | | | 22%; 44% | | | | | | | | | | | | | | |
|  | QC | | -4%; -7% | | | | | | | | | -2%;  -5% | | | | | | | | -1%;  -2% | | | | | | | | | 0%; 1% | | | | | | | | | 2%; 4% | | | | | | | | | 4%; 7% | | | | | | | | | | 5%; 10% | | | | | | | | | 7%; 14% | | | | | | | | | 9%; 17% | | | | | | | | | 10%; 21% | | | | | | | | | 12%; 25% | | | | | | | | | 14%; 28% | | | | | | | | | 16%; 32% | | | | | | | | | 18%; 37% | | | | | | | | | 20%; 41% | | | | | | | | | 21%; 42% | | | | | | | | | 21%; 43% | | | | | | | | | 22%; 44% | | | | | | | | 21%; 43% | | | | | | | | | | | | | | |
|  | SK | | -3%; -6% | | | | | | | | | -2%;  -3% | | | | | | | | 0%;  -1% | | | | | | | | | 1%; 2% | | | | | | | | | 3%; 5% | | | | | | | | | 4%; 8% | | | | | | | | | | 6%; 12% | | | | | | | | | 8%; 16% | | | | | | | | | 9%; 19% | | | | | | | | | 11%; 23% | | | | | | | | | 13%; 26% | | | | | | | | | 15%; 30% | | | | | | | | | 17%; 34% | | | | | | | | | 19%; 38% | | | | | | | | | 21%; 42% | | | | | | | | | 22%; 45% | | | | | | | | | 24%; 49% | | | | | | | | | 27%; 55% | | | | | | | | 30%; 60% | | | | | | | | | | | | | | |
|  | **Information on diarrhea available (Reference 83%)** | | | | | | | | | | | | | | | | | | | | | | | | | | | | | | | | | | | | | | | | | | | | | | | | | | | | | | | | | | | | | | | | | | | | | | | | | | | | | | | | | | | | | | | | | | | | | | | | | | | | | | | | | | | | | | | | | | | | | | | | | | | | | | | | | | | | | | | | | | | | | | | | | | | | | | | | | | | | | | | | | | | | | | | | | |  |  |  |  |  |  |  |  |
|  |  | 5% | | | | | | | | | 10% | | | | | | | | | | 15% | | | | | | | | | 20% | | | | | | | | | 25% | | | | | | | | | 30% | | | | | | | | | | 35% | | | | | | | | | | 40% | | | | | | | | | 45% | | | | | | | | | 50% | | | | | | | | | 55% | | | | | | | | | 60% | | | | | | | | | 65% | | | | | | | | | 70% | | | | | | | | | 75% | | | | | | | | | 80% | | | | | | | 85% | | | | | | | | 90% | | | | | | | | 95% | | | | | | | |  |  |  |  |  |  |  |  |
|  | BC | -38%; -76% | | | | | | | | | -31%; -63% | | | | | | | | | | -16%; -33% | | | | | | | | | -7%;  -14% | | | | | | | | | -2%;  -3% | | | | | | | | | 3%; 6% | | | | | | | | | | 7%; 14% | | | | | | | | | | 9%; 19% | | | | | | | | | 11%; 23% | | | | | | | | | 13%; 27% | | | | | | | | | 17%; 33% | | | | | | | | | 16%; 32% | | | | | | | | | 13%; 25% | | | | | | | | | 9%; 18% | | | | | | | | | 5%; 11% | | | | | | | | | 2%; 5% | | | | | | | 0%;  -1% | | | | | | | | -1%;  -2% | | | | | | | | -1%;  -3% | | | | | | | |  |  |  |  |  |  |  |  |
|  | MB | -39%; -79% | | | | | | | | | -31%; -63% | | | | | | | | | | -16%; -33% | | | | | | | | | -7%;  -14% | | | | | | | | | -2%;  -3% | | | | | | | | | 3%; 6% | | | | | | | | | | 7%; 14% | | | | | | | | | | 9%; 19% | | | | | | | | | 11%; 23% | | | | | | | | | 13%; 27% | | | | | | | | | 15%; 30% | | | | | | | | | 13%; 27% | | | | | | | | | 10%; 21% | | | | | | | | | 7%; 14% | | | | | | | | | 4%; 8% | | | | | | | | | 2%; 3% | | | | | | | -1%;  -2% | | | | | | | | -1%;  -3% | | | | | | | | -1%;  -3% | | | | | | | |  |  |  |  |  |  |  |  |
|  | NL | -39%; -79% | | | | | | | | | -31%; -63% | | | | | | | | | | -16%; -33% | | | | | | | | | -6%;  -12% | | | | | | | | | 1%; 1% | | | | | | | | | 6%; 12% | | | | | | | | | | 10%; 21% | | | | | | | | | | 14%; 27% | | | | | | | | | 16%; 33% | | | | | | | | | 19%; 39% | | | | | | | | | 21%; 43% | | | | | | | | | 21%; 42% | | | | | | | | | 17%; 35% | | | | | | | | | 14%; 28% | | | | | | | | | 11%; 23% | | | | | | | | | 8%; 17% | | | | | | | 6%; 12% | | | | | | | | 5%; 10% | | | | | | | | 5%; 11% | | | | | | | |  |  |  |  |  |  |  |  |
|  | NS | -38%; -78% | | | | | | | | | -31%; -63% | | | | | | | | | | -16%; -33% | | | | | | | | | -7%;  -14% | | | | | | | | | -2%;  -3% | | | | | | | | | 3%; 6% | | | | | | | | | | 6%; 13% | | | | | | | | | | 9%; 18% | | | | | | | | | 11%; 22% | | | | | | | | | 13%; 26% | | | | | | | | | 16%; 32% | | | | | | | | | 15%; 31% | | | | | | | | | 12%; 24% | | | | | | | | | 8%; 17% | | | | | | | | | 5%; 10% | | | | | | | | | 2%; 4% | | | | | | | -1%;  -2% | | | | | | | | -2%;  -3% | | | | | | | | -2%;  -3% | | | | | | | |  |  |  |  |  |  |  |  |
|  | ON | -39%; -79% | | | | | | | | | -31%; -63% | | | | | | | | | | -16%; -33% | | | | | | | | | -7%;  -14% | | | | | | | | | -2%;  -4% | | | | | | | | | 3%; 6% | | | | | | | | | | 6%; 13% | | | | | | | | | | 9%; 18% | | | | | | | | | 11%; 22% | | | | | | | | | 13%; 26% | | | | | | | | | 14%; 29% | | | | | | | | | 13%; 26% | | | | | | | | | 10%; 20% | | | | | | | | | 7%; 14% | | | | | | | | | 4%; 8% | | | | | | | | | 1%; 3% | | | | | | | -1%;  -2% | | | | | | | | -2%;  -3% | | | | | | | | -2%;  -3% | | | | | | | |  |  |  |  |  |  |  |  |
|  | PEI | -38%; -77% | | | | | | | | | -20%; -41% | | | | | | | | | | -5%; -11% | | | | | | | | | 5%; 10% | | | | | | | | | 9%; 19% | | | | | | | | | 12%; 24% | | | | | | | | | | 14%; 29% | | | | | | | | | | 16%; 33% | | | | | | | | | 18%; 36% | | | | | | | | | 20%; 40% | | | | | | | | | 20%; 41% | | | | | | | | | 18%; 37% | | | | | | | | | 14%; 29% | | | | | | | | | 11%; 23% | | | | | | | | | 8%; 16% | | | | | | | | | 5%; 10% | | | | | | | 2%; 4% | | | | | | | | 2%; 3% | | | | | | | | 2%; 5% | | | | | | | |  |  |  |  |  |  |  |  |
|  | QC | -39%; -79% | | | | | | | | | -31%; -64% | | | | | | | | | | -17%; -33% | | | | | | | | | -7%;  -14% | | | | | | | | | -2%;  -3% | | | | | | | | | 3%; 7% | | | | | | | | | | 7%; 14% | | | | | | | | | | 9%; 19% | | | | | | | | | 11%; 23% | | | | | | | | | 13%; 27% | | | | | | | | | 15%; 30% | | | | | | | | | 14%; 27% | | | | | | | | | 10%; 21% | | | | | | | | | 7%; 14% | | | | | | | | | 4%; 8% | | | | | | | | | 2%; 3% | | | | | | | -1%;  -2% | | | | | | | | -1%;  -3% | | | | | | | | -1%;  -3% | | | | | | | |  |  |  |  |  |  |  |  |
|  | SK | -38%; -76% | | | | | | | | | -31%; -63% | | | | | | | | | | -17%; -34% | | | | | | | | | -7%;  -15% | | | | | | | | | -2%;  -3% | | | | | | | | | 3%; 6% | | | | | | | | | | 7%; 14% | | | | | | | | | | 9%; 19% | | | | | | | | | 12%; 23% | | | | | | | | | 13%; 27% | | | | | | | | | 16%; 33% | | | | | | | | | 16%; 32% | | | | | | | | | 13%; 25% | | | | | | | | | 7%; 14% | | | | | | | | | 5%; 11% | | | | | | | | | 2%; 4% | | | | | | | -1%;  -1% | | | | | | | | -1%;  -3% | | | | | | | | -1%;  -3% | | | | | | | |  |  |  |  |  |  |  |  |
|  | Absolute difference (province – CNISP); Relative difference (province – CNISP)/CNISP | | | | | | | | | | | | | | | | | | | | | | | | | | | | | | | | | | | | | | | | | | | | | | | |  |  |  |  |  |  |  |  |  |  |  |  |  |  |  |  |  |  |  |  |  |  |  |  |  |  |  |  |  |  |  |  |  |  |  |  |  |  |  |  |  |  |  |  |  |  |  |  |  |  |  |  |  |  |  |  |  |  |  |  |  |  |  |  |  |  |  |  |  |  |  |  |  |  |  |  |  |  |  |  |  |  |  |  |  |  |  |  |  |  |  |  |  |  |  |  |  |  |  |  |  |  |  |  |  |  |  |  |  |  |  |  |  |  |  |  |  |  |  |  |  |  |  |  |  |  |  |  |  |  |
|  | Comparable | | | | | | | | | | | | | | | | | | | | | | | | | | | | | | | | | | | | | | | | | | | | | | | |  |  |  |  |  |  |  |  |  |  |  |  |  |  |  |  |  |  |  |  |  |  |  |  |  |  |  |  |  |  |  |  |  |  |  |  |  |  |  |  |  |  |  |  |  |  |  |  |  |  |  |  |  |  |  |  |  |  |  |  |  |  |  |  |  |  |  |  |  |  |  |  |  |  |  |  |  |  |  |  |  |  |  |  |  |  |  |  |  |  |  |  |  |  |  |  |  |  |  |  |  |  |  |  |  |  |  |  |  |  |  |  |  |  |  |  |  |  |  |  |  |  |  |  |  |  |  |  |  |  |
|  | Province not comparable with CNISP | | | | | | | | | | | | | | | | | | | | | | | | | | | | | | | | | | | | | | | | | | | | | | | |  |  |  |  |  |  |  |  |  |  |  |  |  |  |  |  |  |  |  |  |  |  |  |  |  |  |  |  |  |  |  |  |  |  |  |  |  |  |  |  |  |  |  |  |  |  |  |  |  |  |  |  |  |  |  |  |  |  |  |  |  |  |  |  |  |  |  |  |  |  |  |  |  |  |  |  |  |  |  |  |  |  |  |  |  |  |  |  |  |  |  |  |  |  |  |  |  |  |  |  |  |  |  |  |  |  |  |  |  |  |  |  |  |  |  |  |  |  |  |  |  |  |  |  |  |  |  |  |  |  |

**Table S7-** Distribution of absolute and relative differences of proportions of patients meeting case definition: provinces vs CNISP with both varying proportions of elements, by province CDI

| **Two days of admission (Reference 7.2%)** | | | | | | | | | | | | | | | | | | | | | | | | | | | | | | | | | | | | | | | | | | | | | | | | | | | | | | | | | | | | | | | | | | | | | | | | | | | | | | | | | | | | | | | | | | | | | | | | | | | | | | | | | | | | | | | | | | | | | | | | | | | | | | | | | | | | | | | | | | | | | | | | | | | | | | | | | | | | | | | | | | | | | | | | | | |  |
| --- | --- | --- | --- | --- | --- | --- | --- | --- | --- | --- | --- | --- | --- | --- | --- | --- | --- | --- | --- | --- | --- | --- | --- | --- | --- | --- | --- | --- | --- | --- | --- | --- | --- | --- | --- | --- | --- | --- | --- | --- | --- | --- | --- | --- | --- | --- | --- | --- | --- | --- | --- | --- | --- | --- | --- | --- | --- | --- | --- | --- | --- | --- | --- | --- | --- | --- | --- | --- | --- | --- | --- | --- | --- | --- | --- | --- | --- | --- | --- | --- | --- | --- | --- | --- | --- | --- | --- | --- | --- | --- | --- | --- | --- | --- | --- | --- | --- | --- | --- | --- | --- | --- | --- | --- | --- | --- | --- | --- | --- | --- | --- | --- | --- | --- | --- | --- | --- | --- | --- | --- | --- | --- | --- | --- | --- | --- | --- | --- | --- | --- | --- | --- | --- | --- | --- | --- | --- | --- | --- | --- | --- | --- | --- | --- | --- | --- | --- | --- | --- | --- | --- | --- | --- | --- | --- | --- | --- | --- | --- | --- | --- | --- | --- | --- | --- | --- | --- | --- | --- | --- | --- |
|  | | | | | | | | | | 5% | | | | | | | | | 10% | | | | | | | | 15% | | | | | | | | | 20% | | | | | | | | 25% | | | | | | | | | | 30% | | | | | | | | | 35% | | | | | | | | | 40% | | | | | | | | | 45% | | | | | | | | 50% | | | | | | | | 55% | | | | | | | | 60% | | | | | | | | 65% | | | | | | | | | 70% | | | | | | | | 75% | | | | | | | | 80% | | | | | | | | 85% | | | | | | | 90% | | | | | | | | | 95% | | | | | | | | |  |
| BC | | | | | | | | | | 1%; 1% | | | | | | | | | 1%; 1% | | | | | | | | 1%; 1% | | | | | | | | | 1%; 2% | | | | | | | | 1%; 2% | | | | | | | | | | 1%; 3% | | | | | | | | | 2%; 4% | | | | | | | | | 2%; 4% | | | | | | | | | 1%; 3% | | | | | | | | 2%; 4% | | | | | | | | 1%; 3% | | | | | | | | 1%; 3% | | | | | | | | 1%; 2% | | | | | | | | | 1%; 2% | | | | | | | | 1%; 2% | | | | | | | | 1%; 3% | | | | | | | | 1%; 2% | | | | | | | 1%; 2% | | | | | | | | | 1%; 2% | | | | | | | | |  |
| MB | | | | | | | | | | 0%; 0% | | | | | | | | | 0%; 0% | | | | | | | | 0%; 0% | | | | | | | | | 0%; 0% | | | | | | | | 0%; 0% | | | | | | | | | | 0%; 0% | | | | | | | | | 0%; 0% | | | | | | | | | 0%; 0% | | | | | | | | | 0%; 0% | | | | | | | | 0%; 0% | | | | | | | | 0%; 0% | | | | | | | | 0%; 0% | | | | | | | | 0%; 0% | | | | | | | | | 0%; 0% | | | | | | | | 0%; 0% | | | | | | | | 0%; 0% | | | | | | | | 0%; 0% | | | | | | | 0%; 0% | | | | | | | | | 0%; 0% | | | | | | | | |  |
| NL | | | | | | | | | | 6%; 13% | | | | | | | | | 6%; 13% | | | | | | | | 7%; 13% | | | | | | | | | 7%; 14% | | | | | | | | 6%; 13% | | | | | | | | | | 5%; 11% | | | | | | | | | 4%; 9% | | | | | | | | | 3%; 7% | | | | | | | | | 4%; 7% | | | | | | | | 3%; 6% | | | | | | | | 5%; 10% | | | | | | | | 5%; 10% | | | | | | | | 4%; 8% | | | | | | | | | 4%; 7% | | | | | | | | 3%; 6% | | | | | | | | 3%; 7% | | | | | | | | 4%; 8% | | | | | | | 5%; 10% | | | | | | | | | 6%; 12% | | | | | | | | |  |
| NS | | | | | | | | | | 0%; 0% | | | | | | | | | 0%; 1% | | | | | | | | 0%; 0% | | | | | | | | | 0%; 1% | | | | | | | | 1%; 2% | | | | | | | | | | 1%; 3% | | | | | | | | | 2%; 4% | | | | | | | | | 2%; 3% | | | | | | | | | 1%; 3% | | | | | | | | 2%; 4% | | | | | | | | 2%; 3% | | | | | | | | 1%; 3% | | | | | | | | 1%; 2% | | | | | | | | | 1%; 2% | | | | | | | | 1%; 2% | | | | | | | | 1%; 2% | | | | | | | | 1%; 2% | | | | | | | 1%; 2% | | | | | | | | | 1%; 2% | | | | | | | | |  |
| ON | | | | | | | | | | 0%; -1% | | | | | | | | | 0%; -1% | | | | | | | | 0%; 0% | | | | | | | | | 0%; 0% | | | | | | | | 0%; 0% | | | | | | | | | | 0%; 0% | | | | | | | | | 0%; 0% | | | | | | | | | 0%; 0% | | | | | | | | | 0%; 0% | | | | | | | | 0%; 0% | | | | | | | | 0%; 0% | | | | | | | | 0%; 0% | | | | | | | | 0%; 0% | | | | | | | | | 0%; 0% | | | | | | | | 0%; 0% | | | | | | | | 0%; 0% | | | | | | | | 0%; 0% | | | | | | | 0%; 0% | | | | | | | | | 0%; 0% | | | | | | | | |  |
| PEI | | | | | | | | | | 2%; 4% | | | | | | | | | 5%; 10% | | | | | | | | 8%; 15% | | | | | | | | | 9%; 19% | | | | | | | | 13%; 27% | | | | | | | | | | 16%; 32% | | | | | | | | | 18%; 37% | | | | | | | | | 22%; 44% | | | | | | | | | 24%; 48% | | | | | | | | 27%; 54% | | | | | | | | 29%; 59% | | | | | | | | 30%; 61% | | | | | | | | 31%; 62% | | | | | | | | | 34%; 69% | | | | | | | | 37%; 74% | | | | | | | | 38%; 77% | | | | | | | | 39%; 80% | | | | | | | 43%; 86% | | | | | | | | | 49%; 98% | | | | | | | | |  |
| QC | | | | | | | | | | 0%; 0% | | | | | | | | | 0%; 0% | | | | | | | | 0%; 0% | | | | | | | | | 0%; 0% | | | | | | | | 0%; 0% | | | | | | | | | | 0%; 0% | | | | | | | | | 0%; 0% | | | | | | | | | 0%; 0% | | | | | | | | | 0%; 0% | | | | | | | | 0%; 0% | | | | | | | | 0%; 0% | | | | | | | | 0%; 0% | | | | | | | | 0%; 0% | | | | | | | | | 0%; 0% | | | | | | | | 0%; 0% | | | | | | | | 0%; 0% | | | | | | | | 0%; 0% | | | | | | | 0%; 0% | | | | | | | | | 0%; 0% | | | | | | | | |  |
| SK | | | | | | | | | | 1%; 1% | | | | | | | | | 1%; 1% | | | | | | | | 1%; 1% | | | | | | | | | 1%; 2% | | | | | | | | 1%; 2% | | | | | | | | | | 1%; 3% | | | | | | | | | 2%; 4% | | | | | | | | | 2%; 4% | | | | | | | | | 1%; 2% | | | | | | | | 2%; 4% | | | | | | | | 1%; 3% | | | | | | | | 1%; 3% | | | | | | | | 1%; 2% | | | | | | | | | 1%; 2% | | | | | | | | 1%; 2% | | | | | | | | 1%; 3% | | | | | | | | 1%; 3% | | | | | | | 1%; 2% | | | | | | | | | 1%; 2% | | | | | | | | |  |
| **Three days of admission (Reference 49.3%)** | | | | | | | | | | | | | | | | | | | | | | | | | | | | | | | | | | | | | | | | | | | | | | | | | | | | | | | | | | | | | | | | | | | | | | | | | | | | | | | | | | | | | | | | | | | | | | | | | | | | | | | | | | | | | | | | | | | | | | | | | | | | | | | | | | | | | | | | | | | | | | | | | | | | | | | | | | | | | | | | | | | | | | | | | |  |  |
|  | | | | | | | | | 5% | | | | | | | | | 10% | | | | | | | | 15% | | | | | | | | 20% | | | | | | | | | 25% | | | | | | | | | | 30% | | | | | | | | | 35% | | | | | | | | | 40% | | | | | | | | | 45% | | | | | | | | 50% | | | | | | | | 55% | | | | | | | | 60% | | | | | | | | 65% | | | | | | | | 70% | | | | | | | | 75% | | | | | | | | 80% | | | | | | | | | 85% | | | | | | | 90% | | | | | | | | | 95% | | | | | | | | |  |  |
| BC | | | | | | | | | 0%; 1% | | | | | | | | | 0%; 0% | | | | | | | | 0%; 1% | | | | | | | | 0%; 0% | | | | | | | | | 0%; 1% | | | | | | | | | | 0%; 1% | | | | | | | | | 0%; 1% | | | | | | | | | 0%; 1% | | | | | | | | | 1%; 1% | | | | | | | | 1%; 1% | | | | | | | | 1%; 2% | | | | | | | | 1%; 2% | | | | | | | | 1%; 2% | | | | | | | | 2%; 3% | | | | | | | | 2%; 4% | | | | | | | | 3%; 5% | | | | | | | | | 3%; 4% | | | | | | | 3%; 3% | | | | | | | | | 2%; 3% | | | | | | | | |  |  |
| MB | | | | | | | | | 0%; 0% | | | | | | | | | 0%; 0% | | | | | | | | 0%; 0% | | | | | | | | 0%; 0% | | | | | | | | | 0%; 0% | | | | | | | | | | 0%; 0% | | | | | | | | | 0%; 0% | | | | | | | | | 0%; 0% | | | | | | | | | 0%; 0% | | | | | | | | 0%; 0% | | | | | | | | 0%; 0% | | | | | | | | 0%; 0% | | | | | | | | 0%; 0% | | | | | | | | 0%; 0% | | | | | | | | 0%; 0% | | | | | | | | 0%; 0% | | | | | | | | | 0%; 0% | | | | | | | 0%; 0% | | | | | | | | | 0%; 0% | | | | | | | | |  |  |
| NL | | | | | | | | | 4%; 21% | | | | | | | | | 4%; 19% | | | | | | | | 4%; 17% | | | | | | | | 4%; 15% | | | | | | | | | 5%; 15% | | | | | | | | | | 5%; 14% | | | | | | | | | 5%; 14% | | | | | | | | | 6%; 14% | | | | | | | | | 6%; 13% | | | | | | | | 7%; 13% | | | | | | | | 7%; 14% | | | | | | | | 8%; 14% | | | | | | | | 9%; 14% | | | | | | | | 8%; 13% | | | | | | | | 9%; 14% | | | | | | | | 9%; 13% | | | | | | | | | 10%; 13% | | | | | | | 10%; 13% | | | | | | | | | 10%; 13% | | | | | | | | |  |  |
| NS | | | | | | | | | 0%; -1% | | | | | | | | | 0%; 0% | | | | | | | | 0%; -1% | | | | | | | | 0%; 0% | | | | | | | | | 0%; 0% | | | | | | | | | | 0%; 1% | | | | | | | | | 0%; 0% | | | | | | | | | 0%; 0% | | | | | | | | | 0%; 0% | | | | | | | | 0%; 1% | | | | | | | | 1%; 1% | | | | | | | | 1%; 2% | | | | | | | | 1%; 2% | | | | | | | | 1%; 2% | | | | | | | | 2%; 3% | | | | | | | | 3%; 4% | | | | | | | | | 3%; 4% | | | | | | | 2%; 3% | | | | | | | | | 2%; 3% | | | | | | | | |  |  |
| ON | | | | | | | | | 0%; -1% | | | | | | | | | 0%; -1% | | | | | | | | 0%; -1% | | | | | | | | 0%; -1% | | | | | | | | | 0%; -1% | | | | | | | | | | 0%; -1% | | | | | | | | | 0%; -1% | | | | | | | | | 0%; -1% | | | | | | | | | 0%; 0% | | | | | | | | 0%; 0% | | | | | | | | 0%; 0% | | | | | | | | 0%; -1% | | | | | | | | 0%; -1% | | | | | | | | 0%; -1% | | | | | | | | 0%; 0% | | | | | | | | 0%; 0% | | | | | | | | | 0%; 0% | | | | | | | 0%; 0% | | | | | | | | | 0%; 0% | | | | | | | | |  |  |
| PEI | | | | | | | | | 3%; 16% | | | | | | | | | 3%; 15% | | | | | | | | 3%; 14% | | | | | | | | 3%; 12% | | | | | | | | | 3%; 9% | | | | | | | | | | 3%; 8% | | | | | | | | | 3%; 9% | | | | | | | | | 3%; 7% | | | | | | | | | 3%; 7% | | | | | | | | 3%; 6% | | | | | | | | 4%; 7% | | | | | | | | 3%; 6% | | | | | | | | 4%; 6% | | | | | | | | 4%; 6% | | | | | | | | 4%; 6% | | | | | | | | 4%; 6% | | | | | | | | | 4%; 6% | | | | | | | 4%; 6% | | | | | | | | | 4%; 5% | | | | | | | | |  |  |
| QC | | | | | | | | | 0%; 0% | | | | | | | | | 0%; 0% | | | | | | | | 0%; 0% | | | | | | | | 0%; 0% | | | | | | | | | 0%; 0% | | | | | | | | | | 0%; 0% | | | | | | | | | 0%; 0% | | | | | | | | | 0%; 0% | | | | | | | | | 0%; 0% | | | | | | | | 0%; 0% | | | | | | | | 0%; 0% | | | | | | | | 0%; 0% | | | | | | | | 0%; 0% | | | | | | | | 0%; 0% | | | | | | | | 0%; 0% | | | | | | | | 0%; 0% | | | | | | | | | 0%; 0% | | | | | | | 0%; 0% | | | | | | | | | 0%; 0% | | | | | | | | |  |  |
| SK | | | | | | | | | 0%; 1% | | | | | | | | | 0%; 1% | | | | | | | | 0%; 1% | | | | | | | | 0%; 1% | | | | | | | | | 0%; 1% | | | | | | | | | | 0%; 1% | | | | | | | | | 0%; 1% | | | | | | | | | 0%; 0% | | | | | | | | | 1%; 1% | | | | | | | | 1%; 1% | | | | | | | | 1%; 2% | | | | | | | | 1%; 2% | | | | | | | | 1%; 2% | | | | | | | | 2%; 3% | | | | | | | | 2%; 4% | | | | | | | | 3%; 5% | | | | | | | | | 3%; 4% | | | | | | | 3%; 3% | | | | | | | | | 2%; 3% | | | | | | | | |  |  |
| **Fever and abdominal pain (Reference 6.3%)** | | | | | | | | | | | | | | | | | | | | | | | | | | | | | | | | | | | | | | | | | | | | | | | | | | | | | | | | | | | | | | | | | | | | | | | | | | | | | | | | | | | | | | | | | | | | | | | | | | | | | | | | | | | | | | | | | | | | | | | | | | | | | | | | | | | | | | | | | | | | | | | | | | | | | | | | | | | | | | | | | | | | | | | | |  |  |  |
|  | | | | | | | | 5% | | | | | | | | | 10% | | | | | | | 15% | | | | | | | | | 20% | | | | | | | | | 25% | | | | | | | | | | 30% | | | | | | | | | 35% | | | | | | | | | 40% | | | | | | | | | 45% | | | | | | | | | 50% | | | | | | | | 55% | | | | | | | | 60% | | | | | | | | 65% | | | | | | | | 70% | | | | | | | | 75% | | | | | | | | 80% | | | | | | | | | 85% | | | | | | | 90% | | | | | | | | 95% | | | | | | | | |  |  |  |
| BC | | | | | | | | 1%; 1% | | | | | | | | | 1%; 1% | | | | | | | 1%; 1% | | | | | | | | | 1%; 1% | | | | | | | | | 1%; 1% | | | | | | | | | | 1%; 1% | | | | | | | | | 1%; 1% | | | | | | | | | 1%; 1% | | | | | | | | | 1%; 1% | | | | | | | | | 1%; 1% | | | | | | | | 1%; 1% | | | | | | | | 1%; 1% | | | | | | | | 1%; 2% | | | | | | | | 1%; 2% | | | | | | | | 2%; 3% | | | | | | | | 1%; 3% | | | | | | | | | 0%; 1% | | | | | | | 0%; 0% | | | | | | | | 0%; 0% | | | | | | | | |  |  |  |
| MB | | | | | | | | 0%; 0% | | | | | | | | | 0%; 0% | | | | | | | 0%; 0% | | | | | | | | | 0%; 0% | | | | | | | | | 0%; 0% | | | | | | | | | | 0%; 0% | | | | | | | | | 0%; 0% | | | | | | | | | 0%; 0% | | | | | | | | | 0%; 0% | | | | | | | | | 0%; 0% | | | | | | | | 0%; 0% | | | | | | | | 0%; 0% | | | | | | | | 0%; 0% | | | | | | | | 0%; 0% | | | | | | | | 0%; 0% | | | | | | | | 0%; 0% | | | | | | | | | 0%; 0% | | | | | | | 0%; 0% | | | | | | | | 0%; 0% | | | | | | | | |  |  |  |
| NL | | | | | | | | 7%; 13% | | | | | | | | | 7%; 13% | | | | | | | 7%; 13% | | | | | | | | | 7%; 13% | | | | | | | | | 7%; 13% | | | | | | | | | | 7%; 13% | | | | | | | | | 7%; 13% | | | | | | | | | 7%; 13% | | | | | | | | | 6%; 13% | | | | | | | | | 6%; 12% | | | | | | | | 6%; 12% | | | | | | | | 6%; 11% | | | | | | | | 5%; 11% | | | | | | | | 6%; 11% | | | | | | | | 6%; 11% | | | | | | | | 5%; 11% | | | | | | | | | 6%; 13% | | | | | | | 6%; 12% | | | | | | | | 6%; 12% | | | | | | | | |  |  |  |
| NS | | | | | | | | 1%; 1% | | | | | | | | | 0%; 1% | | | | | | | 0%; 1% | | | | | | | | | 0%; 1% | | | | | | | | | 0%; 1% | | | | | | | | | | 1%; 1% | | | | | | | | | 0%; 1% | | | | | | | | | 0%; 1% | | | | | | | | | 0%; 0% | | | | | | | | | 0%; 0% | | | | | | | | 0%; 0% | | | | | | | | 0%; 0% | | | | | | | | 0%; 1% | | | | | | | | 0%; 1% | | | | | | | | 1%; 1% | | | | | | | | 0%; 1% | | | | | | | | | 0%; 1% | | | | | | | 0%; 0% | | | | | | | | 0%; 0% | | | | | | | | |  |  |  |
| ON | | | | | | | | 0%; -1% | | | | | | | | | 0%; 0% | | | | | | | 0%; 0% | | | | | | | | | 0%; 0% | | | | | | | | | 0%; 0% | | | | | | | | | | 0%; 0% | | | | | | | | | 0%; 0% | | | | | | | | | 0%; 0% | | | | | | | | | 0%; 0% | | | | | | | | | 0%; -1% | | | | | | | | 0%; -1% | | | | | | | | 0%; -1% | | | | | | | | 0%; -1% | | | | | | | | 0%; 0% | | | | | | | | 0%; 0% | | | | | | | | 0%; -1% | | | | | | | | | 0%; 0% | | | | | | | 0%; 0% | | | | | | | | 0%; 0% | | | | | | | | |  |  |  |
| PEI | | | | | | | | 3%; 7% | | | | | | | | | 3%; 6% | | | | | | | 3%; 6% | | | | | | | | | 3%; 6% | | | | | | | | | 3%; 6% | | | | | | | | | | 3%; 5% | | | | | | | | | 3%; 5% | | | | | | | | | 3%; 5% | | | | | | | | | 2%; 5% | | | | | | | | | 2%; 4% | | | | | | | | 2%; 4% | | | | | | | | 1%; 3% | | | | | | | | 1%; 2% | | | | | | | | 3%; 5% | | | | | | | | 3%; 6% | | | | | | | | 1%; 2% | | | | | | | | | 1%; 3% | | | | | | | 2%; 3% | | | | | | | | 2%; 4% | | | | | | | | |  |  |  |
| QC | | | | | | | | 0%; 0% | | | | | | | | | 0%; 0% | | | | | | | 0%; 0% | | | | | | | | | 0%; 0% | | | | | | | | | 0%; 0% | | | | | | | | | | 0%; 0% | | | | | | | | | 0%; 0% | | | | | | | | | 0%; 0% | | | | | | | | | 0%; 0% | | | | | | | | | 0%; 0% | | | | | | | | 0%; 0% | | | | | | | | 0%; 0% | | | | | | | | 0%; 0% | | | | | | | | 0%; 0% | | | | | | | | 0%; 0% | | | | | | | | 0%; 0% | | | | | | | | | 0%; 0% | | | | | | | 0%; 0% | | | | | | | | 0%; 0% | | | | | | | | |  |  |  |
| SK | | | | | | | | 1%; 2% | | | | | | | | | 1%; 2% | | | | | | | 1%; 1% | | | | | | | | | 1%; 2% | | | | | | | | | 0%; 1% | | | | | | | | | | 1%; 1% | | | | | | | | | 1%; 1% | | | | | | | | | 1%; 1% | | | | | | | | | 1%; 1% | | | | | | | | | 1%; 1% | | | | | | | | 1%; 1% | | | | | | | | 1%; 1% | | | | | | | | 1%; 2% | | | | | | | | 1%; 2% | | | | | | | | 2%; 3% | | | | | | | | 1%; 3% | | | | | | | | | 0%; 0% | | | | | | | 0%; 0% | | | | | | | | 0%; 0% | | | | | | | | |  |  |  |
| **Diarrhea (Reference 84.0%)** | | | | | | | | | | | | | | | | | | | | | | | | | | | | | | | | | | | | | | | | | | | | | | | | | | | | | | | | | | | | | | | | | | | | | | | | | | | | | | | | | | | | | | | | | | | | | | | | | | | | | | | | | | | | | | | | | | | | | | | | | | | | | | | | | | | | | | | | | | | | | | | | | | | | | | | | | | | | | | | | | | | | | | | |  |  |  |  |
|  |  | | | | | | 5% | | | | | | | | 10% | | | | | | | | 15% | | | | | | | | | 20% | | | | | | | | | 25% | | | | | | | | | | 30% | | | | | | | | | 35% | | | | | | | | | 40% | | | | | | | | | 45% | | | | | | | | | 50% | | | | | | | | 55% | | | | | | | | 60% | | | | | | | | 65% | | | | | | | | 70% | | | | | | | | 75% | | | | | | | | 80% | | | | | | | | 85% | | | | | | | 90% | | | | | | | | | 95% | | | | | | | | | | | | |
|  | BC | | | | | | 1%; 6% | | | | | | | | 1%; 6% | | | | | | | | 1%; 4% | | | | | | | | | 1%; 3% | | | | | | | | | 1%; 2% | | | | | | | | | | 1%; 2% | | | | | | | | | 1%; 2% | | | | | | | | | 1%; 2% | | | | | | | | | 0%; 1% | | | | | | | | | 0%; 1% | | | | | | | | 0%; 1% | | | | | | | | 1%; 1% | | | | | | | | 1%; 1% | | | | | | | | 1%; 1% | | | | | | | | 1%; 1% | | | | | | | | 1%; 1% | | | | | | | | 1%; 1% | | | | | | | 1%; 1% | | | | | | | | | 0%; 1% | | | | | | | | | | | | |
|  | MB | | | | | | 0%; 0% | | | | | | | | 0%; 0% | | | | | | | | 0%;  -1% | | | | | | | | | 0%; 0% | | | | | | | | | 0%; 0% | | | | | | | | | | 0%; 0% | | | | | | | | | 0%; 0% | | | | | | | | | 0%; 0% | | | | | | | | | 0%; 0% | | | | | | | | | 0%; 0% | | | | | | | | 0%; 0% | | | | | | | | 0%; 0% | | | | | | | | 0%; 0% | | | | | | | | 0%; 0% | | | | | | | | 0%; 0% | | | | | | | | 0%; 0% | | | | | | | | 0%; 0% | | | | | | | 0%; 0% | | | | | | | | | 0%; 0% | | | | | | | | | | | | |
|  | NL | | | | | | 1%; 6% | | | | | | | | 1%; 8% | | | | | | | | 2%; 11% | | | | | | | | | 2%; 12% | | | | | | | | | 3%; 14% | | | | | | | | | | 4%; 14% | | | | | | | | | 4%; 14% | | | | | | | | | 5%; 15% | | | | | | | | | 5%; 15% | | | | | | | | | 5%; 14% | | | | | | | | 6%; 14% | | | | | | | | 6%; 14% | | | | | | | | 7%; 15% | | | | | | | | 6%; 15% | | | | | | | | 6%; 14% | | | | | | | | 7%; 14% | | | | | | | | 7%; 14% | | | | | | | 7%; 13% | | | | | | | | | 7%; 13% | | | | | | | | | | | | |
|  | NS | | | | | | 1%; 6% | | | | | | | | 1%; 5% | | | | | | | | 1%; 4% | | | | | | | | | 0%; 2% | | | | | | | | | 0%; 1% | | | | | | | | | | 1%; 2% | | | | | | | | | 0%; 1% | | | | | | | | | 0%; 1% | | | | | | | | | 0%; 1% | | | | | | | | | 0%; 1% | | | | | | | | 0%; 1% | | | | | | | | 0%; 0% | | | | | | | | 0%; 1% | | | | | | | | 0%; 1% | | | | | | | | 1%; 1% | | | | | | | | 0%; 1% | | | | | | | | 0%; 1% | | | | | | | 0%; 1% | | | | | | | | | 0%; 0% | | | | | | | | | | | | |
|  | ON | | | | | | 0%; 0% | | | | | | | | 0%; 0% | | | | | | | | 0%;  -1% | | | | | | | | | 0%; 0% | | | | | | | | | 0%; 0% | | | | | | | | | | 0%; 0% | | | | | | | | | 0%; 0% | | | | | | | | | 0%; 0% | | | | | | | | | 0%; 0% | | | | | | | | | 0%; -1% | | | | | | | | 0%; -1% | | | | | | | | 0%; 0% | | | | | | | | 0%; 0% | | | | | | | | 0%; 0% | | | | | | | | 0%; 0% | | | | | | | | 0%; 0% | | | | | | | | 0%; -1% | | | | | | | 0%; -1% | | | | | | | | | 0%; -1% | | | | | | | | | | | | |
|  | PEI | | | | | | -3%; -44% | | | | | | | | -2%; -20% | | | | | | | | -1%; -8% | | | | | | | | | -1%; -4% | | | | | | | | | 0%; -1% | | | | | | | | | | 0%; 1% | | | | | | | | | 1%; 2% | | | | | | | | | 1%; 3% | | | | | | | | | 1%; 2% | | | | | | | | | 1%; 3% | | | | | | | | 2%; 4% | | | | | | | | 2%; 4% | | | | | | | | 2%; 5% | | | | | | | | 2%; 5% | | | | | | | | 3%; 6% | | | | | | | | 3%; 6% | | | | | | | | 3%; 7% | | | | | | | 4%; 8% | | | | | | | | | 4%; 8% | | | | | | | | | | | | |
|  | QC | | | | | | 0%; 0% | | | | | | | | 0%; 0% | | | | | | | | 0%; -1% | | | | | | | | | 0%; 0% | | | | | | | | | 0%; 0% | | | | | | | | | | 0%; 0% | | | | | | | | | 0%; 0% | | | | | | | | | 0%; 0% | | | | | | | | | 0%; 0% | | | | | | | | | 0%; 0% | | | | | | | | 0%; 0% | | | | | | | | 0%; 0% | | | | | | | | 0%; 0% | | | | | | | | 0%; 0% | | | | | | | | 0%; 0% | | | | | | | | 0%; 0% | | | | | | | | 0%; 0% | | | | | | | 0%; 0% | | | | | | | | | 0%; 0% | | | | | | | | | | | | |
|  | SK | | | | | | 1%; 8% | | | | | | | | 1%; 5% | | | | | | | | 0%; 3% | | | | | | | | | 1%; 3% | | | | | | | | | 0%; 2% | | | | | | | | | | 1%; 2% | | | | | | | | | 1%; 2% | | | | | | | | | 0%; 1% | | | | | | | | | 1%; 2% | | | | | | | | | 0%; 1% | | | | | | | | 1%; 1% | | | | | | | | 1%; 1% | | | | | | | | 0%; 1% | | | | | | | | 1%; 1% | | | | | | | | 1%; 1% | | | | | | | | 1%; 1% | | | | | | | | 1%; 2% | | | | | | | 1%; 1% | | | | | | | | | 0%; 1% | | | | | | | | | | | | |
|  | **One year old and more (Reference 98.5%)** | | | | | | | | | | | | | | | | | | | | | | | | | | | | | | | | | | | | | | | | | | | | | | | | | | | | | | | | | | | | | | | | | | | | | | | | | | | | | | | | | | | | | | | | | | | | | | | | | | | | | | | | | | | | | | | | | | | | | | | | | | | | | | | | | | | | | | | | | | | | | | | | | | | | | | | | | | | | | | | | | | | | | |  |  |  |  |  |
|  |  | | | | | 5% | | | | | | | | | | 10% | | | | | | | | | 15% | | | | | | | | | | 20% | | | | | | | | | | 25% | | | | | | | | | | 30% | | | | | | | | | 35% | | | | | | | | | 40% | | | | | | | | | 45% | | | | | | | | 50% | | | | | | | | 55% | | | | | | | | 60% | | | | | | | | 65% | | | | | | | 70% | | | | | | | | 75% | | | | | | | | 80% | | | | | | | 85% | | | | | | | 90% | | | | | | | 95% | | | | | | | | |  |  |  |  |  |
|  | BC | | | | | 0%; 7% | | | | | | | | | | 0%; 5% | | | | | | | | | 0%; 3% | | | | | | | | | | 1%; 4% | | | | | | | | | | 1%; 2% | | | | | | | | | | 1%; 2% | | | | | | | | | 0%; 1% | | | | | | | | | 0%; 1% | | | | | | | | | 0%; 1% | | | | | | | | 0%; 1% | | | | | | | | 0%; 1% | | | | | | | | 0%; 1% | | | | | | | | 0%; 1% | | | | | | | 1%; 1% | | | | | | | | 1%; 1% | | | | | | | | 1%; 1% | | | | | | | 1%; 1% | | | | | | | 1%; 1% | | | | | | | 1%; 1% | | | | | | | | |  |  |  |  |  |
|  | MB | | | | | 0%; 2% | | | | | | | | | | 0%; 5% | | | | | | | | | 0%; -1% | | | | | | | | | | 0%; 0% | | | | | | | | | | 0%; 0% | | | | | | | | | | 0%; 0% | | | | | | | | | 0%; 0% | | | | | | | | | 0%; 0% | | | | | | | | | 0%; 0% | | | | | | | | 0%; 0% | | | | | | | | 0%; 0% | | | | | | | | 0%; 0% | | | | | | | | 0%; 0% | | | | | | | 0%; 0% | | | | | | | | 0%; 0% | | | | | | | | 0%; 0% | | | | | | | 0%; 0% | | | | | | | 0%; 0% | | | | | | | 0%; 0% | | | | | | | | |  |  |  |  |  |
|  | NL | | | | | 0%; 2% | | | | | | | | | | 0%; 1% | | | | | | | | | 0%; 3% | | | | | | | | | | 1%; 4% | | | | | | | | | | 1%; 4% | | | | | | | | | | 1%; 6% | | | | | | | | | 1%; 5% | | | | | | | | | 2%; 6% | | | | | | | | | 3%; 7% | | | | | | | | 3%; 8% | | | | | | | | 3%; 7% | | | | | | | | 3%; 7% | | | | | | | | 4%; 10% | | | | | | | 4%; 10% | | | | | | | | 5%; 10% | | | | | | | | 5%; 11% | | | | | | | 6%; 12% | | | | | | | 6%; 12% | | | | | | | 6%; 12% | | | | | | | | |  |  |  |  |  |
|  | NS | | | | | 0%; 5% | | | | | | | | | | 0%; 5% | | | | | | | | | 0%; 2% | | | | | | | | | | 1%; 3% | | | | | | | | | | 1%; 2% | | | | | | | | | | 0%; 2% | | | | | | | | | 0%; 1% | | | | | | | | | 0%; 1% | | | | | | | | | 0%; 1% | | | | | | | | 0%; 1% | | | | | | | | 0%; 0% | | | | | | | | 0%; 0% | | | | | | | | 0%; 0% | | | | | | | 0%; 0% | | | | | | | | 0%; 1% | | | | | | | | 0%; 1% | | | | | | | 0%; 1% | | | | | | | 0%; 0% | | | | | | | 0%; 0% | | | | | | | | |  |  |  |  |  |
|  | ON | | | | | 0%; 2% | | | | | | | | | | 0%; 0% | | | | | | | | | 0%; -1% | | | | | | | | | | 0%; 0% | | | | | | | | | | 0%; 0% | | | | | | | | | | 0%; 0% | | | | | | | | | 0%; 0% | | | | | | | | | 0%; 0% | | | | | | | | | 0%; 0% | | | | | | | | 0%; 0% | | | | | | | | 0%; 0% | | | | | | | | 0%; 0% | | | | | | | | 0%; 0% | | | | | | | 0%; 0% | | | | | | | | 0%; 0% | | | | | | | | 0%; 0% | | | | | | | 0%; 0% | | | | | | | 0%; 0% | | | | | | | 0%; -1% | | | | | | | | |  |  |  |  |  |
|  | PEI | | | | | 48%; 1133% | | | | | | | | | | 44%; 546% | | | | | | | | | 40%; 324% | | | | | | | | | | 36%; 221% | | | | | | | | | | 32%; 157% | | | | | | | | | | 29%; 116% | | | | | | | | | 25%; 87% | | | | | | | | | 21%; 65% | | | | | | | | | 17%; 48% | | | | | | | | 14%; 37% | | | | | | | | 12%; 30% | | | | | | | | 10%; 24% | | | | | | | | 9%; 19% | | | | | | | 7%; 16% | | | | | | | | 6%; 14% | | | | | | | | 6%; 12% | | | | | | | 5%; 10% | | | | | | | 4%; 8% | | | | | | | 4%; 8% | | | | | | | | |  |  |  |  |  |
|  | QC | | | | | 0%; 0% | | | | | | | | | | 0%; 0% | | | | | | | | | 0%; 0% | | | | | | | | | | 0%; 0% | | | | | | | | | | 0%; 0% | | | | | | | | | | 0%; 0% | | | | | | | | | 0%; 0% | | | | | | | | | 0%; 0% | | | | | | | | | 0%; 0% | | | | | | | | 0%; 0% | | | | | | | | 0%; 0% | | | | | | | | 0%; 0% | | | | | | | | 0%; 0% | | | | | | | 0%; 0% | | | | | | | | 0%; 0% | | | | | | | | 0%; 0% | | | | | | | 0%; 0% | | | | | | | 0%; 0% | | | | | | | 0%; 0% | | | | | | | | |  |  |  |  |  |
|  | SK | | | | | 0%; 7% | | | | | | | | | | 0%; 5% | | | | | | | | | 0%; 2% | | | | | | | | | | 1%; 3% | | | | | | | | | | 1%; 2% | | | | | | | | | | 0%; 2% | | | | | | | | | 0%; 1% | | | | | | | | | 0%; 1% | | | | | | | | | 0%; 1% | | | | | | | | 0%; 1% | | | | | | | | 0%; 1% | | | | | | | | 1%; 1% | | | | | | | | 0%; 1% | | | | | | | 0%; 1% | | | | | | | | 0%; 1% | | | | | | | | 1%; 1% | | | | | | | 1%; 1% | | | | | | | 1%; 1% | | | | | | | 1%; 1% | | | | | | | | |  |  |  |  |  |
|  | **Admission (Reference 77.0%)** | | | | | | | | | | | | | | | | | | | | | | | | | | | | | | | | | | | | | | | | | | | | | | | | | | | | | | | | | | | | | | | | | | | | | | | | | | | | | | | | | | | | | | | | | | | | | | | | | | | | | | | | | | | | | | | | | | | | | | | | | | | | | | | | | | | | | | | | | | | | | | | | | | | | | | | | | | | | | | | | | | | | |  |  |  |  |  |  |
|  |  | | | | 5% | | | | | | | | | 10% | | | | | | | | 15% | | | | | | | | | 20% | | | | | | | | | 25% | | | | | | | | | | 30% | | | | | | | | | 35% | | | | | | | | | 40% | | | | | | | | | 45% | | | | | | | | | 50% | | | | | | | | 55% | | | | | | | | 60% | | | | | | | | 65% | | | | | | | | 70% | | | | | | | | 75% | | | | | | | | 80% | | | | | | | | 85% | | | | | | | 90% | | | | | | | | 95% | | | | | | | | |  |  |  |  |  |  |
|  | BC | | | | 6%; 52% | | | | | | | | | 7%; 47% | | | | | | | | 6%; 28% | | | | | | | | | 4%; 17% | | | | | | | | | 3%; 10% | | | | | | | | | | 2%; 5% | | | | | | | | | 1%; 4% | | | | | | | | | 1%; 3% | | | | | | | | | 1%; 2% | | | | | | | | | 1%; 1% | | | | | | | | 1%; 1% | | | | | | | | 1%; 1% | | | | | | | | 1%; 1% | | | | | | | | 1%; 1% | | | | | | | | 1%; 1% | | | | | | | | 1%; 1% | | | | | | | | 1%; 1% | | | | | | | 1%; 1% | | | | | | | | 1%; 1% | | | | | | | | |  |  |  |  |  |  |
|  | MB | | | | 0%; 0% | | | | | | | | | 0%; 0% | | | | | | | | 0%; 0% | | | | | | | | | 0%; 0% | | | | | | | | | 0%; 0% | | | | | | | | | | 0%; 0% | | | | | | | | | 0%; 0% | | | | | | | | | 0%; 0% | | | | | | | | | 0%; 0% | | | | | | | | | 0%; 0% | | | | | | | | 0%; 0% | | | | | | | | 0%; 0% | | | | | | | | 0%; 0% | | | | | | | | 0%; 0% | | | | | | | | 0%; 0% | | | | | | | | 0%; 0% | | | | | | | | 0%; 0% | | | | | | | 0%; 0% | | | | | | | | 0%; 0% | | | | | | | | |  |  |  |  |  |  |
|  | NL | | | | 4%; 37% | | | | | | | | | 4%; 26% | | | | | | | | 4%; 18% | | | | | | | | | 4%; 16% | | | | | | | | | 4%; 13% | | | | | | | | | | 5%; 13% | | | | | | | | | 4%; 10% | | | | | | | | | 4%; 10% | | | | | | | | | 4%; 9% | | | | | | | | | 4%; 9% | | | | | | | | 5%; 10% | | | | | | | | 6%; 11% | | | | | | | | 6%; 12% | | | | | | | | 6%; 12% | | | | | | | | 7%; 13% | | | | | | | | 7%; 14% | | | | | | | | 7%; 14% | | | | | | | 7%; 14% | | | | | | | | 7%; 14% | | | | | | | | |  |  |  |  |  |  |
|  | NS | | | | -3%; -23% | | | | | | | | | -2%; -13% | | | | | | | | -2%; -10% | | | | | | | | | -2%; -8% | | | | | | | | | -2%; -7% | | | | | | | | | | -2%; -6% | | | | | | | | | -2%; -6% | | | | | | | | | -2%; -5% | | | | | | | | | -2%; -4% | | | | | | | | | -2%; -3% | | | | | | | | -1%; -1% | | | | | | | | 0%; 0% | | | | | | | | 0%; 0% | | | | | | | | 0%; 0% | | | | | | | | 0%; 1% | | | | | | | | 0%; 1% | | | | | | | | 0%; 1% | | | | | | | 0%; 1% | | | | | | | | 1%; 1% | | | | | | | | |  |  |  |  |  |  |
|  | ON | | | | -9%;  -76% | | | | | | | | | -9%;  -58% | | | | | | | | -7%;  -35% | | | | | | | | | -6%;  -23% | | | | | | | | | -5%;  -16% | | | | | | | | | | -4%;  -11% | | | | | | | | | -4%;  -9% | | | | | | | | | -3%;  -7% | | | | | | | | | -3%;  -6% | | | | | | | | | -2%;  -5% | | | | | | | | -1%;  -3% | | | | | | | | -1%;  -1% | | | | | | | | 0%;  -1% | | | | | | | | 0%;  -1% | | | | | | | | 0%;  -1% | | | | | | | | 0%; 0% | | | | | | | | 0%; 0% | | | | | | | 0%; 0% | | | | | | | | 0%; 0% | | | | | | | | |  |  |  |  |  |  |
|  | PEI | | | | 5%; 41% | | | | | | | | | 7%; 42% | | | | | | | | 6%; 28% | | | | | | | | | 5%; 20% | | | | | | | | | 5%; 17% | | | | | | | | | | 5%; 14% | | | | | | | | | 4%; 11% | | | | | | | | | 4%; 9% | | | | | | | | | 4%; 8% | | | | | | | | | 3%; 6% | | | | | | | | 4%; 8% | | | | | | | | 4%; 9% | | | | | | | | 3%; 7% | | | | | | | | 3%; 6% | | | | | | | | 3%; 7% | | | | | | | | 3%; 7% | | | | | | | | 3%; 7% | | | | | | | 3%; 7% | | | | | | | | 3%; 7% | | | | | | | | |  |  |  |  |  |  |
|  | QC | | | | 0%; 0% | | | | | | | | | 0%; 0% | | | | | | | | 0%; 0% | | | | | | | | | 0%; 0% | | | | | | | | | 0%; 0% | | | | | | | | | | 0%; 0% | | | | | | | | | 0%; 0% | | | | | | | | | 0%; 0% | | | | | | | | | 0%; 0% | | | | | | | | | 0%; 0% | | | | | | | | 0%; 0% | | | | | | | | 0%; 0% | | | | | | | | 0%; 0% | | | | | | | | 0%; 0% | | | | | | | | 0%; 0% | | | | | | | | 0%; 0% | | | | | | | | 0%; 0% | | | | | | | 0%; 0% | | | | | | | | 0%; 0% | | | | | | | | |  |  |  |  |  |  |
|  | SK | | | | 6%; 53% | | | | | | | | | 7%; 46% | | | | | | | | 6%; 27% | | | | | | | | | 4%; 16% | | | | | | | | | 3%; 10% | | | | | | | | | | 2%; 6% | | | | | | | | | 1%; 3% | | | | | | | | | 1%; 2% | | | | | | | | | 1%; 2% | | | | | | | | | 1%; 1% | | | | | | | | 1%; 1% | | | | | | | | 1%; 1% | | | | | | | | 1%; 1% | | | | | | | | 1%; 1% | | | | | | | | 1%; 1% | | | | | | | | 1%; 1% | | | | | | | | 1%; 1% | | | | | | | 0%; 1% | | | | | | | | 1%; 1% | | | | | | | | |  |  |  |  |  |  |
|  | **Four weeks recurrence (Reference 18.4%)** | | | | | | | | | | | | | | | | | | | | | | | | | | | | | | | | | | | | | | | | | | | | | | | | | | | | | | | | | | | | | | | | | | | | | | | | | | | | | | | | | | | | | | | | | | | | | | | | | | | | | | | | | | | | | | | | | | | | | | | | | | | | | | | | | | | | | | | | | | | | | | | | | | | | | | | | | | | | | | | | | | | |  |  |  |  |  |  |  |
|  |  | | | 5% | | | | | | | | | 10% | | | | | | | 15% | | | | | | | | 20% | | | | | | | | | 25% | | | | | | | | | 30% | | | | | | | | | | 35% | | | | | | | | | 40% | | | | | | | | | 45% | | | | | | | | | 50% | | | | | | | | 55% | | | | | | | | 60% | | | | | | | | 65% | | | | | | | | 70% | | | | | | | | 75% | | | | | | | | 80% | | | | | | | | 85% | | | | | | | | 90% | | | | | | | | 95% | | | | | | | | | | | | | | | | |
|  | BC | | | 1%; 1% | | | | | | | | | 1%; 1% | | | | | | | 1%; 1% | | | | | | | | 1%; 1% | | | | | | | | | 1%; 2% | | | | | | | | | 1%; 2% | | | | | | | | | | 1%; 1% | | | | | | | | | 0%; 1% | | | | | | | | | 1%; 1% | | | | | | | | | 0%; 1% | | | | | | | | 1%; 2% | | | | | | | | 0%; 1% | | | | | | | | 0%; 1% | | | | | | | | 0%; 1% | | | | | | | | 0%; 1% | | | | | | | | 0%; 1% | | | | | | | | 0%; 1% | | | | | | | | 0%; 1% | | | | | | | | 0%; -2% | | | | | | | | | | | | | | | | |
|  | MB | | | 0%; 0% | | | | | | | | | 0%; 0% | | | | | | | 0%; 0% | | | | | | | | 0%; 0% | | | | | | | | | 0%; 0% | | | | | | | | | 0%; 0% | | | | | | | | | | 0%; 0% | | | | | | | | | 0%; 0% | | | | | | | | | 0%; 0% | | | | | | | | | 0%; 0% | | | | | | | | 0%; 0% | | | | | | | | 0%; 0% | | | | | | | | 0%; 0% | | | | | | | | 0%; 0% | | | | | | | | 0%; 1% | | | | | | | | 0%; -1% | | | | | | | | 0%; 0% | | | | | | | | 0%; 0% | | | | | | | | 0%; -2% | | | | | | | | | | | | | | | | |
|  | NL | | | 2%; 4% | | | | | | | | | 4%; 7% | | | | | | | 6%; 11% | | | | | | | | 7%; 15% | | | | | | | | | 10%; 21% | | | | | | | | | 12%; 26% | | | | | | | | | | 14%; 33% | | | | | | | | | 17%; 42% | | | | | | | | | 19%; 51% | | | | | | | | | 22%; 64% | | | | | | | | 24%; 77% | | | | | | | | 27%; 95% | | | | | | | | 30%; 119% | | | | | | | | 34%; 151% | | | | | | | | 37%; 196% | | | | | | | | 40%; 258% | | | | | | | | 44%; 361% | | | | | | | | 47%; 543% | | | | | | | | 51%; 1071% | | | | | | | | | | | | | | | | |
|  | NS | | | 0%; 1% | | | | | | | | | 0%; 0% | | | | | | | 0%; 1% | | | | | | | | 0%; 1% | | | | | | | | | 0%; 1% | | | | | | | | | 0%; 1% | | | | | | | | | | 0%; 1% | | | | | | | | | 0%; 1% | | | | | | | | | 0%; 1% | | | | | | | | | 0%; 1% | | | | | | | | 0%; 1% | | | | | | | | 0%; 1% | | | | | | | | 0%; 1% | | | | | | | | 0%; 1% | | | | | | | | 0%; 2% | | | | | | | | 0%; 0% | | | | | | | | 0%; 1% | | | | | | | | 0%; 0% | | | | | | | | 0%; -2% | | | | | | | | | | | | | | | | |
|  | ON | | | 0%; -1% | | | | | | | | | 0%; -1% | | | | | | | 0%; 0% | | | | | | | | 0%; -1% | | | | | | | | | 0%; 0% | | | | | | | | | 0%; 0% | | | | | | | | | | 0%; 0% | | | | | | | | | 0%; 0% | | | | | | | | | 0%; 0% | | | | | | | | | 0%; 0% | | | | | | | | 0%; 0% | | | | | | | | 0%; 0% | | | | | | | | 0%; 0% | | | | | | | | 0%; 0% | | | | | | | | 0%; 0% | | | | | | | | 0%; 0% | | | | | | | | 0%; 0% | | | | | | | | 0%; 0% | | | | | | | | 0%; -2% | | | | | | | | | | | | | | | | |
|  | PEI | | | 4%; 8% | | | | | | | | | 4%; 7% | | | | | | | 4%; 7% | | | | | | | | 4%; 8% | | | | | | | | | 3%; 7% | | | | | | | | | 2%; 5% | | | | | | | | | | 2%; 5% | | | | | | | | | 2%; 5% | | | | | | | | | 2%; 5% | | | | | | | | | 1%; 4% | | | | | | | | 1%; 4% | | | | | | | | 1%; 4% | | | | | | | | 1%; 3% | | | | | | | | 1%; 4% | | | | | | | | 1%; 4% | | | | | | | | 0%; 2% | | | | | | | | 0%; 2% | | | | | | | | 0%; 2% | | | | | | | | 0%; 4% | | | | | | | | | | | | | | | | |
|  | QC | | | 0%; 0% | | | | | | | | | 0%; 0% | | | | | | | 0%; 0% | | | | | | | | 0%; 0% | | | | | | | | | 0%; 0% | | | | | | | | | 0%; 0% | | | | | | | | | | 0%; 0% | | | | | | | | | 0%; 0% | | | | | | | | | 0%; 0% | | | | | | | | | 0%; 0% | | | | | | | | 0%; 0% | | | | | | | | 0%; 0% | | | | | | | | 0%; 0% | | | | | | | | 0%; 0% | | | | | | | | 0%; 0% | | | | | | | | 0%; 0% | | | | | | | | 0%; 0% | | | | | | | | 0%; 0% | | | | | | | | 0%; -2% | | | | | | | | | | | | | | | | |
|  | SK | | | 1%; 1% | | | | | | | | | 1%; 1% | | | | | | | 1%; 2% | | | | | | | | 1%; 1% | | | | | | | | | 0%; 1% | | | | | | | | | 1%; 1% | | | | | | | | | | 0%; 1% | | | | | | | | | 0%; 1% | | | | | | | | | 1%; 2% | | | | | | | | | 0%; 1% | | | | | | | | 0%; 1% | | | | | | | | 0%; 1% | | | | | | | | 0%; 1% | | | | | | | | 0%; 0% | | | | | | | | 0%; 1% | | | | | | | | 0%; 1% | | | | | | | | 0%; 1% | | | | | | | | 0%; 1% | | | | | | | | 0%; -2% | | | | | | | | | | | | | | | | |
|  | **Hospitalisation within four weeks (Reference 18.5%)** | | | | | | | | | | | | | | | | | | | | | | | | | | | | | | | | | | | | | | | | | | | | | | | | | | | | | | | | | | | | | | | | | | | | | | | | | | | | | | | | | | | | | | | | | | | | | | | | | | | | | | | | | | | | | | | | | | | | | | | | | | | | | | | | | | | | | | | | | | | | | | | | | | | | | | | | | | | | | | | | | | |  |  |  |  |  |  |  |  |
|  |  | | 5% | | | | | | | | | 10% | | | | | | | | | 15% | | | | | | | | 20% | | | | | | | | | 25% | | | | | | | | | 30% | | | | | | | | | | 35% | | | | | | | | | 40% | | | | | | | | | 45% | | | | | | | | | 50% | | | | | | | | 55% | | | | | | | | 60% | | | | | | | | 65% | | | | | | | | 70% | | | | | | | | 75% | | | | | | | | | 80% | | | | | | | | 85% | | | | | | | 90% | | | | | | | | 95% | | | | | | | |  |  |  |  |  |  |  |  |
|  | BC | | 1%; 2% | | | | | | | | | 1%; 1% | | | | | | | | | 1%; 1% | | | | | | | | 1%; 2% | | | | | | | | | 1%; 2% | | | | | | | | | 1%; 1% | | | | | | | | | | 1%; 1% | | | | | | | | | 1%; 1% | | | | | | | | | 1%; 2% | | | | | | | | | 1%; 1% | | | | | | | | 1%; 1% | | | | | | | | 1%; 1% | | | | | | | | 1%; 1% | | | | | | | | 1%; 1% | | | | | | | | 1%; 1% | | | | | | | | | 1%; 2% | | | | | | | | 3%; 4% | | | | | | | 6%; 8% | | | | | | | | 9%; 12% | | | | | | | |  |  |  |  |  |  |  |  |
|  | MB | | 0%; 0% | | | | | | | | | 0%; 0% | | | | | | | | | 0%; 0% | | | | | | | | 0%; 0% | | | | | | | | | 0%; 0% | | | | | | | | | 0%; 0% | | | | | | | | | | 0%; 0% | | | | | | | | | 0%; 0% | | | | | | | | | 0%; 0% | | | | | | | | | 0%; 0% | | | | | | | | 0%; 0% | | | | | | | | 0%; 0% | | | | | | | | 0%; 0% | | | | | | | | 0%; 0% | | | | | | | | 0%; 0% | | | | | | | | | 0%; 0% | | | | | | | | 0%; 0% | | | | | | | 0%; 0% | | | | | | | | 0%; 0% | | | | | | | |  |  |  |  |  |  |  |  |
|  | NL | | 5%; 11% | | | | | | | | | 6%; 12% | | | | | | | | | 6%; 13% | | | | | | | | 7%; 14% | | | | | | | | | 7%; 14% | | | | | | | | | 8%; 15% | | | | | | | | | | 8%; 15% | | | | | | | | | 9%; 16% | | | | | | | | | 10%; 16% | | | | | | | | | 10%; 17% | | | | | | | | 10%; 17% | | | | | | | | 11%; 17% | | | | | | | | 11%; 17% | | | | | | | | 12%; 17% | | | | | | | | 12%; 17% | | | | | | | | | 12%; 17% | | | | | | | | 12%; 17% | | | | | | | 12%; 17% | | | | | | | | 12%; 17% | | | | | | | |  |  |  |  |  |  |  |  |
|  | NS | | 1%; 2% | | | | | | | | | 1%; 1% | | | | | | | | | 0%; 1% | | | | | | | | 0%; 0% | | | | | | | | | 1%; 1% | | | | | | | | | 0%; 0% | | | | | | | | | | 0%; 0% | | | | | | | | | 0%; 0% | | | | | | | | | 0%; 0% | | | | | | | | | 0%;  -1% | | | | | | | | -1%;  -1% | | | | | | | | -1%;  -1% | | | | | | | | -1%;  -2% | | | | | | | | -2%;  -3% | | | | | | | | -3%;  -5% | | | | | | | | | -4%;  -6% | | | | | | | | -4%;  -6% | | | | | | | -4%;  -5% | | | | | | | | -3%;  -5% | | | | | | | |  |  |  |  |  |  |  |  |
|  | ON | | 0%; 0% | | | | | | | | | 0%; 0% | | | | | | | | | 0%; 0% | | | | | | | | 0%;  -1% | | | | | | | | | 0%;  -1% | | | | | | | | | 0%;  -1% | | | | | | | | | | -1%;  -1% | | | | | | | | | -1%;  -1% | | | | | | | | | -1%;  -1% | | | | | | | | | -1%;  -2% | | | | | | | | -1%;  -2% | | | | | | | | -2%;  -3% | | | | | | | | -2%;  -3% | | | | | | | | -3%;  -4% | | | | | | | | -4%;  -6% | | | | | | | | | -5%;  -7% | | | | | | | | -5%;  -7% | | | | | | | -5%;  -7% | | | | | | | | -5%;  -7% | | | | | | | |  |  |  |  |  |  |  |  |
|  | PEI | | 4%; 10% | | | | | | | | | 4%; 9% | | | | | | | | | 3%; 7% | | | | | | | | 3%; 7% | | | | | | | | | 3%; 5% | | | | | | | | | 3%; 6% | | | | | | | | | | 2%; 4% | | | | | | | | | 2%; 4% | | | | | | | | | 2%; 4% | | | | | | | | | 2%; 4% | | | | | | | | 2%; 3% | | | | | | | | 2%; 3% | | | | | | | | 2%; 3% | | | | | | | | 2%; 2% | | | | | | | | 1%; 2% | | | | | | | | | 1%; 1% | | | | | | | | 1%; 1% | | | | | | | 1%; 1% | | | | | | | | 1%; 1% | | | | | | | |  |  |  |  |  |  |  |  |
|  | QC | | 0%; 0% | | | | | | | | | 0%; 0% | | | | | | | | | 0%; 0% | | | | | | | | 0%; 0% | | | | | | | | | 0%; 0% | | | | | | | | | 0%; 0% | | | | | | | | | | 0%; 0% | | | | | | | | | 0%; 0% | | | | | | | | | 0%; 0% | | | | | | | | | 0%; 0% | | | | | | | | 0%; 0% | | | | | | | | 0%; 0% | | | | | | | | 0%; 0% | | | | | | | | 0%; 0% | | | | | | | | 0%; 0% | | | | | | | | | 0%; 0% | | | | | | | | 0%; 0% | | | | | | | 0%; 0% | | | | | | | | 0%; 0% | | | | | | | |  |  |  |  |  |  |  |  |
|  | SK | | 1%; 2% | | | | | | | | | 1%; 1% | | | | | | | | | 1%; 1% | | | | | | | | 1%; 1% | | | | | | | | | 1%; 1% | | | | | | | | | 1%; 1% | | | | | | | | | | 1%; 1% | | | | | | | | | 1%; 2% | | | | | | | | | 1%; 1% | | | | | | | | | 1%; 2% | | | | | | | | 1%; 1% | | | | | | | | 1%; 1% | | | | | | | | 1%; 2% | | | | | | | | 1%; 1% | | | | | | | | 1%; 1% | | | | | | | | | 1%; 2% | | | | | | | | 3%; 4% | | | | | | | 6%; 8% | | | | | | | | 9%; 12% | | | | | | | |  |  |  |  |  |  |  |  |
|  | **Information on diarrhea available (Reference 83%)** | | | | | | | | | | | | | | | | | | | | | | | | | | | | | | | | | | | | | | | | | | | | | | | | | | | | | | | | | | | | | | | | | | | | | | | | | | | | | | | | | | | | | | | | | | | | | | | | | | | | | | | | | | | | | | | | | | | | | | | | | | | | | | | | | | | | | | | | | | | | | | | | | | | | | | | | | | | | | | | | | |  |  |  |  |  |  |  |  |  |
|  |  | 5% | | | | | | | | | 10% | | | | | | | | | | 15% | | | | | | | | | 20% | | | | | | | | | 25% | | | | | | | | | | 30% | | | | | | | | | 35% | | | | | | | | | 40% | | | | | | | | | 45% | | | | | | | | | 50% | | | | | | | | 55% | | | | | | | | 60% | | | | | | | | 65% | | | | | | | | 70% | | | | | | | | 75% | | | | | | | 80% | | | | | | | | 85% | | | | | | | 90% | | | | | | | 95% | | | | | | | | |  |  |  |  |  |  |  |  |  |
|  | BC | 1%; 14% | | | | | | | | | 0%; 1% | | | | | | | | | | 0%; 0% | | | | | | | | | 0%; 0% | | | | | | | | | 0%; 0% | | | | | | | | | | 0%; 0% | | | | | | | | | 0%; 0% | | | | | | | | | 0%; 0% | | | | | | | | | 0%; 0% | | | | | | | | | 0%; 0% | | | | | | | | 2%; 3% | | | | | | | | 3%; 4% | | | | | | | | 2%; 4% | | | | | | | | 2%; 3% | | | | | | | | 1%; 2% | | | | | | | 1%; 2% | | | | | | | | 1%; 1% | | | | | | | 0%; 1% | | | | | | | 0%; 0% | | | | | | | | |  |  |  |  |  |  |  |  |  |
|  | MB | 0%; 0% | | | | | | | | | 0%; 0% | | | | | | | | | | 0%; 0% | | | | | | | | | 0%; 0% | | | | | | | | | 0%; 0% | | | | | | | | | | 0%; 0% | | | | | | | | | 0%; 0% | | | | | | | | | 0%; 0% | | | | | | | | | 0%; 0% | | | | | | | | | 0%; 0% | | | | | | | | 0%; 0% | | | | | | | | 0%; 0% | | | | | | | | 0%; 0% | | | | | | | | 0%;  -1% | | | | | | | | 0%; 0% | | | | | | | 0%; 0% | | | | | | | | 0%; 0% | | | | | | | 0%; 0% | | | | | | | 0%; 0% | | | | | | | | |  |  |  |  |  |  |  |  |  |
|  | NL | 0%; 1% | | | | | | | | | 0%; 2% | | | | | | | | | | 0%; 0% | | | | | | | | | 1%; 3% | | | | | | | | | 2%; 5% | | | | | | | | | | 3%; 5% | | | | | | | | | 4%; 6% | | | | | | | | | 4%; 7% | | | | | | | | | 5%; 8% | | | | | | | | | 6%; 10% | | | | | | | | 6%; 10% | | | | | | | | 7%; 11% | | | | | | | | 7%; 12% | | | | | | | | 7%; 12% | | | | | | | | 7%; 13% | | | | | | | 7%; 13% | | | | | | | | 7%; 14% | | | | | | | 6%; 13% | | | | | | | 7%; 14% | | | | | | | | |  |  |  |  |  |  |  |  |  |
|  | NS | 1%; 8% | | | | | | | | | 0%; 1% | | | | | | | | | | 0%; 0% | | | | | | | | | 0%; 0% | | | | | | | | | 0%; 0% | | | | | | | | | | 0%;  -1% | | | | | | | | | 0%;  -1% | | | | | | | | | -1%;  -1% | | | | | | | | | -1%;  -1% | | | | | | | | | 0%; 0% | | | | | | | | 1%; 2% | | | | | | | | 2%; 3% | | | | | | | | 2%; 3% | | | | | | | | 1%; 2% | | | | | | | | 1%; 1% | | | | | | | 1%; 1% | | | | | | | | 0%; 0% | | | | | | | 0%; 0% | | | | | | | 0%; 0% | | | | | | | | |  |  |  |  |  |  |  |  |  |
|  | ON | 0%; 1% | | | | | | | | | 0%; 0% | | | | | | | | | | 0%; 0% | | | | | | | | | 0%; 0% | | | | | | | | | 0%; 0% | | | | | | | | | | 0%;  -1% | | | | | | | | | 0%;  -1% | | | | | | | | | 0%;  -1% | | | | | | | | | 0%; 0% | | | | | | | | | 0%; 0% | | | | | | | | -1%;  -1% | | | | | | | | -1%;  -1% | | | | | | | | 0%;  -1% | | | | | | | | 0%;  -1% | | | | | | | | 0%;  -1% | | | | | | | 0%; 0% | | | | | | | | 0%; 0% | | | | | | | 0%; 0% | | | | | | | 0%; 0% | | | | | | | | |  |  |  |  |  |  |  |  |  |
|  | PEI | 1%; 10% | | | | | | | | | 11%; 61% | | | | | | | | | | 11%; 34% | | | | | | | | | 12%; 29% | | | | | | | | | 11%; 23% | | | | | | | | | | 9%; 16% | | | | | | | | | 8%; 14% | | | | | | | | | 7%; 12% | | | | | | | | | 6%; 11% | | | | | | | | | 6%; 10% | | | | | | | | 6%; 9% | | | | | | | | 5%; 8% | | | | | | | | 4%; 7% | | | | | | | | 4%; 7% | | | | | | | | 3%; 7% | | | | | | | 4%; 7% | | | | | | | | 3%; 6% | | | | | | | 3%; 6% | | | | | | | 4%; 8% | | | | | | | | |  |  |  |  |  |  |  |  |  |
|  | QC | 0%; 1% | | | | | | | | | 0%; -1% | | | | | | | | | | 0%; -1% | | | | | | | | | 0%; 0% | | | | | | | | | 0%; 0% | | | | | | | | | | 0%; 0% | | | | | | | | | 0%; 0% | | | | | | | | | 0%; 0% | | | | | | | | | 0%; 0% | | | | | | | | | 0%; 0% | | | | | | | | 0%; 0% | | | | | | | | 0%; 0% | | | | | | | | 0%; 0% | | | | | | | | 0%; 0% | | | | | | | | 0%; 0% | | | | | | | 0%; 0% | | | | | | | | 0%; 0% | | | | | | | 0%; 0% | | | | | | | 0%; 0% | | | | | | | | |  |  |  |  |  |  |  |  |  |
|  | SK | 1%; 14% | | | | | | | | | 0%; 1% | | | | | | | | | | -1%; -2% | | | | | | | | | 0%; 0% | | | | | | | | | 0%; 0% | | | | | | | | | | 0%; 0% | | | | | | | | | 0%; 0% | | | | | | | | | 0%; 0% | | | | | | | | | 0%; 0% | | | | | | | | | 0%; 0% | | | | | | | | 2%; 3% | | | | | | | | 2%; 4% | | | | | | | | 2%; 4% | | | | | | | | 0%; 0% | | | | | | | | 1%; 2% | | | | | | | 1%; 1% | | | | | | | | 0%; 1% | | | | | | | 0%; 0% | | | | | | | 0%; 0% | | | | | | | | |  |  |  |  |  |  |  |  |  |
|  | Absolute difference (province – CNISP); Relative difference (province – CNISP)/CNISP | | | | | | | | | | | | | | | | | | | | | | | | | | | | | | | | | | | | | | | | | | | | | | |  |  |  |  |  |  |  |  |  |  |  |  |  |  |  |  |  |  |  |  |  |  |  |  |  |  |  |  |  |  |  |  |  |  |  |  |  |  |  |  |  |  |  |  |  |  |  |  |  |  |  |  |  |  |  |  |  |  |  |  |  |  |  |  |  |  |  |  |  |  |  |  |  |  |  |  |  |  |  |  |  |  |  |  |  |  |  |  |  |  |  |  |  |  |  |  |  |  |  |  |  |  |  |  |  |  |  |  |  |  |  |  |  |  |  |  |  |  |  |  |  |  |  |  |
|  | Comparable | | | | | | | | | | | | | | | | | | | | | | | | | | | | | | | | | | | | | | | | | | | | | | |  |  |  |  |  |  |  |  |  |  |  |  |  |  |  |  |  |  |  |  |  |  |  |  |  |  |  |  |  |  |  |  |  |  |  |  |  |  |  |  |  |  |  |  |  |  |  |  |  |  |  |  |  |  |  |  |  |  |  |  |  |  |  |  |  |  |  |  |  |  |  |  |  |  |  |  |  |  |  |  |  |  |  |  |  |  |  |  |  |  |  |  |  |  |  |  |  |  |  |  |  |  |  |  |  |  |  |  |  |  |  |  |  |  |  |  |  |  |  |  |  |  |  |  |
|  | Province not comparable with CNISP | | | | | | | | | | | | | | | | | | | | | | | | | | | | | | | | | | | | | | | | | | | | | | |  |  |  |  |  |  |  |  |  |  |  |  |  |  |  |  |  |  |  |  |  |  |  |  |  |  |  |  |  |  |  |  |  |  |  |  |  |  |  |  |  |  |  |  |  |  |  |  |  |  |  |  |  |  |  |  |  |  |  |  |  |  |  |  |  |  |  |  |  |  |  |  |  |  |  |  |  |  |  |  |  |  |  |  |  |  |  |  |  |  |  |  |  |  |  |  |  |  |  |  |  |  |  |  |  |  |  |  |  |  |  |  |  |  |  |  |  |  |  |  |  |  |  |  |

**Table S8-** Distribution of absolute and relative differences of proportions of patients meeting case definition: provinces with varying proportions of elements vs. elements fixed at their reference proportions for CNISP, by province MRSA

| **Three days of admission (Reference 34.0%)** | | | | | | | | | | | | | | | | | | | | | | | | | | | | | | | | | | | | | | | | | | | | | | | | | | | | | | | | | | | | | | | | | | | | | | | | | | | | | | | | | | | | | | | | | | | | | | | | | | | | | | | | | | | | | | |  |
| --- | --- | --- | --- | --- | --- | --- | --- | --- | --- | --- | --- | --- | --- | --- | --- | --- | --- | --- | --- | --- | --- | --- | --- | --- | --- | --- | --- | --- | --- | --- | --- | --- | --- | --- | --- | --- | --- | --- | --- | --- | --- | --- | --- | --- | --- | --- | --- | --- | --- | --- | --- | --- | --- | --- | --- | --- | --- | --- | --- | --- | --- | --- | --- | --- | --- | --- | --- | --- | --- | --- | --- | --- | --- | --- | --- | --- | --- | --- | --- | --- | --- | --- | --- | --- | --- | --- | --- | --- | --- | --- | --- | --- | --- | --- | --- | --- | --- | --- | --- | --- | --- | --- | --- | --- | --- | --- | --- | --- | --- | --- | --- |
|  | | | | | | | 5% | | | | | | 10% | | | | | | 15% | | | | | | 20% | | | | | | 25% | | | | | | 30% | | | | | | 35% | | | | | | 40% | | | | | | 45% | | | | | | 50% | | | | | 55% | | | | | 60% | | | | | 65% | | | | | 70% | | | | | 75% | | | | | 80% | | | | | 85% | | | | | 90% | | | | | 95% | | | | |  |
| BC | | | | | | | 7%; 13% | | | | | | 7%; 13% | | | | | | 8%; 13% | | | | | | 8%; 13% | | | | | | 8%; 13% | | | | | | 8%; 13% | | | | | | 8%; 13% | | | | | | 8%; 13% | | | | | | 8%; 14% | | | | | | 9%; 15% | | | | | 9%; 15% | | | | | 10%; 17% | | | | | 10%; 18% | | | | | 12%; 20% | | | | | 14%; 24% | | | | | 17%; 30% | | | | | 21%; 35% | | | | | 24%; 42% | | | | | 29%; 49% | | | | |  |
| MB | | | | | | | 8%; 13% | | | | | | 8%; 13% | | | | | | 7%; 13% | | | | | | 7%; 13% | | | | | | 7%; 13% | | | | | | 8%; 13% | | | | | | 8%; 13% | | | | | | 8%; 14% | | | | | | 8%; 14% | | | | | | 8%; 14% | | | | | 9%; 15% | | | | | 9%; 16% | | | | | 10%; 17% | | | | | 12%; 20% | | | | | 14%; 24% | | | | | 17%; 30% | | | | | 21%; 36% | | | | | 24%; 41% | | | | | 28%; 49% | | | | |  |
| NL | | | | | | | 8%; 13% | | | | | | 8%; 13% | | | | | | 8%; 14% | | | | | | 8%; 14% | | | | | | 8%; 14% | | | | | | 9%; 15% | | | | | | 9%; 15% | | | | | | 9%; 16% | | | | | | 10%; 17% | | | | | | 11%; 18% | | | | | 11%; 19% | | | | | 13%; 22% | | | | | 14%; 24% | | | | | 16%; 27% | | | | | 19%; 32% | | | | | 22%; 38% | | | | | 26%; 45% | | | | | 31%; 53% | | | | | 36%; 61% | | | | |  |
| NS | | | | | | | 7%; 12% | | | | | | 9%; 15% | | | | | | 9%; 15% | | | | | | 8%; 14% | | | | | | 8%; 14% | | | | | | 9%; 16% | | | | | | 9%; 15% | | | | | | 9%; 16% | | | | | | 10%; 17% | | | | | | 10%; 17% | | | | | 11%; 19% | | | | | 12%; 21% | | | | | 14%; 24% | | | | | 16%; 27% | | | | | 19%; 32% | | | | | 23%; 39% | | | | | 27%; 46% | | | | | 31%; 53% | | | | | 35%; 61% | | | | |  |
| ON | | | | | | | -55%; -95% | | | | | | -55%; -95% | | | | | | -55%; -95% | | | | | | -55%; -95% | | | | | | -55%; -95% | | | | | | -55%; -95% | | | | | | -55%; -95% | | | | | | -55%; -95% | | | | | | -55%; -94% | | | | | | -54%; -93% | | | | | -54%; -93% | | | | | -53%; -92% | | | | | -53%; -91% | | | | | -51%; -88% | | | | | -50%; -87% | | | | | -51%; -88% | | | | | -52%; -89% | | | | | -52%; -90% | | | | | -53%; -91% | | | | |  |
| PEI | | | | | | | 7%; 12% | | | | | | 8%; 13% | | | | | | 8%; 14% | | | | | | 8%; 14% | | | | | | 8%; 14% | | | | | | 8%; 14% | | | | | | 9%; 15% | | | | | | 9%; 16% | | | | | | 10%; 17% | | | | | | 11%; 19% | | | | | 11%; 19% | | | | | 13%; 22% | | | | | 14%; 24% | | | | | 16%; 27% | | | | | 18%; 31% | | | | | 23%; 39% | | | | | 27%; 46% | | | | | 31%; 54% | | | | | 36%; 62% | | | | |  |
| QC | | | | | | | -2%; -3% | | | | | | -2%; -3% | | | | | | -2%; -3% | | | | | | -2%; -3% | | | | | | -2%; -3% | | | | | | -2%; -3% | | | | | | -2%; -3% | | | | | | -2%; -3% | | | | | | -2%; -3% | | | | | | -1%; -1% | | | | | 0%; -1% | | | | | 1%; 1% | | | | | 2%; 4% | | | | | 5%; 8% | | | | | 8%; 13% | | | | | 9%; 16% | | | | | 11%; 19% | | | | | 14%; 24% | | | | | 17%; 29% | | | | |  |
| **Mother status (Reference 5.5%)** | | | | | | | | | | | | | | | | | | | | | | | | | | | | | | | | | | | | | | | | | | | | | | | | | | | | | | | | | | | | | | | | | | | | | | | | | | | | | | | | | | | | | | | | | | | | | | | | | | | | | | | | | | | | | | |  |
|  | | | | | | 5% | | | | | | 10% | | | | | | 15% | | | | | | 20% | | | | | | 25% | | | | | | 30% | | | | | | 35% | | | | | | 40% | | | | | | 45% | | | | | | 50% | | | | | | 55% | | | | | 60% | | | | | 65% | | | | | 70% | | | | | 75% | | | | | 80% | | | | | 85% | | | | | 90% | | | | | 95% | | | | |  |
| BC | | | | | | 8%; 13% | | | | | | 5%; 9% | | | | | | 3%; 6% | | | | | | 4%; 6% | | | | | | 5%; 9% | | | | | | 6%; 11% | | | | | | 6%; 11% | | | | | | 2%; 3% | | | | | | -2%; -3% | | | | | | -5%; -8% | | | | | | -9%; -15% | | | | | -13%; -22% | | | | | -18%; -31% | | | | | -24%; -41% | | | | | -29%; -50% | | | | | -34%; -59% | | | | | -40%; -68% | | | | | -46%; -78% | | | | | -51%; -88% | | | | |  |
| MB | | | | | | 8%; 13% | | | | | | 5%; 9% | | | | | | 4%; 6% | | | | | | 4%; 6% | | | | | | 5%; 9% | | | | | | 6%; 11% | | | | | | 6%; 10% | | | | | | 2%; 3% | | | | | | -2%; -3% | | | | | | -5%; -8% | | | | | | -8%; -14% | | | | | -13%; -22% | | | | | -18%; -31% | | | | | -23%; -40% | | | | | -29%; -50% | | | | | -34%; -59% | | | | | -39%; -68% | | | | | -45%; -78% | | | | | -51%; -88% | | | | |  |
| NL | | | | | | 9%; 16% | | | | | | 7%; 12% | | | | | | 5%; 9% | | | | | | 6%; 11% | | | | | | 7%; 13% | | | | | | 8%; 14% | | | | | | 9%; 15% | | | | | | 6%; 11% | | | | | | 5%; 8% | | | | | | 3%; 5% | | | | | | 1%; 1% | | | | | -3%; -5% | | | | | -6%; -11% | | | | | -11%; -19% | | | | | -15%; -25% | | | | | -18%; -30% | | | | | -21%; -36% | | | | | -25%; -42% | | | | | -28%; -48% | | | | |  |
| NS | | | | | | 10%; 17% | | | | | | 8%; 13% | | | | | | 5%; 9% | | | | | | 6%; 10% | | | | | | 8%; 13% | | | | | | 9%; 15% | | | | | | 8%; 13% | | | | | | 7%; 12% | | | | | | 4%; 7% | | | | | | 2%; 4% | | | | | | 2%; 3% | | | | | -1%; -2% | | | | | -5%; -9% | | | | | -11%; -18% | | | | | -15%; -25% | | | | | -16%; -28% | | | | | -22%; -37% | | | | | -25%; -44% | | | | | -28%; -48% | | | | |  |
| ON | | | | | | -55%; -95% | | | | | | -55%; -95% | | | | | | -55%; -95% | | | | | | -55%; -95% | | | | | | -55%; -95% | | | | | | -55%; -95% | | | | | | -55%; -95% | | | | | | -56%; -96% | | | | | | -55%; -95% | | | | | | -55%; -94% | | | | | | -54%; -94% | | | | | -54%; -93% | | | | | -54%; -92% | | | | | -53%; -92% | | | | | -53%; -91% | | | | | -53%; -91% | | | | | -52%; -90% | | | | | -52%; -89% | | | | | -51%; -88% | | | | |  |
| PEI | | | | | | 9%; 15% | | | | | | 7%; 12% | | | | | | 6%; 10% | | | | | | 7%; 12% | | | | | | 8%; 14% | | | | | | 9%; 15% | | | | | | 9%; 16% | | | | | | 7%; 12% | | | | | | 5%; 8% | | | | | | 2%; 4% | | | | | | 1%; 2% | | | | | -2%; -4% | | | | | -6%; -10% | | | | | -10%; -18% | | | | | -14%; -24% | | | | | -17%; -30% | | | | | -21%; -37% | | | | | -25%; -43% | | | | | -26%; -45% | | | | |  |
| QC | | | | | | -1%; -2% | | | | | | 0%; 0% | | | | | | 3%; 4% | | | | | | 6%; 10% | | | | | | 9%; 16% | | | | | | 13%; 23% | | | | | | 15%; 26% | | | | | | 14%; 25% | | | | | | 12%; 21% | | | | | | 9%; 16% | | | | | | 7%; 12% | | | | | 3%; 6% | | | | | -1%; -1% | | | | | -5%; -9% | | | | | -12%; -20% | | | | | -20%; -35% | | | | | -29%; -49% | | | | | -38%; -65% | | | | | -48%; -83% | | | | |  |
| **Admission (Reference 71,8%)** | | | | | | | | | | | | | | | | | | | | | | | | | | | | | | | | | | | | | | | | | | | | | | | | | | | | | | | | | | | | | | | | | | | | | | | | | | | | | | | | | | | | | | | | | | | | | | | | | | | | | | | | | | | | | |  |  |
|  | | | | | 5% | | | | | | 10% | | | | | | 15% | | | | | | 20% | | | | | | 25% | | | | | | 30% | | | | | | 35% | | | | | | 40% | | | | | | 45% | | | | | | 50% | | | | | | 55% | | | | | 60% | | | | | 65% | | | | | 70% | | | | | 75% | | | | | 80% | | | | | 85% | | | | | 90% | | | | | 95% | | | | |  |  |
| BC | | | | | -52%; -89% | | | | | | -46%; -79% | | | | | | -40%; -69% | | | | | | -35%; -60% | | | | | | -30%; -52% | | | | | | -26%; -45% | | | | | | -23%; -40% | | | | | | -20%; -34% | | | | | | -15%; -26% | | | | | | -10%; -18% | | | | | | -6%; -10% | | | | | -1%; -2% | | | | | 4%; 7% | | | | | 7%; 12% | | | | | 8%; 13% | | | | | 8%; 13% | | | | | 8%; 13% | | | | | 8%; 13% | | | | | 8%; 13% | | | | |  |  |
| MB | | | | | -52%; -89% | | | | | | -46%; -79% | | | | | | -40%; -69% | | | | | | -35%; -60% | | | | | | -30%; -52% | | | | | | -26%; -45% | | | | | | -23%; -40% | | | | | | -20%; -34% | | | | | | -15%; -26% | | | | | | -10%; -18% | | | | | | -6%; -10% | | | | | -1%; -2% | | | | | 4%; 7% | | | | | 7%; 12% | | | | | 8%; 13% | | | | | 8%; 13% | | | | | 8%; 13% | | | | | 8%; 13% | | | | | 8%; 13% | | | | |  |  |
| NL | | | | | -47%; -81% | | | | | | -42%; -72% | | | | | | -36%; -62% | | | | | | -32%; -54% | | | | | | -27%; -47% | | | | | | -24%; -41% | | | | | | -21%; -36% | | | | | | -18%; -31% | | | | | | -14%; -23% | | | | | | -9%; -15% | | | | | | -4%; -7% | | | | | 0%; 1% | | | | | 5%; 9% | | | | | 9%; 15% | | | | | 9%; 15% | | | | | 9%; 15% | | | | | 9%; 15% | | | | | 9%; 15% | | | | | 9%; 15% | | | | |  |  |
| NS | | | | | -47%; -81% | | | | | | -42%; -72% | | | | | | -36%; -61% | | | | | | -31%; -54% | | | | | | -27%; -47% | | | | | | -24%; -41% | | | | | | -21%; -36% | | | | | | -18%; -31% | | | | | | -14%; -24% | | | | | | -9%; -16% | | | | | | -4%; -7% | | | | | 1%; 2% | | | | | 6%; 10% | | | | | 9%; 15% | | | | | 9%; 16% | | | | | 8%; 14% | | | | | 8%; 14% | | | | | 9%; 15% | | | | | 9%; 15% | | | | |  |  |
| ON | | | | | -54%; -94% | | | | | | -51%; -88% | | | | | | -54%; -93% | | | | | | -54%; -92% | | | | | | -53%; -92% | | | | | | -53%; -92% | | | | | | -54%; -93% | | | | | | -55%; -95% | | | | | | -55%; -95% | | | | | | -55%; -95% | | | | | | -55%; -95% | | | | | -55%; -95% | | | | | -55%; -95% | | | | | -55%; -95% | | | | | -55%; -95% | | | | | -55%; -95% | | | | | -55%; -95% | | | | | -55%; -95% | | | | | -55%; -95% | | | | |  |  |
| PEI | | | | | -47%; -81% | | | | | | -41%; -71% | | | | | | -36%; -62% | | | | | | -31%; -53% | | | | | | -28%; -47% | | | | | | -24%; -41% | | | | | | -21%; -36% | | | | | | -18%; -31% | | | | | | -14%; -24% | | | | | | -9%; -15% | | | | | | -5%; -9% | | | | | 1%; 1% | | | | | 5%; 9% | | | | | 9%; 15% | | | | | 10%; 16% | | | | | 8%; 14% | | | | | 9%; 16% | | | | | 9%; 15% | | | | | 9%; 15% | | | | |  |  |
| QC | | | | | -17%; -29% | | | | | | -16%; -27% | | | | | | -13%; -22% | | | | | | -8%; -14% | | | | | | -5%; -8% | | | | | | -2%; -3% | | | | | | 0%; 0% | | | | | | -1%; -1% | | | | | | 0%; 0% | | | | | | 0%; 0% | | | | | | 0%; 0% | | | | | 0%; -1% | | | | | -1%; -1% | | | | | -2%; -3% | | | | | -2%; -3% | | | | | -2%; -3% | | | | | -2%; -3% | | | | | -2%; -3% | | | | | -2%; -4% | | | | |  |  |
| **Patient from mental or mother and children department (Reference 13.2%)** | | | | | | | | | | | | | | | | | | | | | | | | | | | | | | | | | | | | | | | | | | | | | | | | | | | | | | | | | | | | | | | | | | | | | | | | | | | | | | | | | | | | | | | | | | | | | | | | | | | | | | | | | | | | |  |  |  |
|  |  | | | 5% | | | | | | 10% | | | | | | 15% | | | | | | 20% | | | | | | 25% | | | | | | 30% | | | | | | 35% | | | | | | 40% | | | | | | 45% | | | | | | 50% | | | | | | 55% | | | | | 60% | | | | | 65% | | | | | 70% | | | | | 75% | | | | | 80% | | | | | 85% | | | | | 90% | | | | | 95% | | | | | | | |
|  | BC | | | 7%; 11% | | | | | | 8%; 13% | | | | | | 9%; 16% | | | | | | 10%; 17% | | | | | | 10%; 17% | | | | | | 9%; 15% | | | | | | 8%; 14% | | | | | | 7%; 12% | | | | | | 6%; 10% | | | | | | 6%; 10% | | | | | | 4%; 7% | | | | | 3%; 6% | | | | | 3%; 5% | | | | | 1%; 2% | | | | | 3%; 6% | | | | | 5%; 9% | | | | | 6%; 10% | | | | | 8%; 13% | | | | | 8%; 14% | | | | | | | |
|  | MB | | | 6%; 10% | | | | | | 7%; 12% | | | | | | 8%; 13% | | | | | | 8%; 14% | | | | | | 8%; 13% | | | | | | 8%; 13% | | | | | | 7%; 11% | | | | | | 6%; 10% | | | | | | 5%; 8% | | | | | | 3%; 6% | | | | | | 2%; 4% | | | | | 1%; 2% | | | | | 1%; 2% | | | | | 0%; 1% | | | | | 1%; 2% | | | | | 3%; 5% | | | | | 4%; 7% | | | | | 6%; 9% | | | | | 6%; 10% | | | | | | | |
|  | NL | | | 5%; 9% | | | | | | 1%; 2% | | | | | | -3%; -6% | | | | | | -7%; -12% | | | | | | -11%; -20% | | | | | | -16%; -27% | | | | | | -18%; -32% | | | | | | -22%; -37% | | | | | | -25%; -43% | | | | | | -27%; -47% | | | | | | -30%; -52% | | | | | -33%; -56% | | | | | -36%; -61% | | | | | -38%; -65% | | | | | -41%; -70% | | | | | -44%; -76% | | | | | -48%; -82% | | | | | -51%; -88% | | | | | -53%; -90% | | | | | | | |
|  | NS | | | -55%; -95% | | | | | | -55%; -95% | | | | | | -55%; -95% | | | | | | -55%; -95% | | | | | | -55%; -95% | | | | | | -55%; -95% | | | | | | -55%; -95% | | | | | | -55%; -95% | | | | | | -55%; -95% | | | | | | -55%; -95% | | | | | | -55%; -95% | | | | | -56%; -96% | | | | | -55%; -95% | | | | | -55%; -95% | | | | | -56%; -96% | | | | | -55%; -95% | | | | | -55%; -95% | | | | | -55%; -95% | | | | | -55%; -95% | | | | | | | |
|  | ON | | | 5%; 9% | | | | | | 7%; 12% | | | | | | 8%; 13% | | | | | | 8%; 14% | | | | | | 8%; 14% | | | | | | 8%; 13% | | | | | | 7%; 11% | | | | | | 6%; 10% | | | | | | 5%; 8% | | | | | | 3%; 6% | | | | | | 2%; 4% | | | | | 2%; 3% | | | | | 1%; 2% | | | | | 1%; 1% | | | | | 1%; 2% | | | | | 3%; 4% | | | | | 4%; 7% | | | | | 6%; 9% | | | | | 6%; 10% | | | | | | | |
|  | PEI | | | 8%; 13% | | | | | | 8%; 14% | | | | | | 9%; 16% | | | | | | 10%; 17% | | | | | | 10%; 17% | | | | | | 9%; 15% | | | | | | 8%; 14% | | | | | | 7%; 12% | | | | | | 7%; 11% | | | | | | 5%; 8% | | | | | | 4%; 6% | | | | | 3%; 6% | | | | | 2%; 4% | | | | | 3%; 4% | | | | | 3%; 5% | | | | | 3%; 4% | | | | | 6%; 10% | | | | | 7%; 13% | | | | | 8%; 14% | | | | | | | |
|  | QC | | | 7%; 12% | | | | | | 8%; 14% | | | | | | 9%; 16% | | | | | | 10%; 16% | | | | | | 10%; 17% | | | | | | 9%; 15% | | | | | | 8%; 14% | | | | | | 7%; 13% | | | | | | 6%; 10% | | | | | | 5%; 8% | | | | | | 4%; 6% | | | | | 3%; 6% | | | | | 3%; 5% | | | | | 2%; 4% | | | | | 3%; 6% | | | | | 4%; 7% | | | | | 6%; 10% | | | | | 7%; 12% | | | | | 8%; 13% | | | | | | | |
|  | **Previous admission within the last 7 or up to 90 days (Reference 66.0%)** | | | | | | | | | | | | | | | | | | | | | | | | | | | | | | | | | | | | | | | | | | | | | | | | | | | | | | | | | | | | | | | | | | | | | | | | | | | | | | | | | | | | | | | | | | | | | | | | | | | | | | | | | | |  |  |  |  |
|  |  | | 5% | | | | | | 10% | | | | | | 15% | | | | | | 20% | | | | | | 25% | | | | | | 30% | | | | | | 35% | | | | | | 40% | | | | | | 45% | | | | | | 50% | | | | | | 55% | | | | | 60% | | | | | 65% | | | | | 70% | | | | | 75% | | | | | 80% | | | | | 85% | | | | | 90% | | | | | 95% | | | | |  |  |  |  |
|  | BC | | -24%; -41% | | | | | | -23%; -39% | | | | | | -22%; -38% | | | | | | -21%; -36% | | | | | | -20%; -35% | | | | | | -17%; -30% | | | | | | -14%; -25% | | | | | | -13%; -22% | | | | | | -9%; -15% | | | | | | -5%; -8% | | | | | | 0%; 0% | | | | | 4%; 7% | | | | | 8%; 13% | | | | | 11%; 20% | | | | | 12%; 20% | | | | | 11%; 19% | | | | | 11%; 19% | | | | | 12%; 20% | | | | | 12%; 20% | | | | |  |  |  |  |
|  | MB | | -25%; -43% | | | | | | -24%; -42% | | | | | | -23%; -40% | | | | | | -22%; -38% | | | | | | -21%; -36% | | | | | | -19%; -33% | | | | | | -17%; -29% | | | | | | -14%; -23% | | | | | | -10%; -17% | | | | | | -6%; -11% | | | | | | -2%; -3% | | | | | 2%; 4% | | | | | 7%; 12% | | | | | 10%; 17% | | | | | 10%; 17% | | | | | 10%; 17% | | | | | 10%; 18% | | | | | 10%; 18% | | | | | 10%; 18% | | | | |  |  |  |  |
|  | NL | | -27%; -46% | | | | | | -27%; -46% | | | | | | -27%; -46% | | | | | | -27%; -46% | | | | | | -26%; -45% | | | | | | -26%; -44% | | | | | | -24%; -41% | | | | | | -21%; -37% | | | | | | -18%; -31% | | | | | | -15%; -25% | | | | | | -11%; -19% | | | | | -6%; -11% | | | | | -3%; -5% | | | | | 3%; 5% | | | | | 8%; 13% | | | | | 12%; 21% | | | | | 16%; 28% | | | | | 21%; 36% | | | | | 25%; 43% | | | | |  |  |  |  |
|  | NS | | -27%; -47% | | | | | | -31%; -54% | | | | | | -35%; -61% | | | | | | -39%; -68% | | | | | | -43%; -74% | | | | | | -46%; -80% | | | | | | -49%; -84% | | | | | | -51%; -87% | | | | | | -52%; -90% | | | | | | -53%; -91% | | | | | | -54%; -93% | | | | | -55%; -94% | | | | | -55%; -95% | | | | | -55%; -95% | | | | | -55%; -95% | | | | | -55%; -95% | | | | | -55%; -95% | | | | | -55%; -95% | | | | | -55%; -95% | | | | |  |  |  |  |
|  | ON | | -25%; -43% | | | | | | -24%; -42% | | | | | | -23%; -40% | | | | | | -22%; -39% | | | | | | -21%; -36% | | | | | | -19%; -33% | | | | | | -17%; -29% | | | | | | -14%; -23% | | | | | | -10%; -18% | | | | | | -6%; -10% | | | | | | -2%; -3% | | | | | 2%; 4% | | | | | 6%; 11% | | | | | 10%; 17% | | | | | 10%; 18% | | | | | 10%; 18% | | | | | 10%; 17% | | | | | 10%; 18% | | | | | 10%; 17% | | | | |  |  |  |  |
|  | PEI | | -23%; -40% | | | | | | -22%; -39% | | | | | | -22%; -38% | | | | | | -21%; -35% | | | | | | -19%; -33% | | | | | | -18%; -31% | | | | | | -14%; -24% | | | | | | -12%; -21% | | | | | | -9%; -16% | | | | | | -5%; -8% | | | | | | 0%; 0% | | | | | 4%; 7% | | | | | 8%; 14% | | | | | 11%; 19% | | | | | 11%; 19% | | | | | 12%; 20% | | | | | 11%; 19% | | | | | 12%; 20% | | | | | 12%; 21% | | | | |  |  |  |  |
|  | QC | | -24%; -40% | | | | | | -23%; -40% | | | | | | -22%; -38% | | | | | | -21%; -36% | | | | | | -19%; -33% | | | | | | -17%; -30% | | | | | | -15%; -26% | | | | | | -12%; -21% | | | | | | -9%; -15% | | | | | | -5%; -8% | | | | | | -1%; -1% | | | | | 4%; 6% | | | | | 8%; 13% | | | | | 11%; 19% | | | | | 12%; 20% | | | | | 12%; 20% | | | | | 12%; 20% | | | | | 12%; 20% | | | | | 12%; 20% | | | | |  |  |  |  |
|  | **Previous admission within the last 3 days (Reference 3.0%)** | | | | | | | | | | | | | | | | | | | | | | | | | | | | | | | | | | | | | | | | | | | | | | | | | | | | | | | | | | | | | | | | | | | | | | | | | | | | | | | | | | | | | | | | | | | | | | | | | | | | | | | | | |  |  |  |  |  |
|  |  | 5% | | | | | | 10% | | | | | | 15% | | | | | | 20% | | | | | | 25% | | | | | | 30% | | | | | | 35% | | | | | | 40% | | | | | | 45% | | | | | | 50% | | | | | | 55% | | | | | 60% | | | | | 65% | | | | | 70% | | | | | 75% | | | | | 80% | | | | | 85% | | | | | 90% | | | | | 95% | | | | |  |  |  |  |  |
|  | BC | 9%; 15% | | | | | | 9%; 15% | | | | | | 10%; 17% | | | | | | 9%; 16% | | | | | | 9%; 16% | | | | | | 9%; 16% | | | | | | 9%; 15% | | | | | | 10%; 16% | | | | | | 10%; 18% | | | | | | 9%; 16% | | | | | | 10%; 18% | | | | | 10%; 18% | | | | | 11%; 18% | | | | | 14%; 24% | | | | | 17%; 30% | | | | | 17%; 29% | | | | | 16%; 28% | | | | | 15%; 25% | | | | | 12%; 21% | | | | |  |  |  |  |  |
|  | MB | 8%; 13% | | | | | | 8%; 13% | | | | | | 8%; 14% | | | | | | 8%; 14% | | | | | | 8%; 14% | | | | | | 8%; 14% | | | | | | 8%; 14% | | | | | | 9%; 15% | | | | | | 9%; 15% | | | | | | 9%; 15% | | | | | | 9%; 15% | | | | | 9%; 15% | | | | | 9%; 15% | | | | | 13%; 22% | | | | | 15%; 27% | | | | | 15%; 27% | | | | | 16%; 27% | | | | | 14%; 24% | | | | | 11%; 19% | | | | |  |  |  |  |  |
|  | NL | -2%; -3% | | | | | | -2%; -3% | | | | | | -2%; -3% | | | | | | -1%; -2% | | | | | | -1%; -2% | | | | | | -2%; -3% | | | | | | -1%; -2% | | | | | | -1%; -2% | | | | | | -1%; -2% | | | | | | -1%; -1% | | | | | | 0%; 0% | | | | | 0%; -1% | | | | | 0%; -1% | | | | | 6%; 9% | | | | | 12%; 21% | | | | | 17%; 30% | | | | | 22%; 37% | | | | | 24%; 42% | | | | | 25%; 44% | | | | |  |  |  |  |  |
|  | NS | -53%; -92% | | | | | | -48%; -83% | | | | | | -44%; -75% | | | | | | -39%; -66% | | | | | | -34%; -58% | | | | | | -29%; -49% | | | | | | -24%; -41% | | | | | | -19%; -32% | | | | | | -14%; -23% | | | | | | -8%; -14% | | | | | | -3%; -6% | | | | | 1%; 2% | | | | | 6%; 10% | | | | | 11%; 19% | | | | | 15%; 25% | | | | | 15%; 25% | | | | | 15%; 26% | | | | | 14%; 23% | | | | | 11%; 18% | | | | |  |  |  |  |  |
|  | ON | 8%; 13% | | | | | | 8%; 13% | | | | | | 7%; 13% | | | | | | 8%; 14% | | | | | | 8%; 14% | | | | | | 8%; 14% | | | | | | 8%; 14% | | | | | | 9%; 15% | | | | | | 9%; 15% | | | | | | 9%; 15% | | | | | | 9%; 15% | | | | | 9%; 15% | | | | | 9%; 16% | | | | | 13%; 22% | | | | | 16%; 27% | | | | | 15%; 26% | | | | | 15%; 26% | | | | | 14%; 23% | | | | | 11%; 18% | | | | |  |  |  |  |  |
|  | PEI | 9%; 16% | | | | | | 9%; 15% | | | | | | 10%; 17% | | | | | | 9%; 16% | | | | | | 9%; 15% | | | | | | 9%; 15% | | | | | | 10%; 17% | | | | | | 9%; 16% | | | | | | 9%; 15% | | | | | | 10%; 17% | | | | | | 11%; 18% | | | | | 11%; 18% | | | | | 10%; 18% | | | | | 15%; 25% | | | | | 17%; 29% | | | | | 17%; 29% | | | | | 17%; 29% | | | | | 16%; 27% | | | | | 12%; 20% | | | | |  |  |  |  |  |
|  | QC | 9%; 16% | | | | | | 9%; 15% | | | | | | 9%; 16% | | | | | | 9%; 15% | | | | | | 9%; 16% | | | | | | 10%; 17% | | | | | | 10%; 17% | | | | | | 10%; 17% | | | | | | 10%; 17% | | | | | | 10%; 18% | | | | | | 10%; 18% | | | | | 10%; 18% | | | | | 10%; 18% | | | | | 15%; 25% | | | | | 17%; 29% | | | | | 17%; 29% | | | | | 17%; 29% | | | | | 15%; 26% | | | | | 12%; 21% | | | | |  |  |  |  |  |
|  | Absolute difference (province – CNISP); Relative difference (province – CNISP)/CNISP | | | | | | | | | | | | | | | | | | | | | | | | | | | | | | |  |  |  |  |  |  |  |  |  |  |  |  |  |  |  |  |  |  |  |  |  |  |  |  |  |  |  |  |  |  |  |  |  |  |  |  |  |  |  |  |  |  |  |  |  |  |  |  |  |  |  |  |  |  |  |  |  |  |  |  |  |  |  |  |  |  |  |  |  |  |  |  |  |  |  |  |  |  |  |  |
|  | Comparable | | | | | | | | | | | | | | | | | | | | | | | | | | | | | | |  |  |  |  |  |  |  |  |  |  |  |  |  |  |  |  |  |  |  |  |  |  |  |  |  |  |  |  |  |  |  |  |  |  |  |  |  |  |  |  |  |  |  |  |  |  |  |  |  |  |  |  |  |  |  |  |  |  |  |  |  |  |  |  |  |  |  |  |  |  |  |  |  |  |  |  |  |  |  |  |
|  | Province not comparable with CNISP | | | | | | | | | | | | | | | | | | | | | | | | | | | | | | |  |  |  |  |  |  |  |  |  |  |  |  |  |  |  |  |  |  |  |  |  |  |  |  |  |  |  |  |  |  |  |  |  |  |  |  |  |  |  |  |  |  |  |  |  |  |  |  |  |  |  |  |  |  |  |  |  |  |  |  |  |  |  |  |  |  |  |  |  |  |  |  |  |  |  |  |  |  |  |  |

**Table S9-** Distribution of absolute and relative differences of proportions of patients meeting case definition: provinces vs CNISP with both varying proportions of elements, by province MRSA

| **Three days of admission (Reference 34.0%)** \| \| \| \| \| \| \| \| \| \| \| \| \| \| \| \| \| \| \|; \| 5% \| 10% \| 15% \| 20% \| 25% \| 30% \| 35% \| 40% \| 45% \| 50% \| 55% \| 60% \| 65% \| 70% \| 75% \| 80% \| 85% \| 90% \| 95%; BC \| 0%; 0% \| 0%; 0% \| 0%; 0% \| 0%; 0% \| 0%; 0% \| 0%; 0% \| 0%; 0% \| 0%; 0% \| 0%; 0% \| 0%; 0% \| 0%; 0% \| 0%; 0% \| 0%; 0% \| 0%; 0% \| 0%; 0% \| 0%; 0% \| 0%; 0% \| 0%; 0% \| 0%; 0%; MB \| 0%; 0% \| 0%; 0% \| 0%; 0% \| 0%; 0% \| 0%; 0% \| 0%; 0% \| 0%; 0% \| 0%; 0% \| 0%; 1% \| 0%; 0% \| 0%; 0% \| 0%; 0% \| 0%; 0% \| 0%; 0% \| 0%; 0% \| 0%; 0% \| 0%; 0% \| 0%; 0% \| 0%; 0%; NL \| 0%; 0% \| 0%; 0% \| 0%; 0% \| 1%; 1% \| 1%; 1% \| 1%; 2% \| 2%; 2% \| 2%; 2% \| 2%; 3% \| 2%; 3% \| 2%; 4% \| 3%; 5% \| 3%; 5% \| 4%; 6% \| 5%; 6% \| 5%; 7% \| 6%; 7% \| 7%; 8% \| 7%; 8%; NS \| 0%; 0% \| 1%; 2% \| 1%; 1% \| 0%; 1% \| 1%; 1% \| 2%; 2% \| 2%; 2% \| 2%; 2% \| 2%; 3% \| 1%; 2% \| 2%; 4% \| 3%; 4% \| 4%; 6% \| 4%; 6% \| 4%; 6% \| 6%; 8% \| 6%; 7% \| 6%; 8% \| 7%; 8%; ON \| -63%; -96% \| -63%; -96% \| -63%; -96% \| -63%; -96% \| -63%; -96% \| -63%; -96% \| -63%; -95% \| -63%; -95% \| -63%; -95% \| -63%; -94% \| -63%; -94% \| -63%; -93% \| -63%; -92% \| -63%; -90% \| -65%; -89% \| -68%; -90% \| -72%; -92% \| -77%; -93% \| -81%; -94%; PEI \| -1%; -1% \| 0%; 0% \| 1%; 1% \| 1%; 1% \| 1%; 1% \| 1%; 1% \| 1%; 2% \| 2%; 2% \| 2%; 3% \| 3%; 4% \| 2%; 3% \| 3%; 5% \| 4%; 6% \| 4%; 6% \| 4%; 6% \| 6%; 7% \| 6%; 7% \| 7%; 8% \| 7%; 9%; QC \| -9%; -14% \| -9%; -14% \| -9%; -14% \| -9%; -14% \| -10%; -14% \| -9%; -14% \| -9%; -14% \| -9%; -14% \| -10%; -15% \| -9%; -14% \| -9%; -14% \| -9%; -13% \| -8%; -12% \| -7%; -9% \| -7%; -9% \| -8%; -10% \| -9%; -12% \| -11%; -13% \| -11%; -13% | | | | | | | | | | | | | | | | | | | | | | | | | | | | | | | | | | | | | | | | | | | | | | | | | | | | | | | | | | | | | | | | | | | | | | | | | | | | | | | | | |  |  |  |
| --- | --- | --- | --- | --- | --- | --- | --- | --- | --- | --- | --- | --- | --- | --- | --- | --- | --- | --- | --- | --- | --- | --- | --- | --- | --- | --- | --- | --- | --- | --- | --- | --- | --- | --- | --- | --- | --- | --- | --- | --- | --- | --- | --- | --- | --- | --- | --- | --- | --- | --- | --- | --- | --- | --- | --- | --- | --- | --- | --- | --- | --- | --- | --- | --- | --- | --- | --- | --- | --- | --- | --- | --- | --- | --- | --- | --- | --- | --- | --- | --- | --- | --- | --- | --- |
| **Mother status (Reference 5.5%)** \| \| \| \| \| \| \| \| \| \| \| \| \| \| \| \| \| \| \|; \| 5% \| 10% \| 15% \| 20% \| 25% \| 30% \| 35% \| 40% \| 45% \| 50% \| 55% \| 60% \| 65% \| 70% \| 75% \| 80% \| 85% \| 90% \| 95%; BC \| 0%; 0% \| 0%; 0% \| 0%; 0% \| 0%; 0% \| 0%; 0% \| 0%; 0% \| 0%; 0% \| 0%; 0% \| 0%; 0% \| 0%; 0% \| 0%; 0% \| 0%; 0% \| 0%; 0% \| 0%; 0% \| 0%; 0% \| 0%; 0% \| 0%; 0% \| 0%; 0% \| 0%; 0%; MB \| 0%; 0% \| 0%; 0% \| 0%; 0% \| 0%; 0% \| 0%; 0% \| 0%; 0% \| 0%; 0% \| 0%; 0% \| 0%; 0% \| 0%; 0% \| 0%; 0% \| 0%; 0% \| 0%; 0% \| 1%; 1% \| 0%; 0% \| 0%; 0% \| 0%; 2% \| 0%; 1% \| 0%; 0%; NL \| 1%; 2% \| 2%; 3% \| 2%; 3% \| 3%; 4% \| 2%; 3% \| 2%; 3% \| 3%; 4% \| 5%; 8% \| 6%; 11% \| 8%; 14% \| 9%; 19% \| 10%; 22% \| 11%; 28% \| 13%; 36% \| 15%; 50% \| 17%; 69% \| 19%; 102% \| 21%; 167% \| 24%; 336%; NS \| 2%; 3% \| 2%; 4% \| 2%; 3% \| 2%; 3% \| 3%; 4% \| 3%; 4% \| 2%; 2% \| 5%; 8% \| 6%; 10% \| 7%; 13% \| 10%; 20% \| 11%; 25% \| 12%; 30% \| 13%; 37% \| 15%; 50% \| 18%; 74% \| 18%; 98% \| 20%; 160% \| 23%; 334%; ON \| -63%; -95% \| -61%; -96% \| -59%; -96% \| -59%; -95% \| -60%; -95% \| -62%; -95% \| -61%; -95% \| -57%; -96% \| -54%; -95% \| -50%; -94% \| -46%; -93% \| -41%; -91% \| -36%; -89% \| -30%; -86% \| -24%; -82% \| -19%; -77% \| -12%; -67% \| -6%; -49% \| 0%; -3%; PEI \| 1%; 2% \| 2%; 3% \| 2%; 4% \| 4%; 6% \| 3%; 4% \| 2%; 4% \| 3%; 5% \| 5%; 8% \| 6%; 11% \| 7%; 13% \| 10%; 19% \| 10%; 23% \| 12%; 29% \| 13%; 38% \| 15%; 51% \| 17%; 71% \| 18%; 100% \| 21%; 165% \| 25%; 356%; QC \| -9%; -14% \| -5%; -8% \| -1%; -1% \| 2%; 4% \| 4%; 6% \| 7%; 11% \| 9%; 15% \| 13%; 21% \| 13%; 24% \| 14%; 26% \| 15%; 31% \| 16%; 35% \| 17%; 41% \| 18%; 52% \| 17%; 59% \| 14%; 59% \| 11%; 60% \| 8%; 63% \| 3%; 44% | | | | | | | | | | | | | | | | | | | | | | | | | | | | | | | | | | | | | | | | | | | | | | | | | | | | | | | | | | | | | | | | | | | | | | | | | | | | | | | | | | | |  |
| **Admission (Reference 71,8%)** | | | | | | | | | | | | | | | | | | | | | | | | | | | | | | | | | | | | | | | | | | | | | | | | | | | | | | | | | | | | | | | | | | | | | | | | | | | | | | | | | | | |  |
|  | | 5% | | | | 10% | | | | 15% | | | | 20% | | | | 25% | | | | 30% | | | | | 35% | | | | 40% | | | | 45% | | | | 50% | | | | 55% | | | | 60% | | | | 65% | | | | 70% | | | | 75% | | | | 80% | | | | 85% | | | | 90% | | | | 95% | | | |  |  |  |  |  |  |
| BC | | 0%; -2% | | | | 0%; 1% | | | | 0%; 0% | | | | 0%; 0% | | | | 0%; 0% | | | | 0%; 0% | | | | | 0%; 0% | | | | 0%; 0% | | | | 0%; 0% | | | | 0%; 0% | | | | 0%; 0% | | | | 0%; 0% | | | | 0%; 0% | | | | 0%; 0% | | | | 0%; 0% | | | | 0%; 0% | | | | 0%; 0% | | | | 0%; 0% | | | | 0%; 0% | | | |  |  |  |  |  |  |
| MB | | 0%; 0% | | | | 0%; 1% | | | | 0%; 1% | | | | 0%; -1% | | | | 0%; -1% | | | | 0%; -1% | | | | | 0%; 0% | | | | 0%; 0% | | | | 0%; 0% | | | | 0%; 0% | | | | 0%; -1% | | | | 0%; 0% | | | | 0%; 0% | | | | 0%; 0% | | | | 0%; 0% | | | | 0%; 0% | | | | 0%; 0% | | | | 0%; 0% | | | | 0%; 0% | | | |  |  |  |  |  |  |
| NL | | 4%; 69% | | | | 4%; 35% | | | | 4%; 21% | | | | 3%; 13% | | | | 3%; 10% | | | | 2%; 7% | | | | | 2%; 6% | | | | 2%; 4% | | | | 2%; 3% | | | | 1%; 3% | | | | 1%; 2% | | | | 1%; 2% | | | | 1%; 2% | | | | 2%; 2% | | | | 1%; 2% | | | | 1%; 2% | | | | 1%; 2% | | | | 1%; 2% | | | | 1%; 2% | | | |  |  |  |  |  |  |
| NS | | 5%; 73% | | | | 4%; 36% | | | | 4%; 24% | | | | 3%; 14% | | | | 3%; 10% | | | | 3%; 8% | | | | | 2%; 6% | | | | 2%; 5% | | | | 1%; 3% | | | | 1%; 2% | | | | 2%; 3% | | | | 2%; 4% | | | | 2%; 4% | | | | 2%; 3% | | | | 2%; 2% | | | | 0%; 1% | | | | 1%; 1% | | | | 1%; 2% | | | | 1%; 2% | | | |  |  |  |  |  |  |
| ON | | -3%; -42% | | | | -5%; -45% | | | | -14%; -78% | | | | -19%; -80% | | | | 23%; -82% | | | | -27%; -85% | | | | | -31%; -88% | | | | -35%; -93% | | | | -40%; -93% | | | | -45%; -94% | | | | -50%; -94% | | | | -54%; -95% | | | | -59%; -95% | | | | -62%; -96% | | | | -63%; -95% | | | | -63%; -96% | | | | -63%; -96% | | | | -63%; -96% | | | | -63%; -95% | | | |  |  |  |  |  |  |
| PEI | | 5%; 73% | | | | 5%; 38% | | | | 4%; 23% | | | | 4%; 15% | | | | 3%; 10% | | | | 2%; 7% | | | | | 2%; 7% | | | | 2%; 5% | | | | 1%; 3% | | | | 2%; 3% | | | | 1%; 1% | | | | 2%; 3% | | | | 2%; 3% | | | | 2%; 3% | | | | 2%; 3% | | | | 1%; 1% | | | | 2%; 2% | | | | 1%; 1% | | | | 1%; 2% | | | |  |  |  |  |  |  |
| QC | | 35%; 542% | | | | 30%; 250% | | | | 27%; 150% | | | | 26%; 112% | | | | 26%; 91% | | | | 24%; 76% | | | | | 23%; 66% | | | | 19%; 50% | | | | 15%; 35% | | | | 10%; 22% | | | | 5%; 10% | | | | 1%; 1% | | | | -4%; -7% | | | | -9%; -14% | | | | -10%; -14% | | | | -10%; -14% | | | | -9%; -14% | | | | -9%; -14% | | | | -10%; -15% | | | |  |  |  |  |  |  |
| **Patient from mental or mother and children department (Reference 13.2%)** | | | | | | | | | | | | | | | | | | | | | | | | | | | | | | | | | | | | | | | | | | | | | | | | | | | | | | | | | | | | | | | | | | | | | | | | | | | | | | | | | | |  |  |
|  | | | 5% | | | | 10% | | | | 15% | | | | 20% | | | | 25% | | | | | 30% | | | | 35% | | | | 40% | | | | 45% | | | | 50% | | | | 55% | | | | 60% | | | | 65% | | | | 70% | | | | 75% | | | | 80% | | | | 85% | | | | 90% | | | | 95% | | | |  |  |  |  |  |
| BC | | | 1%; 2% | | | | 1%; 1% | | | | 1%; 2% | | | | 2%; 3% | | | | 2%; 3% | | | | | 1%; 2% | | | | 2%; 2% | | | | 2%; 3% | | | | 2%; 2% | | | | 3%; 4% | | | | 2%; 4% | | | | 2%; 3% | | | | 2%; 3% | | | | 1%; 2% | | | | 2%; 4% | | | | 3%; 4% | | | | 2%; 2% | | | | 2%; 3% | | | | 2%; 4% | | | |  |  |  |  |  |
| MB | | | 0%; 0% | | | | 0%; 0% | | | | 0%; 0% | | | | 0%; 0% | | | | 0%; 0% | | | | | 0%; 0% | | | | 0%; 0% | | | | 0%; 0% | | | | 0%; 0% | | | | 0%; 0% | | | | 0%; 0% | | | | 0%; 0% | | | | 0%; 0% | | | | 0%; 0% | | | | 0%; 0% | | | | 0%; 0% | | | | 0%; 0% | | | | 0%; 0% | | | | 0%; 0% | | | |  |  |  |  |  |
| NL | | | -1%; -1% | | | | -6%; -9% | | | | -11%; -17% | | | | -15%; -23% | | | | -19%; -29% | | | | | -23%; -35% | | | | -25%; -39% | | | | -27%; -43% | | | | -29%; -47% | | | | -30%; -50% | | | | -32%; -53% | | | | -34%; -57% | | | | -37%; -62% | | | | -38%; -65% | | | | -42%; -71% | | | | -47%; -77% | | | | -52%; -83% | | | | -57%; -89% | | | | -59%; -91% | | | |  |  |  |  |  |
| NS | | | -61%; -96% | | | | -62%; -96% | | | | -63%; -96% | | | | -6%; -95% | | | | -63%; -95% | | | | | -63%; -95% | | | | -62%; -96% | | | | -61%; -95% | | | | -60%; -95% | | | | -59%; -95% | | | | -58%; -96% | | | | -57%; -96% | | | | -57%; -95% | | | | -56%; -95% | | | | -57%; -96% | | | | -58%; -96% | | | | -60%; -96% | | | | -61%; -96% | | | | -61%; -96% | | | |  |  |  |  |  |
| ON | | | 0%; 0% | | | | 0%; 0% | | | | 0%; 0% | | | | 0%; 0% | | | | 0%; 0% | | | | | 0%; 0% | | | | 0%; 0% | | | | 0%; 0% | | | | 0%; 1% | | | | 0%; 0% | | | | 0%; 0% | | | | 0%; 0% | | | | 0%; 0% | | | | 0%; 1% | | | | 0%; 0% | | | | 0%; 0% | | | | 0%; 0% | | | | 0%; 0% | | | | 0%; 0% | | | |  |  |  |  |  |
| PEI | | | 2%; 4% | | | | 1%; 2% | | | | 1%; 2% | | | | 2%; 3% | | | | 2%; 3% | | | | | 2%; 2% | | | | 1%; 2% | | | | 2%; 3% | | | | 2%; 4% | | | | 1%; 2% | | | | 2%; 2% | | | | 2%; 3% | | | | 1%; 2% | | | | 2%; 3% | | | | 2%; 3% | | | | 0%; 0% | | | | 2%; 3% | | | | 2%; 3% | | | | 2%; 4% | | | |  |  |  |  |  |
| QC | | | 1%; 2% | | | | 1%; 2% | | | | 1%; 2% | | | | 2%; 2% | | | | 2%; 3% | | | | | 2%; 2% | | | | 2%; 3% | | | | 2%; 3% | | | | 2%; 3% | | | | 2%; 3% | | | | 2%; 3% | | | | 2%; 3% | | | | 2%; 3% | | | | 2%; 3% | | | | 2%; 3% | | | | 2%; 3% | | | | 2%; 3% | | | | 2%; 3% | | | | 2%; 2% | | | |  |  |  |  |  |
| **Previous admission within the last 7 or up to 90 days (Reference 66.0%)** | | | | | | | | | | | | | | | | | | | | | | | | | | | | | | | | | | | | | | | | | | | | | | | | | | | | | | | | | | | | | | | | | | | | | | | | | | | | | | | | | |  |  |  |
|  | | | | | 5% | | | 10% | | | | 15% | | | | 20% | | | | 25% | | | | | 30% | | | | 35% | | | | 40% | | | | 45% | | | | 50% | | | | 55% | | | | 60% | | | | 65% | | | | 70% | | | | 75% | | | | 80% | | | | 85% | | | | 90% | | | | 95% | | | | |  |  |  |
| BC | | | | | 1%; 3% | | | 2%; 5% | | | | 1%; 3% | | | | 2%; 4% | | | | 1%; 2% | | | | | 2%; 4% | | | | 2%; 6% | | | | 1%; 2% | | | | 1%; 2% | | | | 1%; 3% | | | | 2%; 3% | | | | 2%; 3% | | | | 1%; 2% | | | | 2%; 3% | | | | 1%; 2% | | | | 1%; 1% | | | | 1%; 1% | | | | 1%; 2% | | | | 2%; 3% | | | | |  |  |  |
| MB | | | | | 0%; 0% | | | 0%; 0% | | | | 0%; 0% | | | | 0%; 0% | | | | 0%; 0% | | | | | 0%; 0% | | | | 0%; 0% | | | | 0%; 0% | | | | 0%; 0% | | | | 0%; 0% | | | | 0%; 0% | | | | 0%; 0% | | | | 0%; 0% | | | | 0%; 0% | | | | 0%; 0% | | | | 0%; 0% | | | | 0%; 0% | | | | 0%; 0% | | | | 0%; 0% | | | | |  |  |  |
| NL | | | | | -1%; -4% | | | -2%; -7% | | | | -4%; -10% | | | | -4%; -12% | | | | -5%; -14% | | | | | -6%; -16% | | | | -7%; -17% | | | | -8%; -17% | | | | -8%; -17% | | | | -9%; -16% | | | | -9%; -16% | | | | -9%; -14% | | | | -9%; -14% | | | | -7%; -10% | | | | -3%; -4% | | | | 2%; 3% | | | | 6%; 9% | | | | 11%; 16% | | | | 15%; 22% | | | | |  |  |  |
| NS | | | | | -2%; -6% | | | -7%; -21% | | | | -12%; -35% | | | | -17%; -48% | | | | -22%; -60% | | | | | -27%; -69% | | | | -32%; -77% | | | | -37%; -83% | | | | -42%; -88% | | | | -47%; -90% | | | | -52%; -93% | | | | -57%; -94% | | | | -62%; -95% | | | | -65%; -96% | | | | -66%; -96% | | | | -66%; -96% | | | | -66%; -96% | | | | -66%; -96% | | | | -65%; -96% | | | | |  |  |  |
| ON | | | | | 0%; 0% | | | 0%; 0% | | | | 0%; 0% | | | | 0%; 0% | | | | 0%; 0% | | | | | 0%; 0% | | | | 0%; 0% | | | | 0%; 0% | | | | 0%; -1% | | | | 0%; 0% | | | | 0%; 1% | | | | 0%; 0% | | | | 0%; 0% | | | | 0%; 0% | | | | 0%; 0% | | | | 0%; 0% | | | | 0%; 0% | | | | 0%; 0% | | | | 0%; 0% | | | | |  |  |  |
| PEI | | | | | 2%; 6% | | | 2%; 6% | | | | 1%; 4% | | | | 2%; 5% | | | | 2%; 5% | | | | | 1%; 3% | | | | 3%; 7% | | | | 1%; 3% | | | | 1%; 2% | | | | 1%; 3% | | | | 2%; 4% | | | | 2%; 3% | | | | 2%; 3% | | | | 1%; 2% | | | | 1%; 1% | | | | 2%; 2% | | | | 1%; 1% | | | | 2%; 2% | | | | 2%; 3% | | | | |  |  |  |
| QC | | | | | 2%; 5% | | | 1%; 4% | | | | 1%; 4% | | | | 2%; 4% | | | | 2%; 5% | | | | | 2%; 4% | | | | 2%; 4% | | | | 2%; 4% | | | | 2%; 3% | | | | 1%; 2% | | | | 1%; 2% | | | | 2%; 2% | | | | 1%; 2% | | | | 1%; 2% | | | | 1%; 2% | | | | 1%; 2% | | | | 1%; 2% | | | | 1%; 2% | | | | 1%; 2% | | | | |  |  |  |
| **Previous admission within the last 3 days (Reference 3.0%)** | | | | | | | | | | | | | | | | | | | | | | | | | | | | | | | | | | | | | | | | | | | | | | | | | | | | | | | | | | | | | | | | | | | | | | | | | | | | | | | | |  |  |  |  |
|  |  | | | 5% | | | | | 10% | | | | 15% | | | | 20% | | | | 25% | | | | | 30% | | | | 35% | | | | 40% | | | | 45% | | | | 50% | | | | 55% | | | | 60% | | | | 65% | | | | 70% | | | | 75% | | | | 80% | | | | 85% | | | | 90% | | | | 95% | | | | | | |
|  | BC | | | 1%; 2% | | | | | 1%; 2% | | | | 2%; 3% | | | | 1%; 2% | | | | 1%; 2% | | | | | 1%; 2% | | | | 1%; 1% | | | | 1%; 2% | | | | 2%; 3% | | | | 1%; 1% | | | | 1%; 2% | | | | 1%; 2% | | | | 2%; 2% | | | | 1%; 2% | | | | 2%; 3% | | | | 1%; 2% | | | | 1%; 1% | | | | 1%; 1% | | | | 2%; 2% | | | | | | |
|  | MB | | | 0%; 0% | | | | | 0%; 0% | | | | 0%; 0% | | | | 0%; 0% | | | | 0%; 0% | | | | | 0%; 0% | | | | 0%; 0% | | | | 0%; 0% | | | | 0%; 0% | | | | 0%; 0% | | | | 0%; 0% | | | | 0%; 0% | | | | 0%; 0% | | | | 0%; 0% | | | | 0%; 0% | | | | 0%; 0% | | | | 0%; 0% | | | | 0%; 0% | | | | 0%; 0% | | | | | | |
|  | NL | | | -9%; -14% | | | | | -9%; -14% | | | | -9%; -14% | | | | -9%; -14% | | | | -9%; -14% | | | | | -10%; -15% | | | | -9%; -14% | | | | -9%; -14% | | | | -10%; -14% | | | | -9%; -14% | | | | -9%; -13% | | | | -9%; -14% | | | | -9%; -14% | | | | -8%; -11% | | | | -3%; -4% | | | | 2%; 2% | | | | 6%; 8% | | | | 11%; 15% | | | | 15%; 22% | | | | | | |
|  | NS | | | -61%; -93% | | | | | -56%; -85% | | | | -51%; -78% | | | | -47%; -70% | | | | -42%; -63% | | | | | -37%; -56% | | | | -32%; -48% | | | | -27%; -41% | | | | -22%; -33% | | | | -17%; -26% | | | | -12%; -18% | | | | -8%; -11% | | | | -3%; -4% | | | | -2%; -3% | | | | -1%; -1% | | | | -1%; -1% | | | | 0%; -1% | | | | 0%; 0% | | | | 0%; 0% | | | | | | |
|  | ON | | | 0%; 0% | | | | | 0%; 0% | | | | 0%; -1% | | | | 0%; 0% | | | | 0%; 0% | | | | | 0%; 0% | | | | 0%; 0% | | | | 0%; 0% | | | | 0%; 0% | | | | 0%; 0% | | | | 0%; 0% | | | | 0%; 0% | | | | 0%; 0% | | | | 0%; 0% | | | | 0%; 0% | | | | 0%; 0% | | | | 0%; 0% | | | | 0%; 0% | | | | 0%; 0% | | | | | | |
|  | PEI | | | 2%; 3% | | | | | 1%; 2% | | | | 2%; 3% | | | | 2%; 2% | | | | 1%; 1% | | | | | 1%; 1% | | | | 2%; 2% | | | | 1%; 2% | | | | 0%; 0% | | | | 1%; 2% | | | | 2%; 3% | | | | 2%; 2% | | | | 1%; 2% | | | | 2%; 2% | | | | 1%; 2% | | | | 1%; 2% | | | | 2%; 2% | | | | 2%; 3% | | | | 1%; 2% | | | | | | |
|  | QC | | | 2%; 2% | | | | | 1%; 2% | | | | 1%; 2% | | | | 1%; 2% | | | | 1%; 2% | | | | | 2%; 2% | | | | 1%; 2% | | | | 2%; 2% | | | | 1%; 2% | | | | 2%; 2% | | | | 1%; 2% | | | | 1%; 2% | | | | 1%; 2% | | | | 2%; 2% | | | | 1%; 2% | | | | 2%; 2% | | | | 2%; 2% | | | | 2%; 2% | | | | 2%; 2% | | | | | | |
|  | Absolute difference (province – CNISP); Relative difference (province – CNISP)/CNISP | | | | | | | | | | | | | | | | | | | | | |  |  |  |  |  |  |  |  |  |  |  |  |  |  |  |  |  |  |  |  |  |  |  |  |  |  |  |  |  |  |  |  |  |  |  |  |  |  |  |  |  |  |  |  |  |  |  |  |  |  |  |  |  |  |  |  |  |  |  |  |  |  |
|  | Comparable | | | | | | | | | | | | | | | | | | | | | |  |  |  |  |  |  |  |  |  |  |  |  |  |  |  |  |  |  |  |  |  |  |  |  |  |  |  |  |  |  |  |  |  |  |  |  |  |  |  |  |  |  |  |  |  |  |  |  |  |  |  |  |  |  |  |  |  |  |  |  |  |  |
|  | Province not comparable with CNISP | | | | | | | | | | | | | | | | | | | | | |  |  |  |  |  |  |  |  |  |  |  |  |  |  |  |  |  |  |  |  |  |  |  |  |  |  |  |  |  |  |  |  |  |  |  |  |  |  |  |  |  |  |  |  |  |  |  |  |  |  |  |  |  |  |  |  |  |  |  |  |  |  |

**Table S10-** Data quality analysis for CDI: Proportion of occurrences over the 19 scenarios (representing 5 to 95% variation of one element) when the true value of the parameter (marginal proportion or OR) was outside the confidence interval calculated from 500 simulations

| Parameter | 2d | 3d | Fap | Di | Inf | Ad | Oy | Rec | H4w | Element 1 | | Element 2 | |  |  |
| --- | --- | --- | --- | --- | --- | --- | --- | --- | --- | --- | --- | --- | --- | --- | --- |
| OR 1 |  |  |  |  |  |  |  |  |  | 2d | | 3d | |  |  |
| OR 2 |  |  |  |  |  |  |  |  |  | 2d | | Fap | |  |  |
| OR 3 |  |  |  |  |  |  |  |  |  | 2d | | Di | |  |  |
| OR 4 |  |  |  |  |  |  |  |  |  | 2d | | Inf | |  |  |
| OR 5 |  |  |  |  |  |  |  |  |  | 2d | | Ad | |  |  |
| OR 6 |  |  |  |  |  |  |  |  |  | 2d | | Oy | |  |  |
| OR 7 |  |  |  |  |  |  |  |  |  | 2d | | Rec | |  |  |
| OR 8 |  |  |  |  |  |  |  |  |  | 2d | | H4w | |  |  |
| OR 9 |  |  |  |  |  |  |  |  |  | 3d | | Fap | |  |  |
| OR 10 |  |  |  |  |  |  |  |  |  | 3d | | Di | |  |  |
| OR 11 |  |  |  |  |  |  |  |  |  | 3d | | Inf | |  |  |
| OR 12 |  |  |  |  |  |  |  |  |  | 3d | | Ad | |  |  |
| OR 13 |  |  |  |  |  |  |  |  |  | 3d | | Oy | |  |  |
| OR 14 |  |  |  |  |  |  |  |  |  | 3d | | Rec | |  |  |
| OR 15 |  |  |  |  |  |  |  |  |  | 3d | | H4w | |  |  |
| OR 16 |  |  |  |  |  |  |  |  |  | Fap | | Di | |  |  |
| OR 17 |  |  |  |  |  |  |  |  |  | Fap | | Inf | |  |  |
| OR 18 |  |  |  |  |  |  |  |  |  | Fap | | Ad | |  |  |
| OR 19 |  |  |  |  |  |  |  |  |  | Fap | | Oy | |  |  |
| OR 20 |  |  |  |  |  |  |  |  |  | Fap | | Rec | |  |  |
| OR 21 |  |  |  |  |  |  |  |  |  | Fap | | H4w | |  |  |
| OR 22 |  |  |  |  |  |  |  |  |  | Di | | Inf | |  |  |
| OR 23 |  |  |  |  |  |  |  |  |  | Di | | Ad | |  |  |
| OR 24 |  |  |  |  |  |  |  |  |  | Di | | Oy | |  |  |
| OR 25 |  |  |  |  |  |  |  |  |  | Di | | Rec | |  |  |
| OR 26 |  |  |  |  |  |  |  |  |  | Di | | H4w | |  |  |
| OR 27 |  |  |  |  |  |  |  |  |  | Inf | | Ad | |  |  |
| OR 28 |  |  |  |  |  |  |  |  |  | Inf | | Oy | |  |  |
| OR 29 |  |  |  |  |  |  |  |  |  | Inf | | Rec | |  |  |
| OR 30 |  |  |  |  |  |  |  |  |  | Inf | | H4w | |  |  |
| OR 31 |  |  |  |  |  |  |  |  |  | Ad | | Oy | |  |  |
| OR 32 |  |  |  |  |  |  |  |  |  | Ad | | Rec | |  |  |
| OR 33 |  |  |  |  |  |  |  |  |  | Ad | | H4w | |  |  |
| OR 34 |  |  |  |  |  |  |  |  |  | Oy | | Rec | |  |  |
| OR 35 |  |  |  |  |  |  |  |  |  | Oy | | H4w | |  |  |
| OR 36 |  |  |  |  |  |  |  |  |  | Rec | | H4w | |  |  |
| 2d |  |  |  |  |  |  |  |  |  |  | |  | |  |  |
| 3d |  |  |  |  |  |  |  |  |  |  | |  | |  |  |
| Fap |  |  |  |  |  |  |  |  |  |  | |  | |  |  |
| Di |  |  |  |  |  |  |  |  |  |  | |  | |  |  |
| Inf |  |  |  |  |  |  |  |  |  |  | |  | |  |  |
| Ad |  |  |  |  |  |  |  |  |  |  | |  | |  |  |
| Oy |  |  |  |  |  |  |  |  |  |  | |  | |  |  |
| Rec |  |  |  |  |  |  |  |  |  |  | |  | |  |  |
| H4w |  |  |  |  |  |  |  |  |  |  | |  | |  |  |
|  |  |  |  |  |  |  |  |  |  |  | |  | |  |  |
| The theoretical proportion or OR was in the simulated confidence interval more than 80% of the 19 scenarios. | | | | | | | | | | | | | | | |
| The theoretical proportion or OR was not in the simulated confidence interval more than 80% of the 19 scenarios, but the theoretical OR was extreme (close to 0 or above 1000) and the lower or upper limit of the CI was extreme in an equivalent manner/direction in all 19 scenarios. | | | | | | | | | | | | | | | |
| The theoretical proportion or OR was not in the simulated confidence interval more than 80% of the 19 scenarios. | | | | | | | | | | | | | | | |
| Abbreviations: 2d, two days of admission; 3d, three days of admission; Ad, Admission; Di, Diarrhea; Fap, Fever and abdominal pain; H4w, Hospitalization within the last four weeks; Inf, Information on diarrhea available; Oy, One year or or more Rec, Recurrence between four and eight weeks | | | | | | | | | | | | | | | |
| Element 1: First element involved in the OR | | | | | | | | | | | | | | | |
| Element 2: Second element involved in the OR | | | | | |  |  |  |  |  |  | |  | |  |

**Table S11-** Relative difference between the theoretical value for the marginal proportion, and the furthest marginal proportion 95% CI limit obtained in simulation studies, for each element (left in the table) when varying the marginal proportion of elements at the top of the table from 5% to 95% (each cell presents the mean across all variations from 5 to 95% of the element at the top), CDI infections.^1^

|  | Element for which the marginal proportion varied from 5% to 95% in simulations | | | | | | | | |
| --- | --- | --- | --- | --- | --- | --- | --- | --- | --- |
| Element for which we assessed the marginal proportion | Ad | 2d | Fap | H4w | Inf | Rec | 3d | Di | Oy |
| Ad | 3,7% | 3,0% | 3,4% | 2,7% | 3,5% | 2,7% | 3,2% | 3,3% | 2,8% |
| 2d | 1,7% | 4,1% | 1,8% | 1,6% | 1,8% | 1,7% | 1,7% | 1,6% | 1,7% |
| Fap | 1,6% | 2,2% | 2,8% | 1,5% | 1,7% | 1,6% | 1,6% | 1,6% | 1,7% |
| H4w | 3,0% | 3,1% | 3,0% | 2,6% | 3,2% | 2,7% | 2,6% | 2,6% | 2,6% |
| Inf | 3,4% | 3,3% | 3,3% | 3,1% | 4,1% | 3,2% | 3,3% | 3,3% | 3,1% |
| Rec | 2,6% | 2,6% | 2,5% | 2,5% | 2,6% | 2,7% | 2,5% | 2,5% | 2,5% |
| 3d | 3,9% | 4,1% | 3,2% | 3,2% | 3,7% | 3,2% | 2,8% | 3,2% | 3,3% |
| Di | 2,6% | 8,4% | 9,1% | 2,4% | 2,5% | 2,4% | 9,1% | 2,6% | 2,4% |
| Oy | 1,2% | 1,3% | 0,9% | 0,9% | 1,2% | 0,8% | 0,9% | 0,8% | 2,7% |
|  |  |  |  |  |  |  |  |  |  |
| Abbreviations: 2d, two days of admission; 3d, three days of admission; Ad, Admission; Di, Diarrhea; Fap, Fever and abdominal pain; H4w, Hospitalization within the last four weeks; Inf, Information on diarrhea available; Oy, One year or or more Rec, Recurrence between four and eight weeks.  1. For instance, 3.7% in the first cell can be interpreted as on average when varying the proportion of the element ‘Admission’ from 5 to 95%, more than 95% of the marginal proportions of ‘Admission’ are contained within 3.7% of the theoretical (desired) value based on the multivariate model used in simulations. | | | | | | | | | |

**Table S12-** Data quality analysis for MRSA: Proportion of occurrences over the 19 scenarios (representing 5 to 95% variation of one element) when the true value of the parameter (marginal proportion or OR) was outside the confidence interval calculated from 500 simulations

| Parameter | 3d | Ms | Ad | Mmc | A7/90 | Ad/3 | Element 1 | Element 2 |  |
| --- | --- | --- | --- | --- | --- | --- | --- | --- | --- |
| OR 1 |  |  |  |  |  |  | 3d | Ms |  |
| OR 2 |  |  |  |  |  |  | 3d | Ad |  |
| OR 3 |  |  |  |  |  |  | 3d | Mmc |  |
| OR 4 |  |  |  |  |  |  | 3d | A7/90 |  |
| OR 5 |  |  |  |  |  |  | 3d | Ad/3 |  |
| OR 6 |  |  |  |  |  |  | Ms | Ad |  |
| OR 7 |  |  |  |  |  |  | Ms | Mmc |  |
| OR 8 |  |  |  |  |  |  | Ms | A7/90 |  |
| OR 9 |  |  |  |  |  |  | Ms | Ad/3 |  |
| OR 10 |  |  |  |  |  |  | Ad | Mmc |  |
| OR 11 |  |  |  |  |  |  | Ad | A7/90 |  |
| OR 12 |  |  |  |  |  |  | Ad | Ad/3 |  |
| OR 13 |  |  |  |  |  |  | Mmc | A7/90 |  |
| OR 14 |  |  |  |  |  |  | Mmc | Ad/3 |  |
| OR 15 |  |  |  |  |  |  | A7/90 | Ad/3 |  |
| 3d |  |  |  |  |  |  |  |  |  |
| Ms |  |  |  |  |  |  |  |  |  |
| Ad |  |  |  |  |  |  |  |  |  |
| Mmc |  |  |  |  |  |  |  |  |  |
| A7/90 |  |  |  |  |  |  |  |  |  |
| Ad/3 |  |  |  |  |  |  |  |  |  |
|  |  |  |  |  |  |  |  |  |  |
| The theoretical proportion or OR was in the simulated confidence interval more than 80% of the 19 scenarios. | | | | | | | | | |
| The theoretical proportion or OR was not in the simulated confidence interval more than 80% of the 19 scenarios, but the theoretical OR was extreme (close to 0 or above 1000) and the lower or upper limit of the CI was extreme in an equivalent manner/direction in all 19 scenarios. | | | | | | | | | |
| The theoretical proportion or OR was not in the simulated confidence interval more than 80% of the 19 scenarios. | | | | | | | | | |
| Abbreviations: 3d, three days of admission; A7/90, Previous admission withing the last 7 or up to 90 days; Ad, Admission; Ad/3, Previous admission within the last three days; Mmc, Mental or mother and children patients; Ms, Mother status; | | | | | | | | | |
| Element 1: First element involved in the OR | | | | | | | | | |
| Element 2: Second element involved in the OR | | | | | | | | | |

**Table S13-** Relative difference between the theoretical value for the marginal proportion, and the furthest marginal proportion 95% CI limit obtained in simulation studies, for each element (left in the table) when varying the marginal proportion of elements at the top of the table from 5% to 95% (each cell presents the mean across all variations from 5 to 95% of the element at the top), MRSA infections.^1^

|  | Element for which the marginal proportion varied from 5% to 95% in simulations | | | | | |
| --- | --- | --- | --- | --- | --- | --- |
| Element for which we assessed the marginal proportion | 3d | Ms | Ad | Mmc | A7/90 | Ad/3 |
| 3d | 3,2% | 6,2% | 5,9% | 3,8% | 3,4% | 3,3% |
| Ms | 4,4% | 4,3% | 4,7% | 3,5% | 3,4% | 4,5% |
| Ad | 2,0% | 3,2% | 2,2% | 1,6% | 1,7% | 2,0% |
| Mmc | 3,1% | 3,8% | 3,6% | 3,4% | 2,8% | 3,2% |
| A7/90 | 1,1% | 1,2% | 1,4% | 1,1% | 1,1% | 2,8% |
| Ad/3 | 2,7% | 4,8% | 3,3% | 3,1% | 3,2% | 3,0% |
|  |  |  |  |  |  |  |
| Abbreviations: 3d, three days of admission; A7/90, Previous admission withing the last 7 or up to 90 days; Ad, Admission; Ad/3, Previous admission within the last three days; Mmc, Mental or mother and children patients; Ms, Mother status.  1. For instance, 3.2% in the first cell can be interpreted as on average when varying the proportion of the element ‘three days’ from 5 to 95%, more than 95% of the marginal proportions of ‘three days’ are contained within 3.2% of the theoretical (desired) value based on the multivariate model used in simulations. | | | | | | |
